# Deep neural network prediction of genome-wide transcriptome signatures – beyond the Black-box

**DOI:** 10.1038/s41540-022-00218-9

**Published:** 2022-02-23

**Authors:** Rasmus Magnusson, Jesper N. Tegnér, Mika Gustafsson

**Affiliations:** 1 Bioinformatics, Department of Physics, Chemistry and Biology, Linköping University, Linköping, Sweden; 2 School of Bioscience, Systems Biology Research Center, University of Skövde, Skövde, Sweden; 3 Biological and Environmental Sciences and Engineering Division, Computer, Electrical and Mathematical Sciences and Engineering Division, King Abdullah University of Science and Technology (KAUST), Thuwal, 23955–6900 Saudi Arabia; 4 Unit of Computational Medicine, Department of Medicine, Solna, Center for Molecular Medicine, Karolinska Institutet, Stockholm, Sweden; 5 Science for Life Laboratory, Solna, Sweden

**Keywords:** Regulatory networks, Biomarkers, Computer modelling

## Abstract

Prediction algorithms for protein or gene structures, including transcription factor binding from sequence information, have been transformative in understanding gene regulation. Here we ask whether human transcriptomic profiles can be predicted solely from the expression of transcription factors (TFs). We find that the expression of 1600 TFs can explain >95% of the variance in 25,000 genes. Using the light-up technique to inspect the trained NN, we find an over-representation of known TF-gene regulations. Furthermore, the learned prediction network has a hierarchical organization. A smaller set of around 125 core TFs could explain close to 80% of the variance. Interestingly, reducing the number of TFs below 500 induces a rapid decline in prediction performance. Next, we evaluated the prediction model using transcriptional data from 22 human diseases. The TFs were sufficient to predict the dysregulation of the target genes (rho = 0.61, *P* < 10^−216^). By inspecting the model, key causative TFs could be extracted for subsequent validation using disease-associated genetic variants. We demonstrate a methodology for constructing an interpretable neural network predictor, where analyses of the predictors identified key TFs that were inducing transcriptional changes during disease.

## Introduction

Bridging the gap between genome sequences and phenotypes is a core challenge in genomics and personalized medicine. To this end, it is essential to characterize intermediate levels, such as cells, tissues, and organs, using a suite of molecular technologies. For example, genetic variants associated with diseases exert their effects through the modulation of these intermediate levels. The transcribed mRNA expression is one of the most accessible and important windows into the cell’s regulatory machinery and changes in tissues and organs. Therefore, the analysis of mRNA expression is crucial for the study of diseases^1^. Specifically, the elucidation of gene regulatory mechanisms is central since gene regulatory networks maintain cellular identity and mediate interactions between genetic variants and the environment of humans.

To reverse-engineer gene regulatory mechanisms, large amounts of RNA expression data have been generated from experimental model systems, including cell-lines and tissues from humans under different conditions. Among others, the recount2^2^, the Genotype-Tissue Expression (GTEx) project^3^, and the ARCHS4 database^4^ have all made great amounts of data available. Bioinformatics analysis has been instrumental in clustering genes to make sense of such data and augmenting the power for hypothesizing putative genes involved in diseases^5^. Enrichment and pathway analysis increase the resolution by suggesting groups of genes or specific pathways associated with the observed changes in gene expression. Since transcription factors (TFs) are critical for the regulatory control of genes, a massive body of bioinformatics tools targets TF binding sites’ predictions, suggesting key drivers behind pathways, groups of genes, or clusters^6^. While useful for descriptive purposes, such as associating such differentially expressed genes to many diseases, it is challenging to gain functional and mechanistic insight into the regulatory machinery from such lists.

To advance beyond lists, clusters, and enrichment analysis, a complementary strategy, referred to as network science, instead targets the study of interactions between molecular entities, genotypes, and phenotypes^7,8^, For example, gene regulation effectively acts via a network of interacting genes^9^. Notably, genes that interact with dysregulated genes without being differentially expressed themselves are often overlooked in differential expression studies^10^. Consequently, these networks are challenging to extract from data^11^. The wisdom of the crowd strategy has turned out to be useful while not satisfactory^12^. The limited progress originates from the fact that the inverse problem of inferring interactions from observations is statistically under-constrained. Moreover, these approaches have all struggled with the complex and non-linear dynamics that shape gene regulation, containing several saturation effects and abundant negative and positive feed-backs. These non-linearities impede most of the available correlation-based methods used to study gene expression^13^.

Recent progress in machine learning has fueled interest in whether such methods could facilitate the discovery and analysis of biological networks^14,15^, Pioneering applications of deep neural networks (DNNs) in genomics include prediction of TF binding sites^16^) and the effects of non-coding genetic variants^17^. At the core of these techniques is the ability to capture non-linear relationships. The use of DNNs requires substantial amounts of data, which is now feasible due to the collection of massive amounts of genetic and RNA-seq data into easily accessible databases^4^. Beyond detection of features, recent use of DNN, such as deep autoencoders applied to transcription data, compresses gene expression data into a latent space. The original data can then be reconstructed from the latent space representation^18,19^, DNNs have also been applied to understand the regulation of mRNA expression. Deep convolutional neural networks could predict 60-80% of human RNA abundance variation from the genomic sequence alone^20,21^, While being the first important step towards predicting mRNA levels, the regulatory transcription factors were not separated from the remaining transcriptome, making a biological interpretation and translation to diseases challenging.

Here we develop a methodology that goes beyond producing lists of differentially expressed genes, but not so far as the yet intractable reconstruction of a complete gene regulatory network. Instead, we target the regulation exerted by the transcription factors and ask whether training a DNN on gene expression data could learn a predictive TF network. The expression of all other genes could be explained by the combinatorial control induced by the TFs. Importantly, we constraint the training such that the resulting predictive model is interpretable. We refer to this as a methodology for advancing beyond black-box machine learning models, which in turn is a first step towards what could be referred to as white-box fully interpretable models. We find that such models can indeed predict the expression of genes based on TFs and that the predicted relationships between TFs and their target genes largely overlap with known TF bindings. We apply and evaluate this predictive model using human disease transcriptomes, thus opening the door for a mechanistic and interpretable machine learning analysis of the human gene regulation system.

## Results

First, we explore the influence of different neural network architectures, such as the number of layers and hidden nodes, on predictive performance. Next, we ask how we can inspect the trained network and disentangle the different predictors’ contributions (TFs). Using a light-up network analysis technique, we identify a core set of TFs, including some key regulators. The final section of the results asks whether the prediction model could analyze disease-derived transcriptional data.

### Accurate and robust prediction of the expression level of target genes using deep neural transcription factor networks

**Fig. 1:**
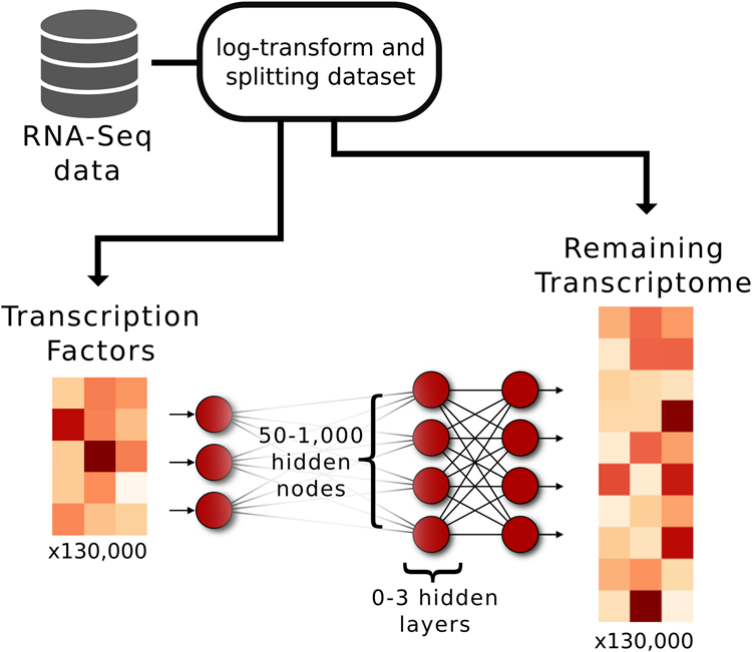
Design of the artificial deep neural networks. The networks were made to predict the expression of 25,861 genes based on 1,625 gene regulators, i.e. transcription factors (TFs). We used more than 100,000 randomly drawn RNA-seq samples from the ARCHS4 database to train the models. Moreover, we designed 15 DNNs of 1–3 hidden layers, and one shallow neural network without any hidden layers. For the DNNs, each hidden layer consisted of either 50, 100, 250, 500, or 1000 hidden nodes using the exponential linear unit, ELU as activation function.

**Fig. 2:**
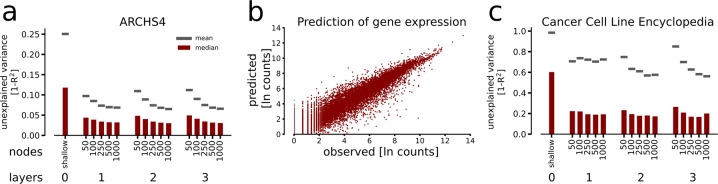
Gene expression prediction performance. **a** We applied the DNNs and the shallow model to previously unseen gene expression profiles randomly selected from the ARCHS4 database, and calculated the coefficient of determination, *R*^2^, for each gene. Shown are the median (red bar) and mean (gray line) of the *R*^2^ values for each model. **b** The typical prediction of an experiment is shown for the DNN with two hidden layers and 250 hidden nodes in each layer. **c** We applied the DNNs to predict gene expression values from 934 human tumor samples from the Cancer Cell Line Encyclopedia. Shown are the abilities to predict this data set for the respective models, following the same layout as in **a**).

To optimize the applied Deep Neural Network (DNN) performance, we compared 15 DNN architectures consisting of 1–3 intermediate layers, each with a depth of 50–1000 hidden nodes. As a reference, we used a shallow NN without any intermediate layer (Fig. 1). The rationale is to identify the most compact architecture, measured by depth and width, capable of predicting most of the target gene expression with sufficient accuracy. Here, TFs were used exclusively as input and non-TFs as output target genes. We extracted TFs using the compendium provided by Lambert et al. 2018^22^, which lists TFs based on several sources, including popular TF databases such as TRANSFAC, JASPAR, and HT-SELEX. We trained models using the ARCHS4 database, using more than 100,000 randomly drawn RNA-seq samples to train and evaluate the models^4^. The performance was evaluated using the gene-specific coefficient of determination (presented as 1 - *R*^2^) on test data. We observed median 1 - *R*^2^ values between 0.12 and 0.03 depending on the model (Fig. 2a, b). Similarly, the mean 1 - *R*^2^ values of all DNNs were in the range of 0.11–0.07, whereas the shallow model had a notably worse performance (1 - *R*^2^ = 0.25). A gene-specific list of the *R*^2^-coefficients, together with the corresponding Spearman correlations, can be found in Supplemental Table 1. Since the RNA-seq gene counts contain several intercept terms, such as sequencing depth and mean gene expressions, we tested the 1 - *R*^2^ when the input data were randomly permuted and found the mean 1 - *R*^2^ to be on the range of (0.79–0.67). Thus, we observed an increase in the ability to predict gene expression compared to 80% of the explained variance when predicting mRNA abundance solely from the DNA sequence, as Zrimec et al. 2020^21^ reported.

To test the trained models’ generalizability, we applied them to predict expression from the independent Cancer Cell Line Encyclopedia resource^23^, which contains mRNA profiles of 934 human tumor cell lines. In this out-of-sample prediction, the DNNs showed a high median 1 - *R*^2^ between 0.26 and 0.17, whereas the shallow model only reached a median *R*^2^ = 0.60. The mean 1 - *R*^2^ values were lower than the medians for all models (0.85–0.56), which we speculate is due to the inclusive list of target genes, such that genes with low variance deflate the mean 1 - *R*^2^. As a comparison, the top 25,000 genes had a mean 1 - *R*^2^ of *<* 0.25 in all deep models. With the input data randomly permuted, we observed the mean 1 - *R*^2^ in the range of (1.41–1.06). The non-linear DNNs could capture perturbations almost three-fold better than the shallow NN, suggesting their potential usefulness even in cancer medicine studies. We continued studying the predictive power of our approach on expression in individual cell types, and found a the learning to have captured a broad representation of human tissues (Supplemental Material 1). With these results, we concluded that the DNNs could faithfully predict the majority of the human transcriptome given the expression levels of ∼1600 TFs, both in healthy and disease-affected states.

### The node light-up technique revealed enrichment of validated TF-target associations within the prediction networks

**Fig. 3:**
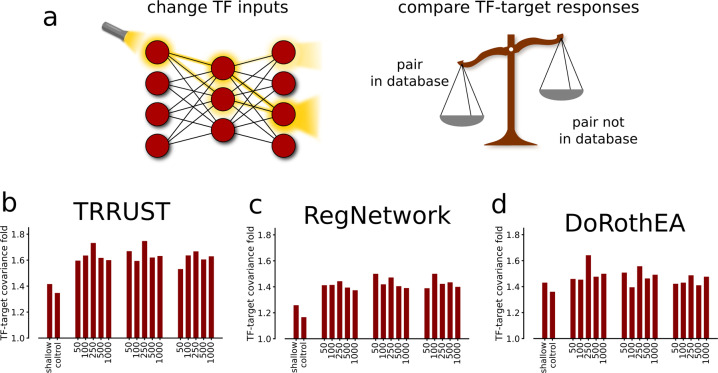
Node light-up reveals known TF-target associations. **a** By applying a light-up analysis, i.e. by changing the input values of each TF independently and subsequently observing the changes on the output layer, we were able to estimate how the TF-to-target mappings corresponded to TF bindings known from literature. We defined the TF-target covariance fold as the median value of the light-ups for the TF-target regulations found in a database divided by the respective backgrounds. **b** The light-up enrichments for the TF-target associations found in the TRRUST database are shown. Note that the expected value representing no biological relevance between TF-target mapping is 1 and that light-up values are compared to absolute Spearman rank correlation values, labeled ‘control’. We performed the same analysis with RegNetwork (**c**), and the interactions annotated with top confidence in DoRothEA (**d**).

Here we ask whether the trained networks are interpretable, that is, whether the learned TF target associations are biologically relevant or not. From a biological standpoint, one could compare directly with known physical DNA bindings between TFs and their target genes. However, due to the nonlinear dependencies embedded in a DNN, such an analysis is not trivial^24^. For example, even simple DNNs can have millions of parameters, and all input values can potentially impact every output value. To approximate the learned dependencies between TFs and target genes, we, therefore, reasoned that in the predicted network, the TF expression perturbations would propagate most effectively to relevant target genes. In other words, such an analysis captures the effective gene dependencies, linear or non-linear. To this end, we used a light-up node analysis, following the implementation in^18^. In other words, the numerical value of each input node, corresponding to a unique TF, was independently perturbed to either half or double of the mean gene expression. The other TF expression values were clamped to their average values, while the responses on the output layer were ranked by the response to such a TF change (Fig. 3a). To validate the DNNs, we compared the light-up values to previously known TF-target bindings. Specifically, we tested whether the light-up responses between such TF-target pairs were significantly higher than the pairs not annotated as interactions.

To this end, we used four sources of TF-target interactions. Specifically, the TF-specific DoRothEA database (*n*_TFs_ = 94, *n*_targets_ = 2240, *n*_edges_ = 4498), the ReMap dataset (n_TFs_ = 296, n_targets_ = 15,196, n_edges_ = 269,757), and the TRRUST database, which has a larger TF coverage (n_TFs_ = 481, n_targets_ = 1,961, n_edges_ = 6,576). The DoRothEA is based on manually curated TF-target interactions, and ChIP-seq derived measurements^25^, the ReMap interactions are derived only from ChIP-seq measurements^26^. The TRRUST database is an extraction of small-scale experimental analyses of TF regulations^27^. We also included the RegNetwork database^28^, which is a compilation of 25 commonly used TF-target binding databases and thus includes numerous additional interactions of somewhat less confidence (*n*_TFs_ = 645, *n*_targets_ = 14,696, *n*_edges_ = 99,488). We also noted a limited overlap between these three databases. For example, 927 interactions were shared between the TRRUST and DoRothEA sets, 1262 between DoRothEA and RegNetwork, 645 between ReMap and TRRUST, and 751 between RegNetwork and TRRUST. Strikingly, the light-up responses between known TF-target associations were significantly higher (26–75%) for all manually curated databases and DNNs (Fig. 3b–d). The more inclusive ReMap also displayed higher light-up values but at lower levels (5-8%). Thus, all models performed better than what was expected under the null hypothesis (60 of 60, binomial *P* < 10^−19^). While the curated databases’ results did not point to a single best model architecture, we observed the highest overall performance for the DNN with two hidden layers using 250 nodes in each layer. The highest enrichment rank was obtained from TRRUST, while DoRothEA yielded the second-highest, and RegNetwork the lowest rank.

Since TFs can act as both inhibitors and initiators of transcription, we asked the extent to which the DNN light-up analyses also captured the directionality and sign of the TF-target interactions. Therefore, we compared the light-ups with known interactions, directions, and signs of TRRUST and DoRothEA. Of note, RegNetwork does not contain any annotation of interaction signs. Again, all tested DNNs showed significant overlaps with the interaction signs of both databases, with accuracies ranging between [0.62–0.67] (P ∈ [10^−120^,10^−58^]) for DoRothEA, and [0.56–0.58] (*P* ∈ [10^−22^,10^−11^]) for TRRUST. We provide the average precision- and receiver operating characteristic scores, with the corresponding figures, in the Supplemental Material 2. We defined accuracy as the percentage of correctly estimated signs of interaction. Again, the shallow model performed significantly worse and could only predict interaction signs compared to DoRothEA (accuracy= 0.57, *P* < 10^−20^), while the comparison to TRRUST gave insignificant results.

In general, we find that the number of hidden layers or units for the DNNs has only a limited impact on the performance. All DNN models showed comparable performance across evaluative analysis, expression predictions of experiments from the ARCHS4 database, the cancer expression predictions, and the light-up comparison to TF-target databases. In practice, 250 hidden nodes in two hidden layers appeared to be a sufficient model size. Notably, the shallow NN never reached a satisfactory performance. Thus, the largest gain in explanatory power and overlap with existing databases came in our hands from adding at least one intermediate layer, thereby allowing for non-linear transformations. While these non-linearities turned out to be essential for the performance, they did not prevent us from inspecting the predictive network and extract relevant and validated biological knowledge using the light-up techniques.

### Algorithmic extraction of a core set of validated regulator TFs from the DNN

We next searched for a minimal subset of key TFs required for predicting the target gene expressions. For this task, we implemented a backward-selection algorithm to stepwise remove TFs from the input layer based on their explanatory power (Methods). We observed highly consistent orders of TF-removal between the models, again suggesting that the TF-target relationship is robust to different DNN architectures. Indeed, the median correlation between different models, estimated from which step the TFs were removed, was 0.71 (geometric mean *P* < 10^−364^). This suggests a high consistency between the independently trained models (Supplemental Material 3). This observation, taken together with the light-up analysis outcome, suggests robustness of our results in that specifics of a DNN have only a minor influence on the results. Therefore, we used the model with two hidden layers and 250 hidden nodes in the analysis’ remaining parts. The step at which each gene was removed is presented in Supplemental Table 2.

**Fig. 4:**
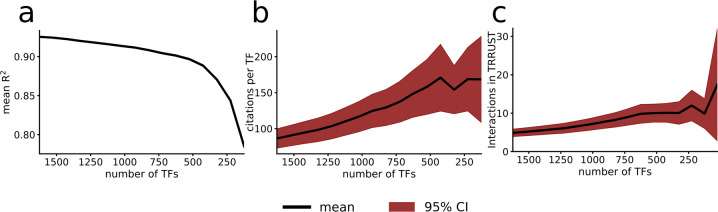
Functional Hierarchies in Model Input. **a** We found the explanatory power on test data to decrease as the number of TFs were removed from the model. Particularly, we found around 500 TFs to carry a greater predictive power, as the explanatory power after that point more rapidly decreased. **b** We measured the median number of scientific studies at each iteration of our backward-selection algorithm, and found that as the number of input TFs decreased, the more studied were those that were left. **c** The TFs that were identified as more important also had more recorded interactions in the TRRUST database.

As expected, the explanatory power gradually decreases when removing predictors, here TFs (Fig. 4a). However, the shape of this loss of explanatory power exhibited two distinguishing features. First, the ability to predict mRNA expression remained relatively unperturbed even as most TFs were removed from the set. Indeed, for *n*_TFs_ = 125, the mean 1 - *R*^2^ = 0.21 compared to that of the full model of 1,625 TFs, which measured 0.07. Second, there appeared to be a stratification of TFs based on how important they are to explain the system, leading to a larger reduction in explanatory power towards the procedures‘ late stage.

To test whether our predicted core set of TFs could correspond to known, important regulatory TFs, we estimated how often or much they have been studied in the literature domain. First, we analyzed the average number of scientific publications per input TF, as annotated by PubMed^29^, and found that the top predicted TFs were significantly more frequently studied (Fig. 4b). For example, the median number of publications was 51 for the top 125 TFs, compared to 24 for all TFs (Mann-Whitney *U* test *P* < 8.2*10^−9^). Second, we analyzed the light-up conformity to the TRRUST database, but this time for each step in the backward-selection algorithm. We found that the full model performed equally well or better at next-to-all steps in the backward-selection algorithm. This observation indicates that the model predominantly discovered known TF-target interactions regardless of model input size. Third, the number of annotated interactions per TF was significantly larger for the top-ranked TFs in the literature-based database TRRUST (9.9 for top 125 compared to 4.9, *P* < 2.4 * 10^−6^), suggesting that the top explanatory TFs were associated with more well-known regulatory bindings. We next analyzed the functional role of the removed TFs and performed enrichment analysis of their predicted downstream targets from our light-up technique (Methods). For each set of 100 removed TFs using the backward-selection procedure, we associated their top 500 target genes, which were then subjected to a KEGG pathway^30^ enrichment analysis. In brief, we found that the top 100 TFs (i.e., the last removed TFs) were mostly enriched for cellular metabolism pathways, followed by immune-pathways for the TFs with rank 100–200 (Supplemental Table 3). Narrowing down further, we analyzed the 25 TFs preserved in our very last step in and a Pubmed query using the search term “master regulator” together with each of the TFs and found that 13 were co-localized with that search term in 358 unique articles, which supports the notion that these are true upstream regulators. Thus, despite having abundant input variables, the DNN discovered known core TFs associated with central and well-annotated pathways.

### Latent DNN space shows enrichment of functionally related and disease-associated genes

In addition to assessing the biological relevance of the specific interactions discovered by the predictive DNN, one may ask whether the predictor could be informative in a disease context. It has been demonstrated that compressing mRNA expression data in deep autoencoders can provide low-dimensional representations exposing complex characteristics of the input data domain. Importantly, from such a representation, one can readily extract sets of functionally related genes, known as modules, which in turn can be used to study diseases^18^. To further increase the resolution beyond the analysis of modules, here we aimed to use the TF-target gene interactions within the two 250 variables measuring intermediate latent layers.

Specifically, we tested whether the genes associated with these hidden variables shared cellular functionality. For this purpose, we again used a light-up response to associate genes to each hidden node independently. We annotated the top 500 responding genes to each node light-up by this procedure and performed a Reactome pathway enrichment analysis for each node-set. This procedure identified 175 unique Reactome pathways that significantly overlapped with at least one hidden node (using a Bonferroni correction of 0.05). In detail, 102 hidden nodes of the first and 162 of the second layer were associated with at least one Reactome pathway. These results suggested at least a subset of the hidden nodes represented different cellular pathways. We evaluated the putative agglomeration of disease-related genes in the hidden-layer light-up responses. To this end, using each hidden node, we performed a genome-wide association study (GWAS) enrichment analysis between annotations in the NHGRI-EBI GWAS catalog^31^ and the 500 genes with the highest light-up associations. We found 37 of the 153 diseases associated with one or several nodes, with 63 nodes in the first and 62 nodes in the second layer having at least one association. Second, we cross-checked these results by comparing the light-ups with the DisGeNET database^32^, which contains broader profiles of gene-disease associations. We tested against genes grouped in 26 disease-type categories and found 22 of these categories enriched with at least one hidden node. At least one significant overlap with a disease category was detected in 107 nodes in the first and 138 in the second layer. Thus, disease-genes appeared to co-occur in hidden-node light-ups in the DNN. This result suggests that the remaining genes found in such disease modules could be relevant in analyzing and interpreting disease-related biomarkers and mechanisms.

### DNN analysis gives insights to human disease mechanisms involved in gene dysregulation

**Fig. 5:**
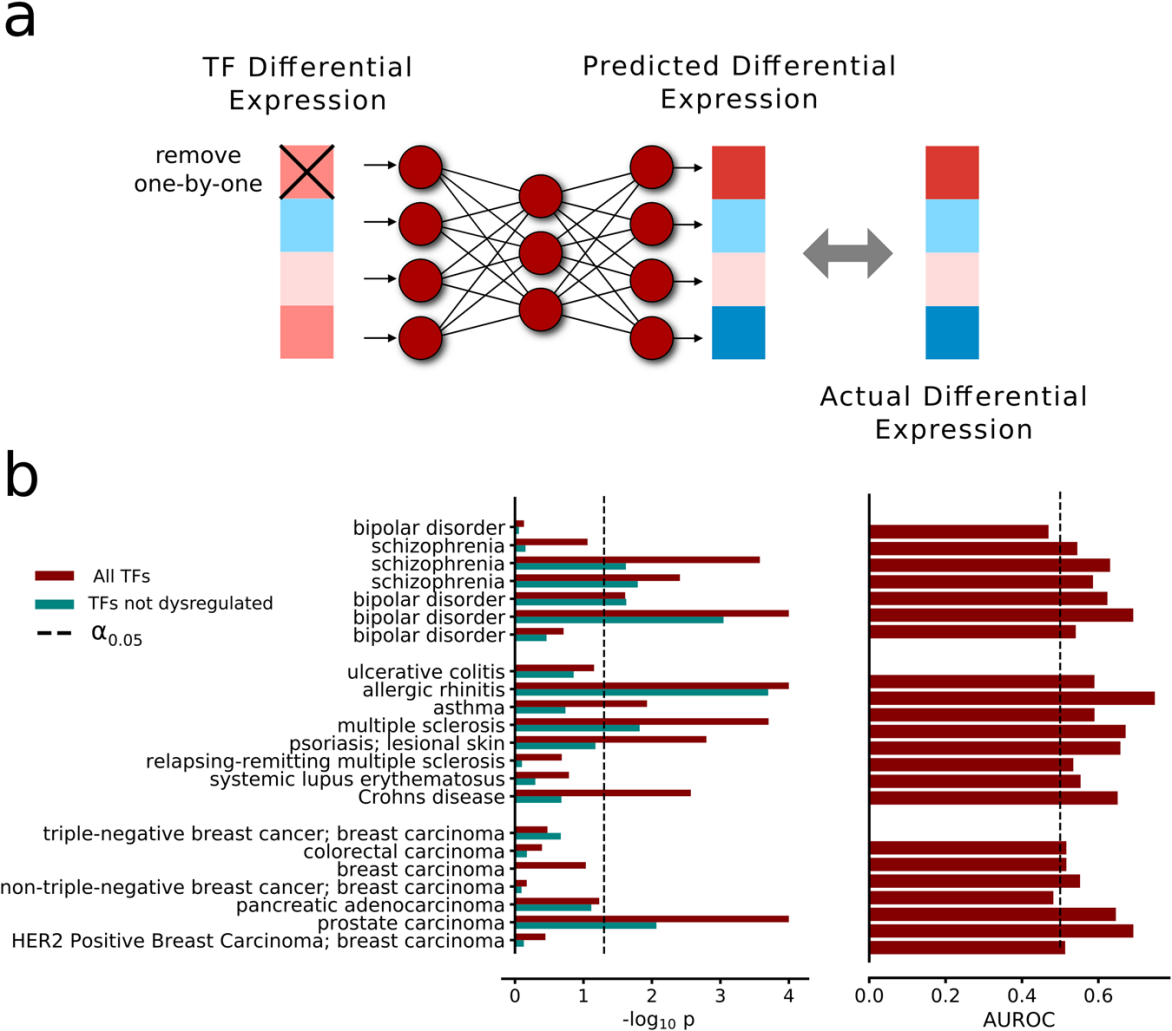
Predicting gene dysregulations in disease using a DNN. **a** We tested if the DNN with two hidden layers of 250 hidden nodes each could be used to predict causative changes in disease states. We did this by analyzing gene expression changes from known diseases, as available in the Expression Atlas repository. By applying the disease changes to the transcription factor input layer, we could observe how these changes projected down to the target genes. Next, we removed the disease-fold changes of each TF independently and observed the changes in correlation between predicted and observed dysregulation of the target genes. Thus, we could rank the TFs on predicted causative disease changes on the target genes. **b** TF rankings significantly overlapped with GWAS in 10 of 22 diseases. (−*log*_10_ *P* values shown as red bars). The test was repeated with significantly differentially expressed TFs removed from the set, leaving 7 TF rankings to overlap with GWAS. (−*log*_10_ *P* values shown as teal bars). The corresponding area under the curves for the TF rankings (all TFs) are shown to the right, with 20 of 22 diseases having an AUROC greater than the expected, as generated under the null hypothesis.

We finally aimed to test the clinical relevance of the DNN by using it to study disease-related changes in the expression of target genes, given the corresponding modifications of TF levels. To this end, we applied the DNN to independent RNA-seq data from the Expression Atlas^33^ using differential expression patterns for diseases from 27 different studies containing 69 expression fold profiles. We tested if the DNN could predict the differential expression of target genes given the fold profiles of the TFs. Prediction quality was measured as the correlation between the predicted and observed fold ranking of significantly differentially expressed target genes for each disease. In other words, we set the TFs to their reference expression levels and applied the fold changes of each respective disease. We next calculated the correlation between the observed and predicted fold changes at the output layer. Notably, we observed highly significant correlations between these predictions and observations, with a median Spearman rank correlation of 0.61 (median *P* < 10^−216^). This result established that disease mechanisms of dysregulation from TFs could be faithfully propagated to the target level. Yet, as a correlation does not imply causation, we assessed whether the DNN could be used to disentangle which TFs drive the target dysregulation. To rank the impact of a predicted TFs, we replaced dysregulated genes with reference expression values independently for each TF. By this, we could evaluate the impact on the output layer and therefore use the change in correlation between predicted and observed target dysregulation (Fig. 5) as a basis for the tanking. Next, we matched these rankings to known genetic variants from genome-wide association studies (GWAS) in the 22 applicable cases and measured the area under the receiver operating characteristics curve (AUROC). The TF rankings in 10 out of 22 diseases significantly matched the TF GWAS annotation (false discovery rate = 0.11, 10/22 binomial test *P* < 3.6 * 10^−8^). Furthermore, we found 20 of 22 diseases to have an AUROC larger than 0.5, i.e., the value expected under the null hypothesis (binomial test *P* < 6.0 * 10^−8^).

Finally, we asked if TFs had to be differentially expressed to carry predictive power over disease-affected target gene regulation. This question is particularly important since causative disease-related changes are not necessarily manifested through dysregulations that are large enough to be detected in multiple-testing corrected statistical tests of expression changes. Notably, we found our approach of causative predictions on a TF level to also give significant results when only applied to TFs that themselves were not significantly dysregulated, with seven diseases remaining significant (Fig. 5, *P* < 6.8 * 10^−5^). In other words, the removal of differential expression of TFs could predict disease-relevant TFs, even if the change in mRNA levels was modest. This suggests that our approach is generally applicable to find disease-causative elements at the TF-level, beyond what a more conventional RNA-seq analysis of gene expression detects. In summary, the TF-to-target DNN allowed a simple representation to describe the observed differential expression between patients of many different complex diseases and control in terms of TFs, which strikingly also correctly associated a truly enriched fraction of disease-associated TFs to several diseases.

## Discussion

We have presented a biologically interpretable, general machine learning method for predicting transcriptomic signatures, including disease signatures. Our learned models predict the expression of genes from the expression of transcription factors (TFs). The predicted relationships between TFs and their target genes largely overlap with known TF bindings. Hence, our DNN method goes beyond classic descriptive bioinformatic techniques such as clustering and enrichment analysis. Importantly, we do not address the yet intractable problem of complete deconvolution of the entire cellular interactome. Instead, our method does extract a core TF component out of such complex regulatory architecture. Therefore, the presented methodology sets the stage for the first step towards mechanistic and interpretable data-driven machine learning analysis of the human gene regulation system.

The early success of machine learning techniques targeted fundamental open bioinformatics problems such as predicting binding sites of TFs^16^ or functional prediction of non-coding RNA in a disease context^17^). The recent extension has focused on questions such as if one data type can be predicted from another datatype. Predicting gene expression from the DNA sequence or prediction of the 3D genomic structure from open chromatin data are just two recent examples. Tasaki et al.^34^ used deep convolutional neural networks to predict differential expression based on mRNA features and the promoter regions of the genes. The L1000 project deals directly with the prediction of gene expression from a smaller subset of genes, where measurements of 1000 landmark genes are used to infer 80% of the transcriptome^35^ computationally. Yet, here as in the other examples, these impressive modeling advances are difficult to translate into biological knowledge. As in different machine learning areas, these systems are useful predictors but act in practice as black-box systems. Related to this challenge is that even if functionally relevant representations can be identified, they can readily suffer from study-biases of identified prevalent genes. The remaining core challenges in using machine learning techniques include the interpretability of the model, large data requirement, and how to learn biologically meaningful representations within the machine learning model. A black-box model does not lend itself to interpretable and meaningful representations, potentially making the model more susceptible to adversarial attacks^36,37^, Recently, it has become increasingly clear that deep neural networks (DNNs) have the potential to identify biologically meaningful molecular representations directly from data^18,38^, and to revolutionize medicine^39,40^, It is therefore of critical importance for the field to develop techniques supporting biological interpretation and insights from such predictive models.

Our main contribution is to design a constrained machine learning approach such that the predictor is interpretable from a biological standpoint. Using two hidden layers with 250 hidden nodes each was sufficient to capture well-known TF-target pairs. These results suggest that the learned representation has significant architectural overlap compared to a proper cellular control system, which is essential for the good generalizability of DNNs. By developing a back-tracking algorithm, we could uncover a minimal core set of 125 TFs sufficient to account for ∼80% of the transcriptomics signature. Interestingly, these TFs represent the TFs being studied most frequently, which makes further studies linking prior structural information from other data types a logical next step to increase generalizability.

Our focus on TFs originates from the observation that TFs have been at the forefront in analyzing cellular reprogramming and converting cell-types^41,42^, Our findings suggest that the presented DNN methodology could be useful as a general method for predictive but yet interpretable studies. In particular to pinpoint key candidate TFs for cellular reprogramming using large transcriptomics data^43^. One of the most common approaches to analyzing disease mechanisms is studying gene expression changes between healthy and disease-affected individuals. Traditionally, a suite of bioinformatics descriptive mining techniques is applied to extract putative candidates of interest^5^. Nevertheless, it has proven challenging to pinpoint molecular mechanisms with high precision using such data-mining approaches. Consider a scenario where the impact of a perturbation on the gene regulatory system is of interest. Such cases are a common end-goal of analyzing transcriptomics and pivotal to understanding mechanisms such as drug perturbations or cancer impacts^44^. Using our light-up analyses could help predict specific regulatory interactions and their effect on the transcriptome. This could be useful both for cellular reprogramming experiments as well as in the analysis of diseases. In contrast, a machine learning model using only gene sequences would not readily consider such changes.

Lastly, since our predictor’s architecture has a biological interpretation, it could be used as a first approximation – like a blueprint – of the regulatory networks controlling the cell-identity and filtering effects of genetic variants. This problem has been at the forefront in systems biology since sequencing the human genome^12,45^, Yet, despite two decades of brilliant work on reverse-engineering gene regulatory networks from data, it remains an open challenge^46^. The combinatorial complexity of such a network exceeds the amount and quality of available data given the current suite of models^12^. As network predictions vary between methods, new tools have been developed to control the abundance of false interactions^47^, illustrating that the problem is still outstanding. Yet, if robust methods, such as our proposed DNN technique, could elucidate the TF part of such a network, we could potentially approach the problem in a step-wise manner. Hence, in summary, a TF-centric reverse-engineering technique could therefore be a stepping stone for renewed systems biology efforts in elucidating the cellar regulatory machinery at scale.

## Materials and Methods

### Data processing

We trained the models on gene expression data from ARCHS4, a database of *>*130,000 human RNA-seq gene expression measurements from GEO and SRA. We first separated the data into two sets, one of the genes annotated as TFs and one containing the rest of the genes, which were assumed to be regulated downstream of the TFs. We defined a TF as genes identified by Lambert et al.^22^, *The Human Transcription Factors*, which lists TFs based on TF databases such as TRANSFAC, JASPAR, and HT-SELEX. We defined the target genes, such as all the remaining genes, excluding pseudo-genes.

Next, we divided the data into 100 comma-separated files to be randomly accessed during the subsequent model training. Moreover, we removed 1200 gene expression profiles from the training set to use as validation of the model predictions. We normalized the data by applying the natural logarithm to the expression counts, annotated as x in Eq. (1).

1 $${\it{x}}_{normalized} = {{{\mathrm{ln}}}}(x + 1)$$

### Model design

We next aimed to predict the expression levels of the target genes using the TF levels. To this end, we designed the models to be feed-forward, fully connected neural networks. We built the models to have one input node for each TF, totaling 1525 input nodes and one output node for each target gene in the data, totaling 25,861 output nodes. Moreover, we opted to use the exponential linear unit (ELU) activation function on all nodes and across all layers. We used the Adam algorithm to minimize the mean squared error, with a learning rate of 0.001, parameter *beta*_1_ = 0.9, *beta*_2_ = 0.999, and decay of 0.01. The models were trained in the Keras package for Python 3, where we used a batch size of 50 experiments and with a validation split of 0.1. The code is available at https://github.com/rasma774/tf_target_white_box_dl.

### Model light-up analysis

NNs are complex, non-linear models, and mapping input to output is not trivial. Here, we aimed to extract TF-target relationships TF-by-TF via comparing the model output between I) the output when the mean TF expression is given as input and II) when each TF has a doubled and halved expression. The rationale behind this approach is that targets that are dependent on a particular TF will have a greater response when the input value of this TF is altered, as compared to unrelated TFs. We next compared these responses to databases of known TF-target interactions, such that for each target, we divided the median of the light-up values for TFs that were known to regulate the target with the median of the rest of the TFs. In other words, we normalized the median light-up value with respect to the background. This metric is referred to as the TF-target covariance-fold in the manuscript.

### The backwards-selection algorithm

We used a backwards-selection algorithm to identify the core set of TFs, i.e., the minimal set of TFs that could predict the target genes. The algorithm operated according to the following three steps. First, each input node, corresponding to one TF, was independently set to zero, and the corresponding 1 - *R*^2^ values were calculated. Second, the 100 TFs with the lowest explanatory power, identified as the ones where the 1 - *R*^2^ changed the least, were removed from the input layer of the DNN. Thirdly, we retrained the new and smaller model to explain the rest of the gene expressions. For each iteration, we tested the model on the same test data from the ARCHS4-database as in the first validation experiment.

### Disease analyses

To analyze diseases, we searched the Expression Atlas^33^ to download all data according to the following criteria. I) The data was to come from a study carried out in human material. II) We only considered RNA-seq experiments. III) The data sets had the term ‘disease’ listed as an experimental variable and be of the ‘differential’ type. This query yielded data from a total of 27 studies, which together contained 69 expression fold profiles. Of these 69 comparisons, 56 were between a disease-affected and a healthy state, as opposed to between two disease-states, and we continued with these 56 studies. The 56 studies contain fold-changes between the healthy and disease-affected states, and by adding these changes to the mean expression values from the ARCHS4 database we could predict the fold changes on the target gene level. Furthermore, we chose only to study the correlation between the genes that were differentially expressed at a false discovery rate of 0.05.

The predictions of TFs causative of disease were made by adding the fold changes to the TFs as described above, followed by removing the dysregulation of each TF independently. This resulted in 1625 predicted changes in correlation between predicted and measured target expressions as compared to that from the full TF profile. We ranked the TFs on change in correlations and calculated the area under the receiver operating characteristics curve, i.e., the AUROC, for this ranking. A true positive identification was defined as the TF being associated with the respective disease, as manually curated from the GWAS catalog^31^. We next Monte Carlo sampled 10,000 random TF permutations and estimated the *P* value from the random AUROC distribution.

## Supplementary information

Supplemental Material 1-3

Table 2

Table 3

Table 1

## Data Availability

The training data were downloaded from the ARCHS4 database, annotated as “gene level” (accessed at https://maayanlab.cloud/archs4/download.html) and used to train the models. The curated disease data files can be accessed at https://github.com/rasma774/tf_target_white_box_dl.

## Code availability

The code used to generate the results, along with the DNN with 250 hidden nodes in two layers, can be found at https://github.com/rasma774/tf_target_white_box_dl.
