# Supplementary material for: Deep neural network prediction of genome-wide transcriptome signatures – beyond the Black-box
Source: NPJ Syst Biol Appl. 2022 Feb 23;8:9. doi: 10.1038/s41540-022-00218-9 (PMC8866467; doi:10.1038/s41540-022-00218-9)
Supplement: Supplementary file 1 — Supplemental Material 1-3 [file 41540_2022_218_MOESM1_ESM.pdf]

Supplementary material 1: Analysis of prediction power on individual human tissues in GTEx data

To assert how well the gene regulatory patterns of different human tissues were learnt during the training on the ARCHS4 data, we used the RNA-Seq data of the GTEx resource, V8 (downloaded the 8:th of November 2021). We extracted gene counts data corresponding to 54 different human tissues, and separated the gene counts into matrices representing the same TFs and target genes as in the training data. The data were log-transformed to follow the same procedure as in the data preprocessing of the ARCHS4 data.

Next, we used the the deep neural network of two hidden layers with 250 nodes each, together with the gene expression of the TFs, to predict the gene expression of the target genes. We used four measures to assess the performance of each tissue: the median and mean of the R2 coefficients respective to each target gene, and the median and mean of the Spearman correlation of each target gene (Supplementary table 1). We found the model to perform well on next-to-all tested human tissues, with the prediction performance on the tissue “Testis” having the worst performance (median R2 = 0.446). The GTEx samples that were annotated as “Cells_Cultured_Fibroblasts” were associated with the highest performance in the model predictions, which we speculate to resonate well with the relatively high proportion of cell line-derived material in the ARCHS4 training data.

Supplementary table 1: Performance on individual tissues in the GTEx data.

	Median R2	Mean R2	Median Spearman Rho	Mean Spearman Rho
Testis	0.446	0.452	0.762	0.765
Pituitary	0.666	0.666	0.829	0.829
Spleen	0.675	0.675	0.827	0.827
Small_Intestine_Terminal_Ileum	0.677	0.680	0.832	0.833
Minor_Salivary_Gland	0.678	0.676	0.829	0.829
Kidney_Cortex	0.688	0.687	0.831	0.831
Prostate	0.688	0.688	0.836	0.836
Kidney_Medulla	0.688	0.690	0.833	0.834
Colon_Transverse	0.691	0.692	0.834	0.835
Brain_Cerebellum	0.693	0.692	0.834	0.834
Brain_Hypothalamus	0.694	0.696	0.837	0.837
Skin_Not_Sun_Exposed_(Suprapubic)	0.695	0.693	0.833	0.833
Skin_Sun_Exposed_(Lower_leg)	0.695	0.694	0.833	0.833
Brain_Cerebellar_Hemisphere	0.696	0.696	0.834	0.834
Thyroid	0.696	0.695	0.836	0.836
Esophagus_Mucosa	0.697	0.696	0.832	0.832
Pancreas	0.697	0.697	0.833	0.833
Vagina	0.697	0.696	0.835	0.835
Brain_Nucleus_accumbens_(basal_ganglia)	0.697	0.701	0.838	0.837
Stomach	0.697	0.699	0.835	0.836
Lung	0.698	0.697	0.841	0.841
Fallopian_Tube	0.700	0.697	0.839	0.837
Ovary	0.702	0.701	0.834	0.834
Brain_Caudate_(basal_ganglia)	0.703	0.703	0.839	0.839

Cervix_Ectocervix	0.703	0.702	0.838	0.836
Nerve_Tibial	0.705	0.705	0.837	0.837
Liver	0.706	0.706	0.832	0.832
Brain_Spinal_cord_(cervical_c-1)	0.707	0.708	0.839	0.839
Cervix_Endocervix	0.708	0.703	0.840	0.839
Adrenal_Gland	0.708	0.708	0.836	0.836
Brain_Cortex	0.708	0.707	0.843	0.843
Brain_Putamen_(basal_ganglia)	0.709	0.712	0.840	0.840
Brain_Hippocampus	0.710	0.710	0.842	0.842
Brain_Substantia_nigra	0.711	0.711	0.841	0.841
Breast_Mammary_Tissue	0.711	0.711	0.842	0.842
Brain_Frontal_Cortex_(BA9)	0.713	0.713	0.844	0.843
Brain_Amygdala	0.714	0.715	0.843	0.843
Brain_Anterior_cingulate_cortex_(BA24)	0.714	0.713	0.844	0.843
Uterus	0.715	0.714	0.839	0.839
Bladder	0.716	0.712	0.842	0.840
Colon_Sigmoid	0.716	0.714	0.842	0.841
Adipose_Visceral_(Omentum)	0.721	0.720	0.843	0.843
Heart_Atrial_Appendage	0.722	0.722	0.842	0.842
Esophagus_Gastroesophageal_Junction	0.724	0.723	0.844	0.844
Artery_Aorta	0.725	0.724	0.841	0.841
Artery_Coronary	0.725	0.723	0.843	0.843
Esophagus_Muscularis	0.725	0.723	0.844	0.843
Adipose_Subcutaneous	0.731	0.730	0.845	0.845
Artery_Tibial	0.732	0.731	0.842	0.841
Cells_EBV-transformed_lymphocytes	0.732	0.733	0.829	0.829
Whole_Blood	0.734	0.734	0.829	0.829
Heart_Left_Ventricle	0.737	0.738	0.844	0.844
Muscle_Skeletal	0.749	0.748	0.844	0.844
Cells_Cultured_fibroblasts	0.764	0.763	0.845	0.845

Supplementary material 2:

DoRothEA results :

TFs_to_targets_250_250_250.csv
AUC: 0.6750707998568443, AP: 0.9556066922259717
n corr: 2897, n tot: 4498

TFs_to_targets_250.csv
AUC: 0.7337516921943805, AP: 0.965824645784112
n corr: 3006, n tot: 4498

TFs_to_targets_100_100_100.csv
AUC: 0.684311737215828, AP: 0.9547763076679238
n corr: 2870, n tot: 4498

TFs_to_targets_1000.csv
AUC: 0.6742927794519624, AP: 0.9566777403658694
n corr: 2905, n tot: 4498

TFs_to_targets_spearmanr.csv
AUC: 0.9698426324061059, AP: 0.9969860959534833
n corr: 3829, n tot: 4498

TFs_to_targets_100.csv
AUC: 0.6937173555605118, AP: 0.9580971287933397
n corr: 2934, n tot: 4498

TFs_to_targets_1000_1000_1000.csv
AUC: 0.6757755566735997, AP: 0.9554574210114752
n corr: 2900, n tot: 4498

TFs_to_targets_500_500_500.csv
AUC: 0.6737760442330534, AP: 0.9560350975080136
n corr: 2888, n tot: 4498

TFs_to_targets_50.csv
AUC: 0.7236296467268681, AP: 0.9607887242081521
n corr: 3021, n tot: 4498

TFs_to_targets_no_hidden_.csv
AUC: 0.6645383486257566, AP: 0.9534499393091929
n corr: 2554, n tot: 4498

TFs_to_targets_50_50_50.csv
AUC: 0.7002903312810884, AP: 0.9584955781749502
n corr: 2978, n tot: 4498

TFs_to_targets_50_50.csv
AUC: 0.7022697448611752, AP: 0.9572874013020993
n corr: 3020, n tot: 4498

TFs_to_targets_500_500.csv

AUC: 0.6295617929739571, AP: 0.9489232635411091
n corr: 2784, n tot: 4498

TFs_to_targets_250_250.csv
AUC: 0.7429576186351448, AP: 0.9675751600306878
n corr: 3021, n tot: 4498

TFs_to_targets_1000_1000.csv
AUC: 0.7006041328443907, AP: 0.9594967900793749
n corr: 2863, n tot: 4498

TFs_to_targets_500.csv
AUC: 0.6755421505521351, AP: 0.9561587324828115
n corr: 2914, n tot: 4498

TFs_to_targets_100_100.csv
AUC: 0.682758289807414, AP: 0.9570792843846412
n corr: 2911, n tot: 4498

TRRUST results:

Tfs_to_targets_250_250_250.csv
AUC: 0.670909527646913, AP: 0.8138270082804918
n corr: 1957, n tot: 3458

Tfs_to_targets_250.csv
AUC: 0.7020704190428961, AP: 0.8283017951663989
n corr: 1985, n tot: 3458

Tfs_to_targets_100_100_100.csv
AUC: 0.6397811802628317, AP: 0.7790028662376707
n corr: 1933, n tot: 3458

Tfs_to_targets_1000.csv
AUC: 0.6563088891643938, AP: 0.8020568284016005
n corr: 1992, n tot: 3458

Tfs_to_targets_spearmanr.csv
AUC: 0.9590309633027523, AP: 0.9797966207211075
n corr: 2264, n tot: 3458

Tfs_to_targets_100.csv
AUC: 0.675046878874287, AP: 0.8089876718633584
n corr: 1975, n tot: 3458

Tfs_to_targets_1000_1000_1000.csv
AUC: 0.6719501611703447, AP: 0.8103590442160198
n corr: 1984, n tot: 3458

Tfs_to_targets_500_500_500.csv
AUC: 0.6494599243739152, AP: 0.7954856257804359
n corr: 1952, n tot: 3458

Tfs_to_targets_50.csv
AUC: 0.6797545251673691, AP: 0.8098362162795755
n corr: 1996, n tot: 3458

Tfs_to_targets_no_hidden_.csv
AUC: 0.6451832925241755, AP: 0.7953751419763172
n corr: 1733, n tot: 3458

Tfs_to_targets_50_50_50.csv
AUC: 0.6647959025539301, AP: 0.8008448329704512
n corr: 1921, n tot: 3458

Tfs_to_targets_50_50.csv
AUC: 0.6770405870319861, AP: 0.8034297243066475
n corr: 1998, n tot: 3458

Tfs_to_targets_500_500.csv
AUC: 0.6431549094966527, AP: 0.792028706538777
n corr: 1965, n tot: 3458

Tfs_to_targets_250_250.csv
AUC: 0.70053077733697, AP: 0.8284202773227727
n corr: 2013, n tot: 3458

Tfs_to_targets_1000_1000.csv
AUC: 0.664138823456484, AP: 0.802734455107331
n corr: 1944, n tot: 3458

Tfs_to_targets_500.csv
AUC: 0.6648799745846765, AP: 0.8044161147570315
n corr: 1974, n tot: 3458

Tfs_to_targets_100_100.csv
AUC: 0.6615469253657327, AP: 0.8004666784457402
n corr: 1940, n tot: 3458

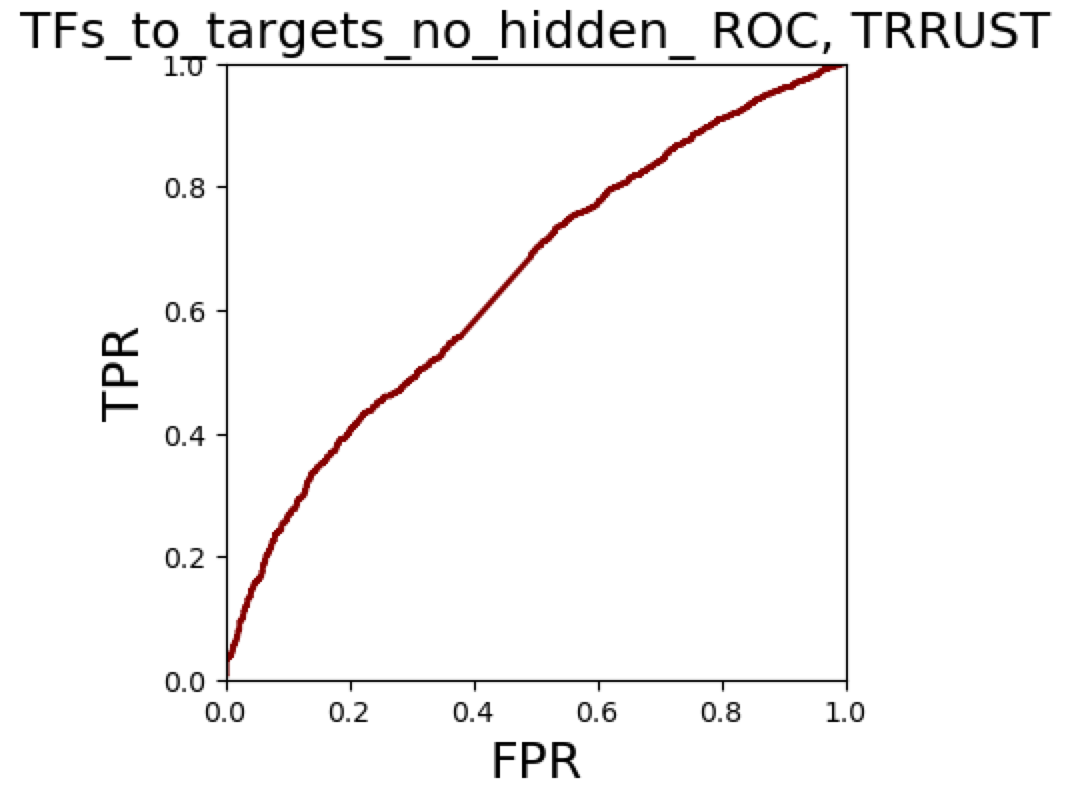
TFs_to_targets_no_hidden_ ROC, TRRUST
TPR
FPR
0.0
0.2
0.4
0.6
0.8
1.0

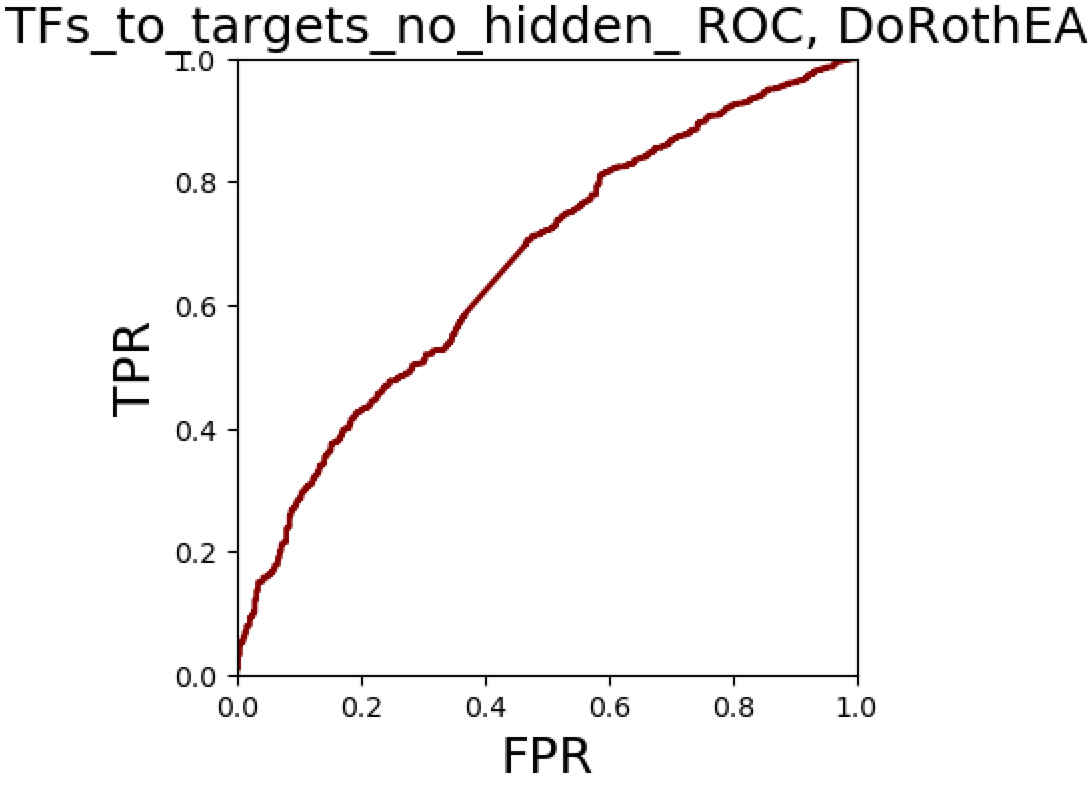

TFs_to_targets_no_hidden_ ROC, DoRothEA
TPR
FPR
0.0
0.2
0.4
0.6
0.8
1.0
0.0
0.2
0.4
0.6
0.8
1.0

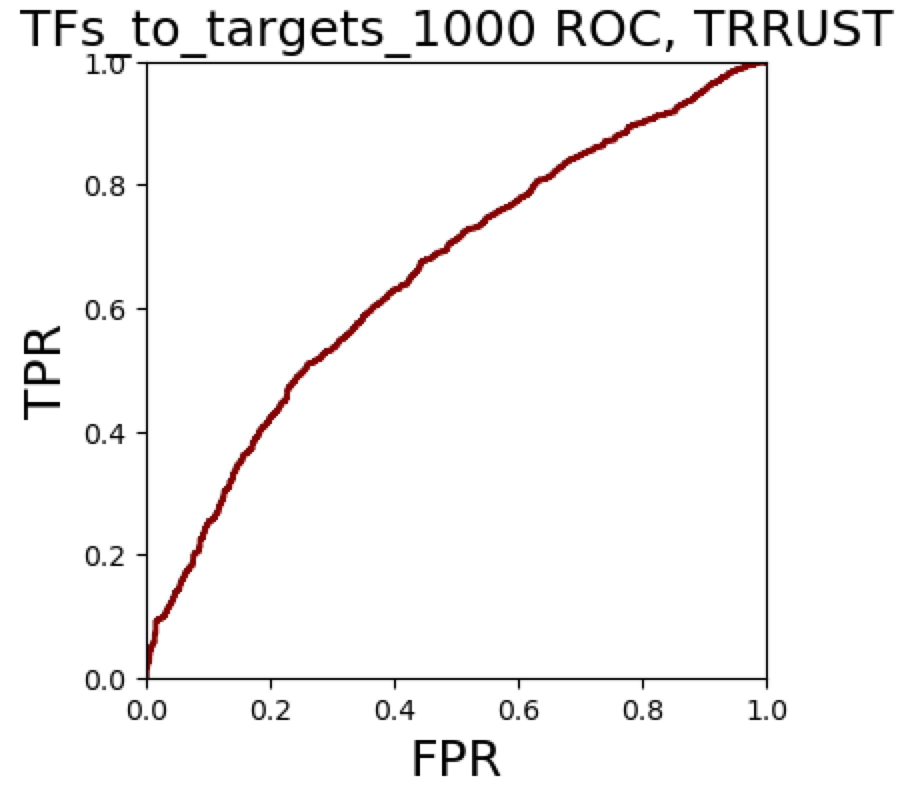

TFs_to_targets_1000 ROC, TRRUST
TPR
FPR
0.0
0.2
0.4
0.6
0.8
1.0

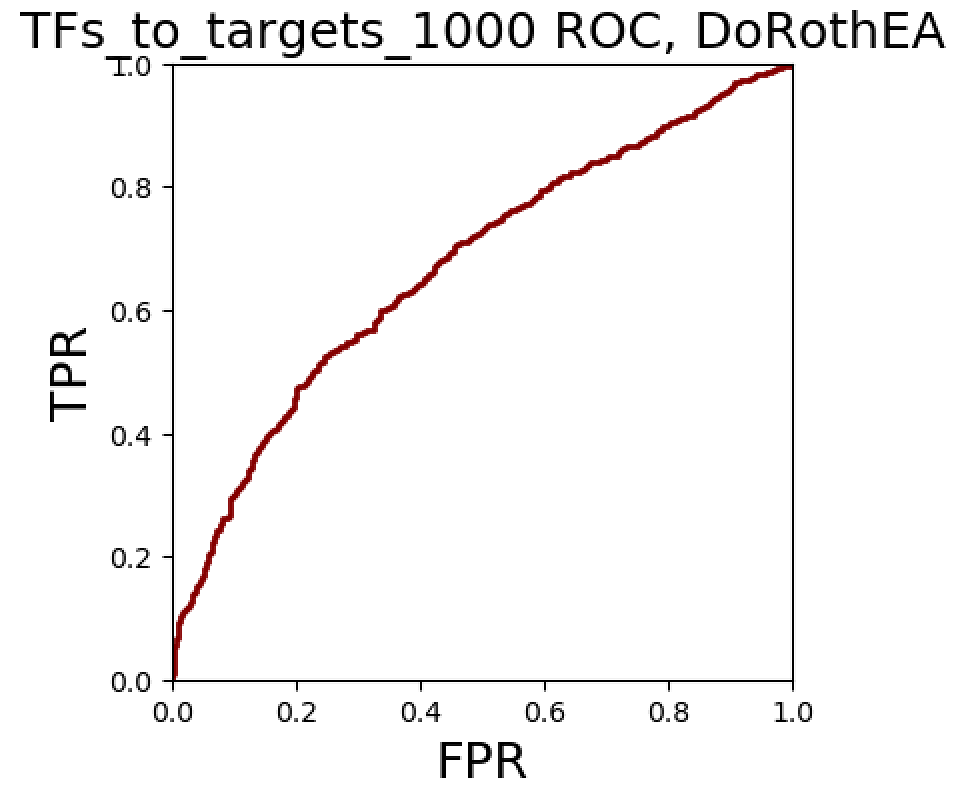
TFs_to_targets_1000 ROC, DoRothEA
TPR
FPR
0.0
0.2
0.4
0.6
0.8
1.0

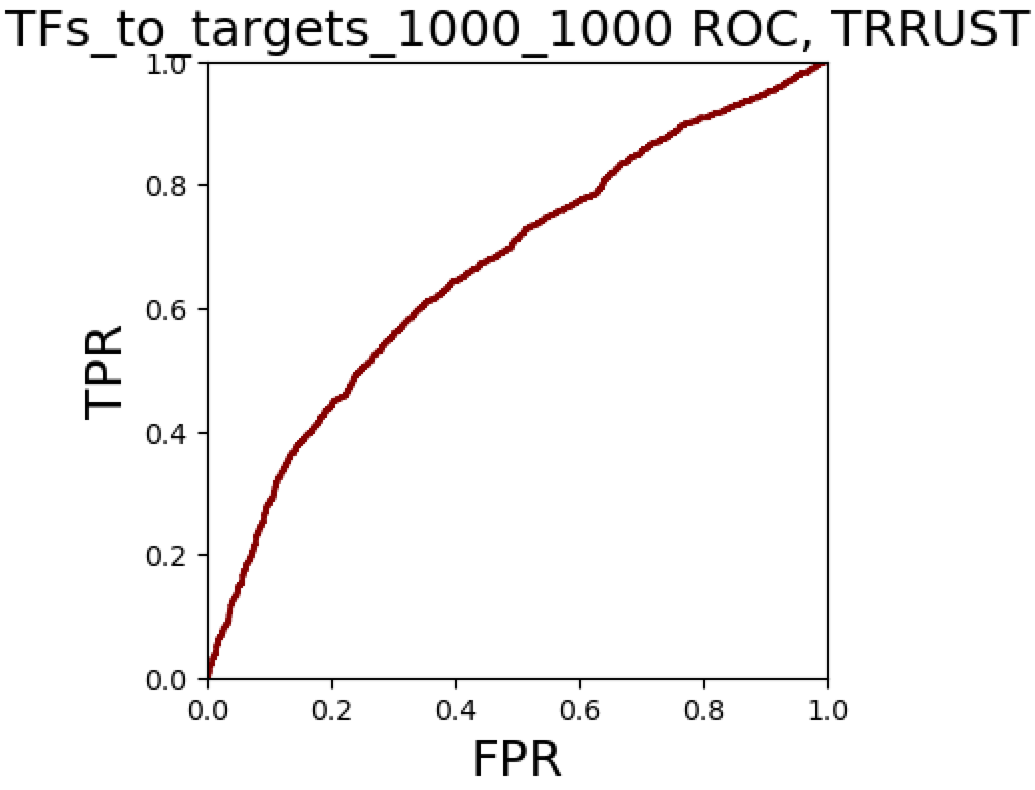
TFs_to_targets_1000_1000 ROC, TRRUST
TPR
FPR
0.0
0.2
0.4
0.6
0.8
1.0
0.0
0.2
0.4
0.6
0.8
1.0

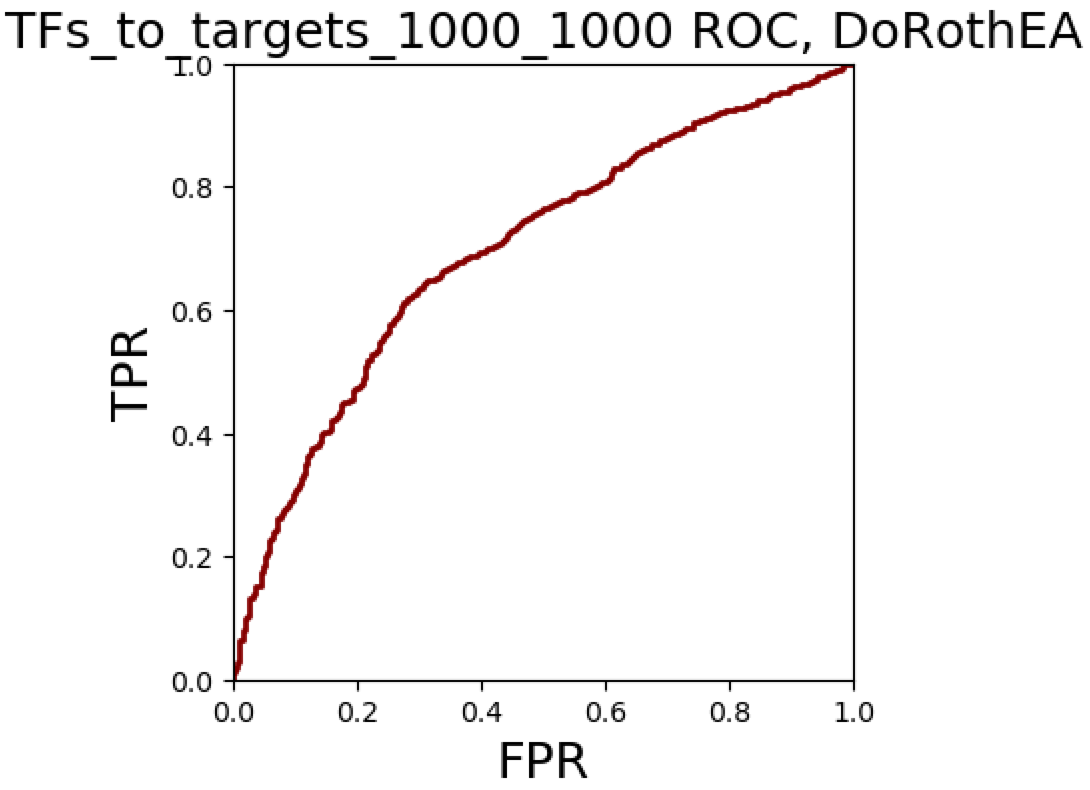

TFs_to_targets_1000_1000 ROC, DoRothEA
TPR
FPR
0.0
0.2
0.4
0.6
0.8
1.0

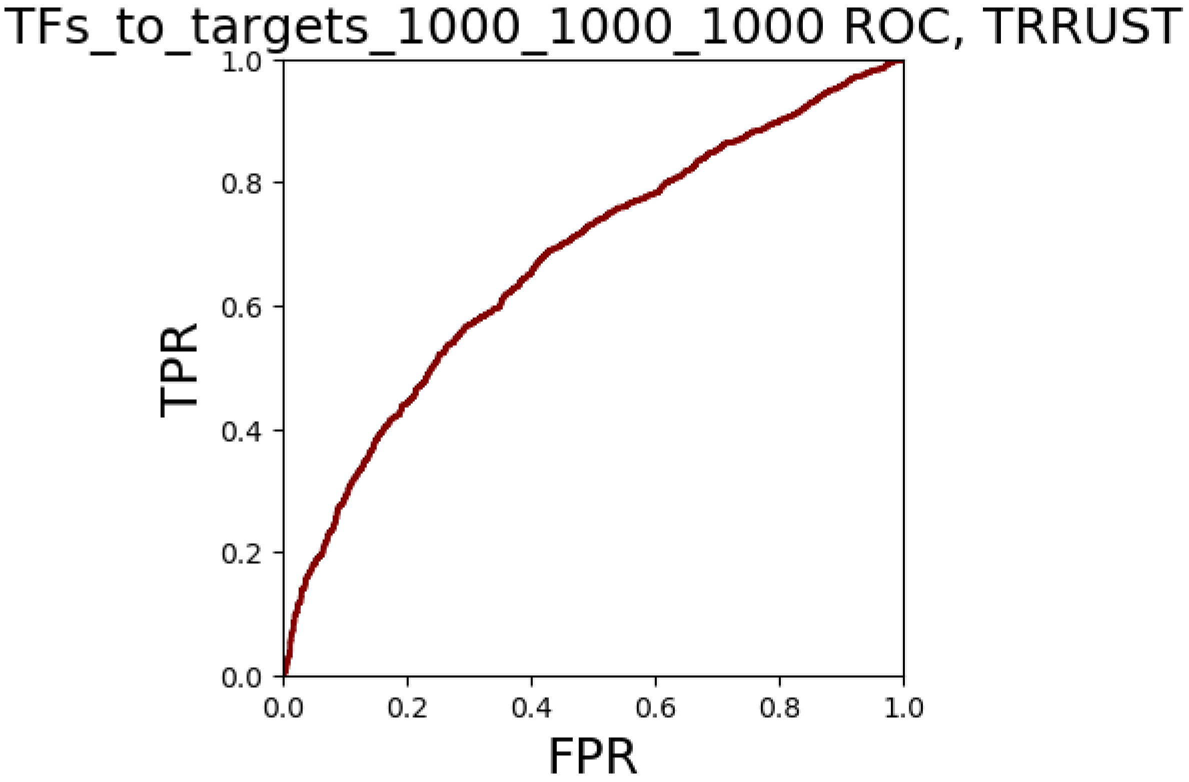
TFs_to_targets_1000_1000_1000 ROC, TRRUST
TPR
FPR
0.0
0.2
0.4
0.6
0.8
1.0

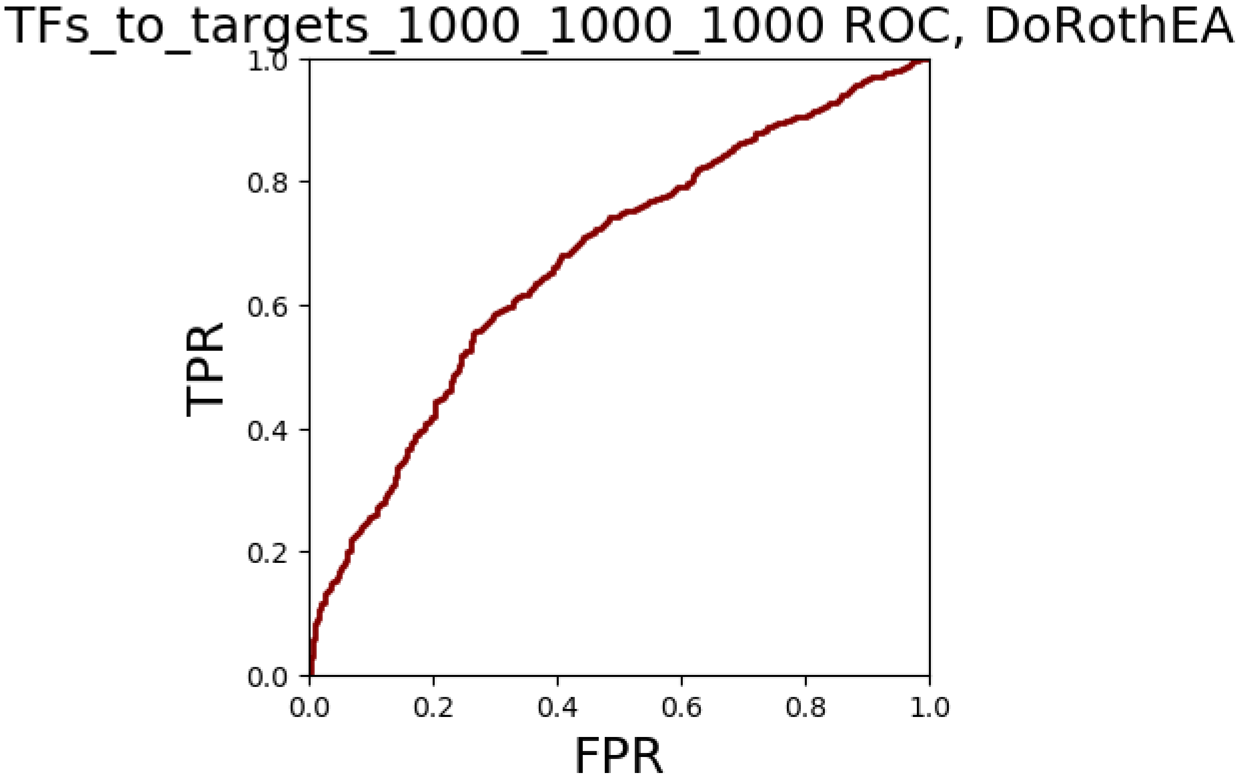
TFs_to_targets_1000_1000_1000 ROC, DoRothEA
TPR
FPR
0.0
0.2
0.4
0.6
0.8
1.0

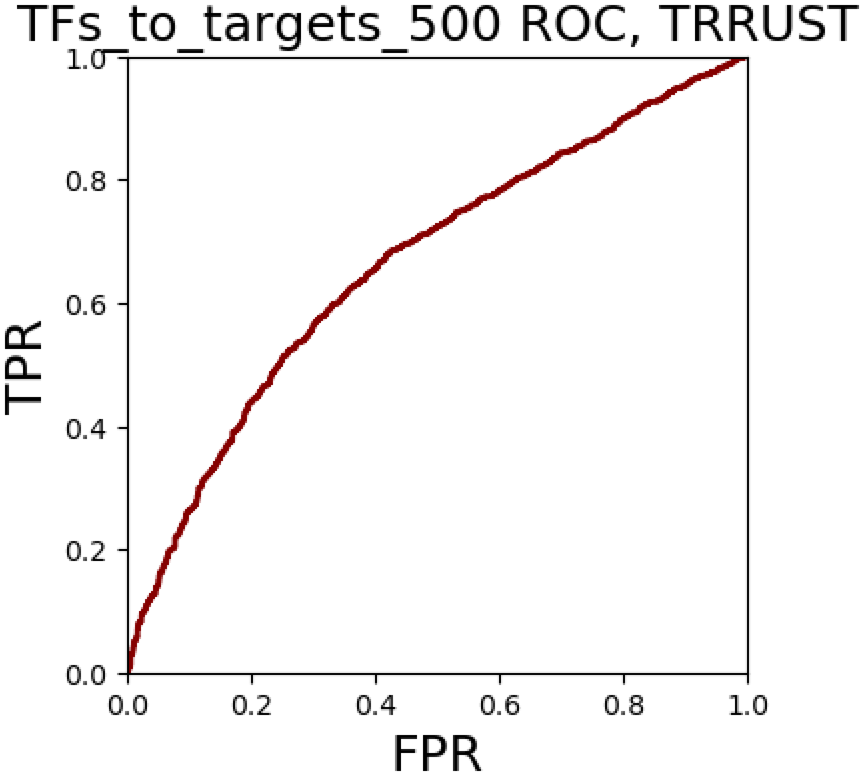

TFs_to_targets_500 ROC, TRRUST
TPR
FPR
0.0
0.2
0.4
0.6
0.8
1.0

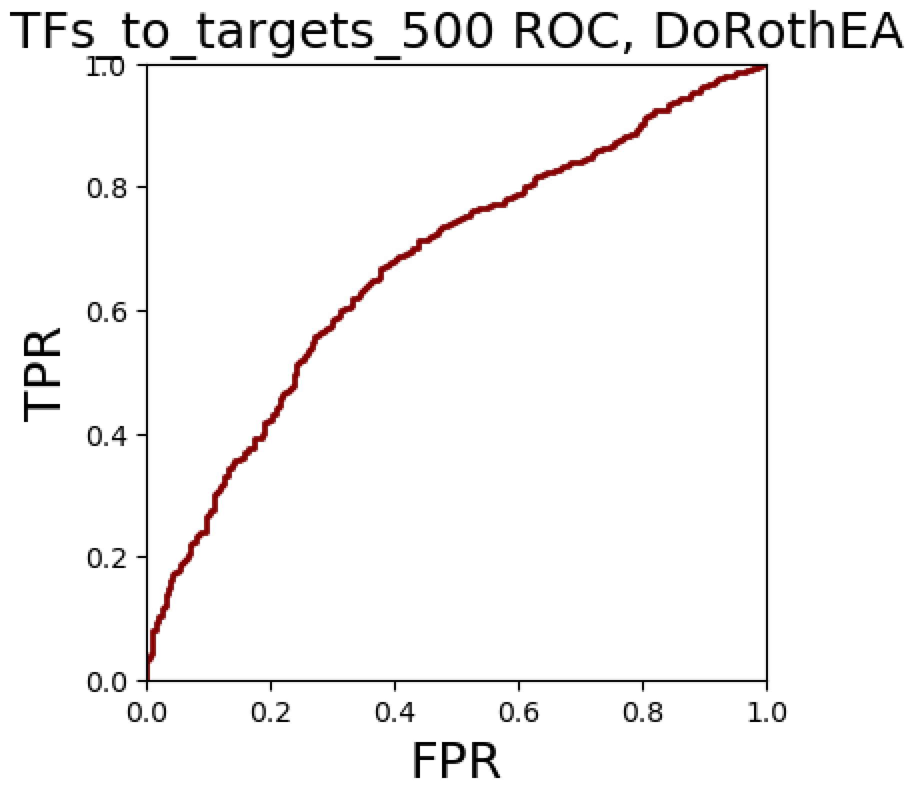

TFs_to_targets_500 ROC, DoRothEA
TPR
FPR
0.0
0.2
0.4
0.6
0.8
1.0

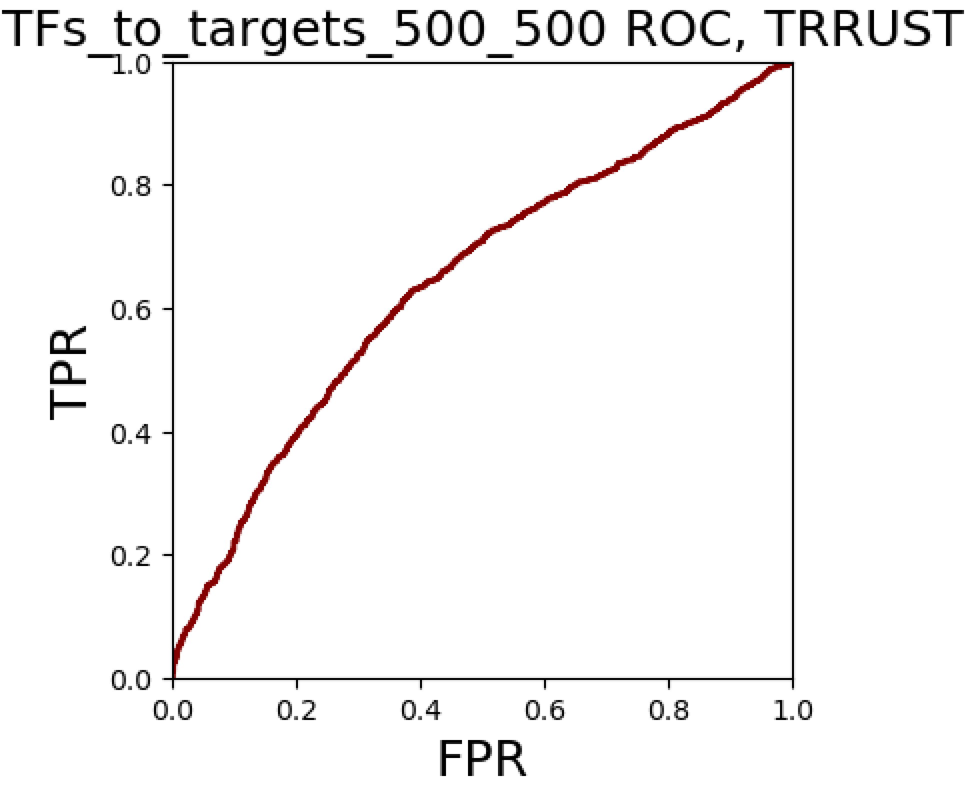
TFs_to_targets_500_500 ROC, TRRUST
TPR
FPR
0.0
0.2
0.4
0.6
0.8
1.0

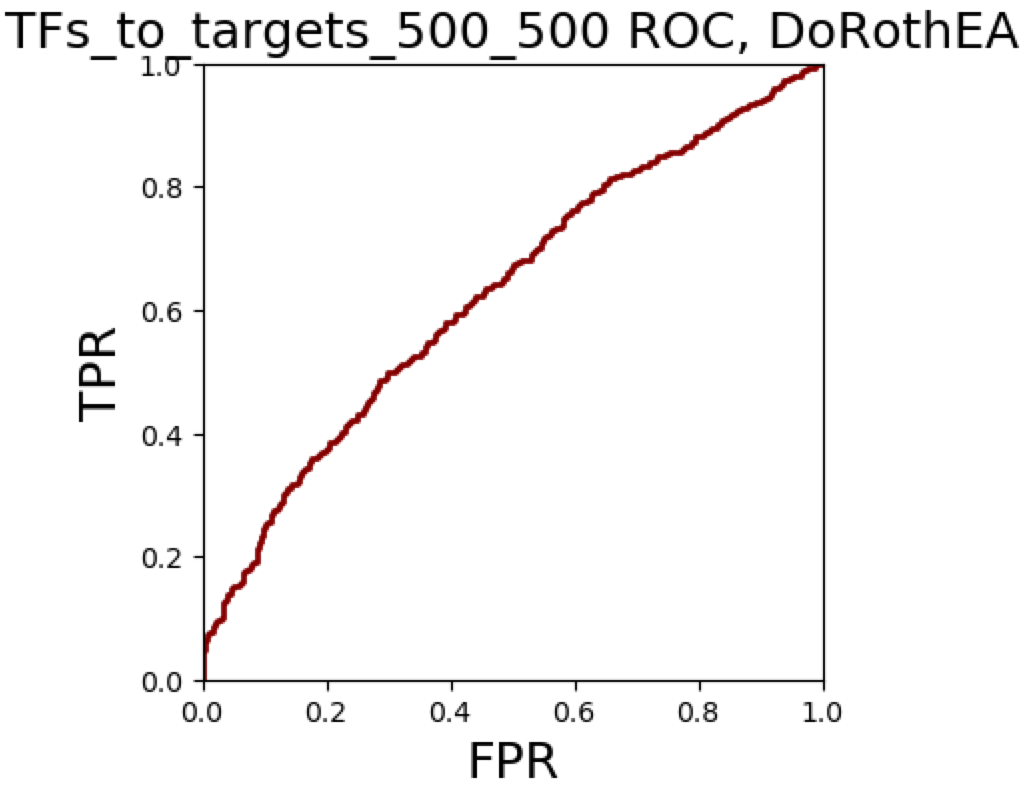

TFs_to_targets_500_500 ROC, DoRothEA
TPR
FPR
0.0
0.2
0.4
0.6
0.8
1.0

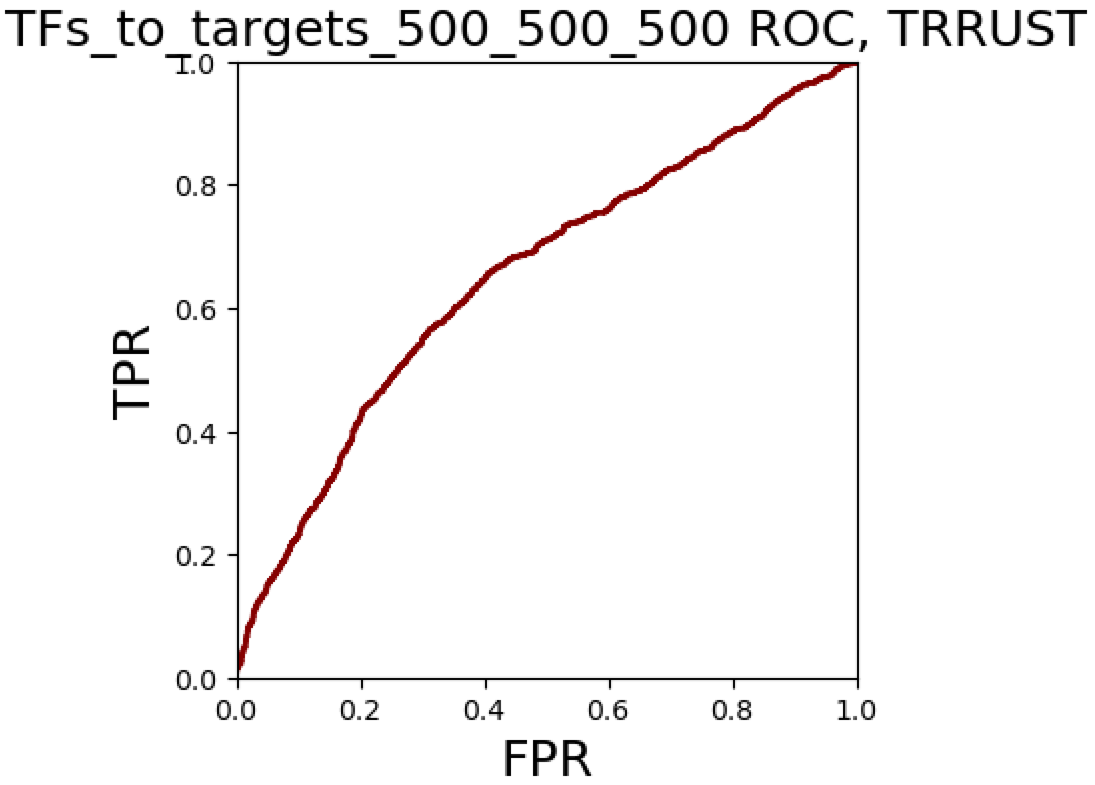
TFs_to_targets_500_500_500 ROC, TRRUST
TPR
FPR
0.0
0.2
0.4
0.6
0.8
1.0
0.0
0.2
0.4
0.6
0.8
1.0

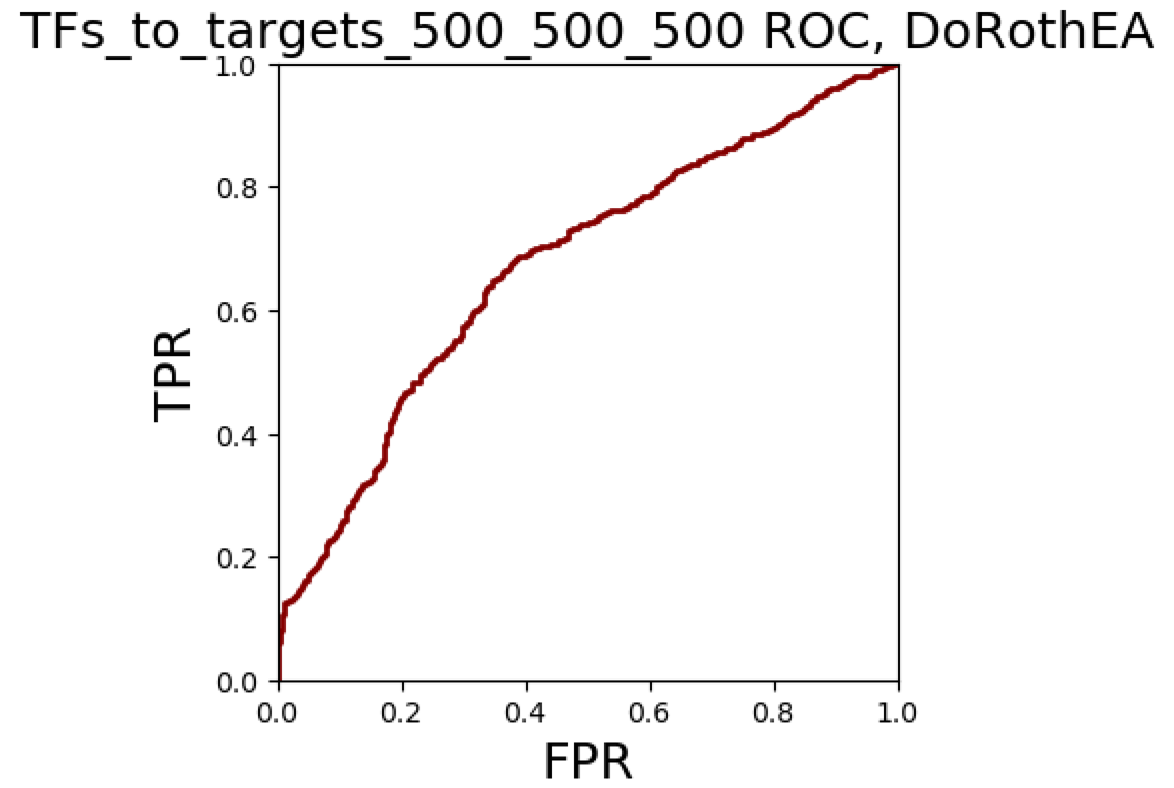

TFs_to_targets_500_500_500 ROC, DoRothEA
TPR
FPR
0.0
0.2
0.4
0.6
0.8
1.0

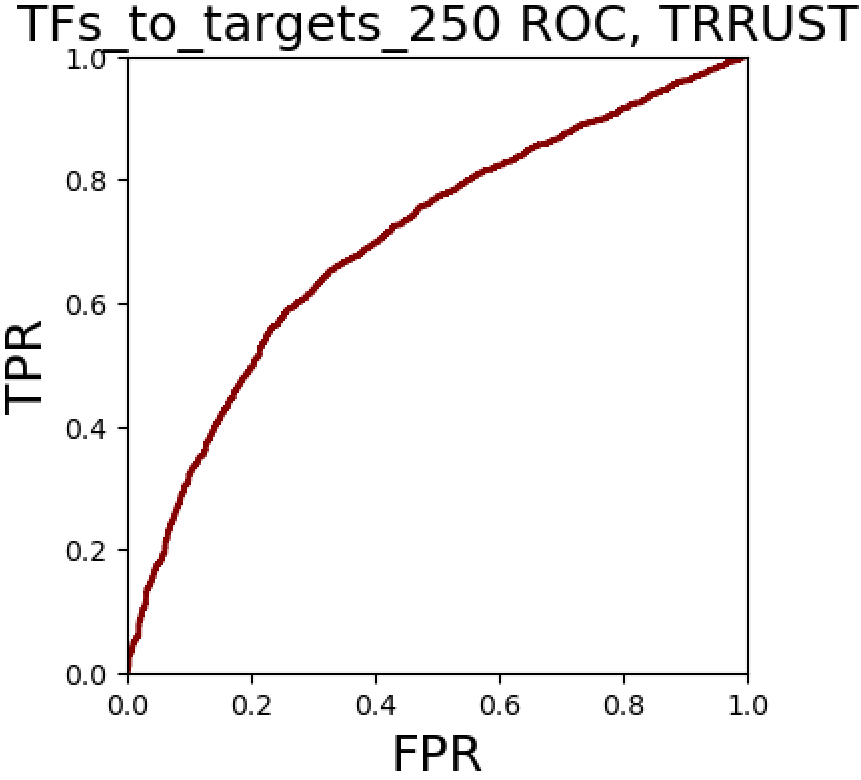

TFs_to_targets_250 ROC, TRRUST
TPR
FPR
0.0
0.2
0.4
0.6
0.8
1.0

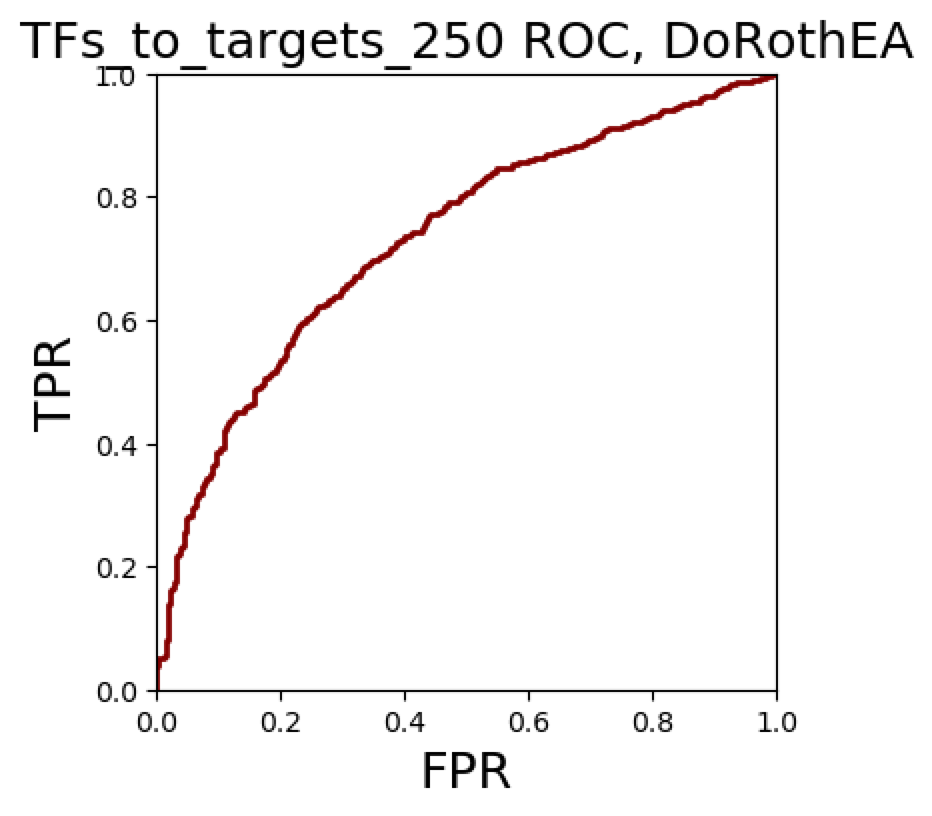

TFs_to_targets_250 ROC, DoRothEA
TPR
FPR
0.0
0.2
0.4
0.6
0.8
1.0

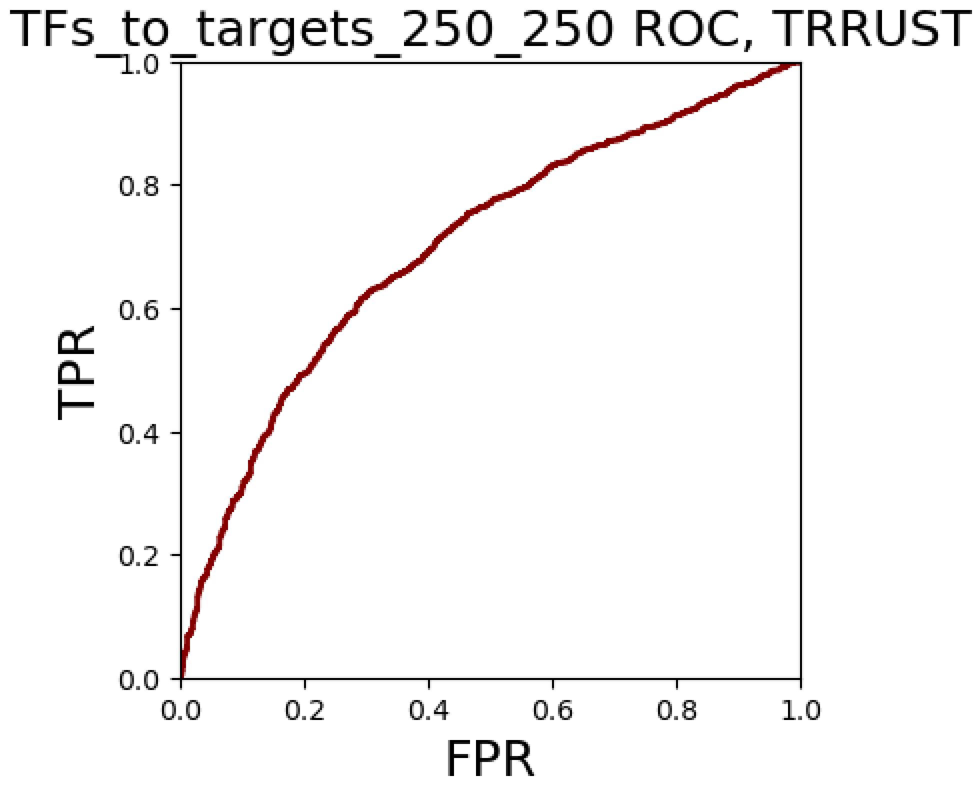
TFs_to_targets_250_250 ROC, TRRUST
TPR
FPR
0.0
0.2
0.4
0.6
0.8
1.0

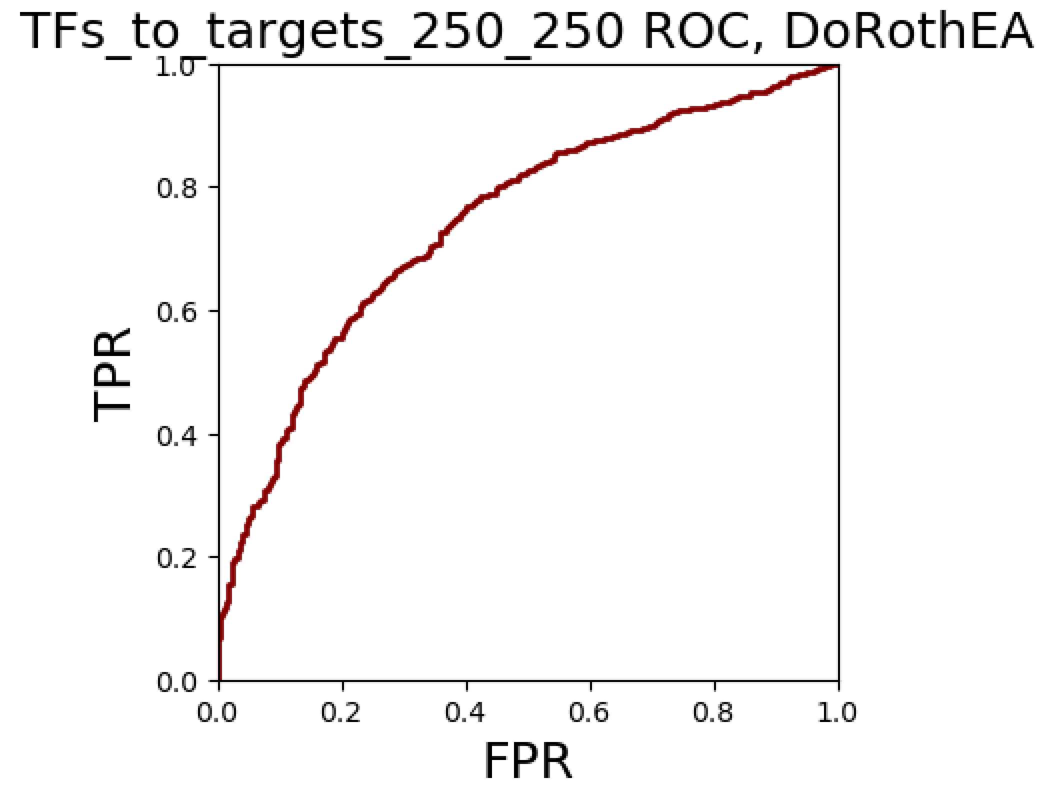
TFs_to_targets_250_250 ROC, DoRothEA
TPR
FPR
0.0
0.2
0.4
0.6
0.8
1.0

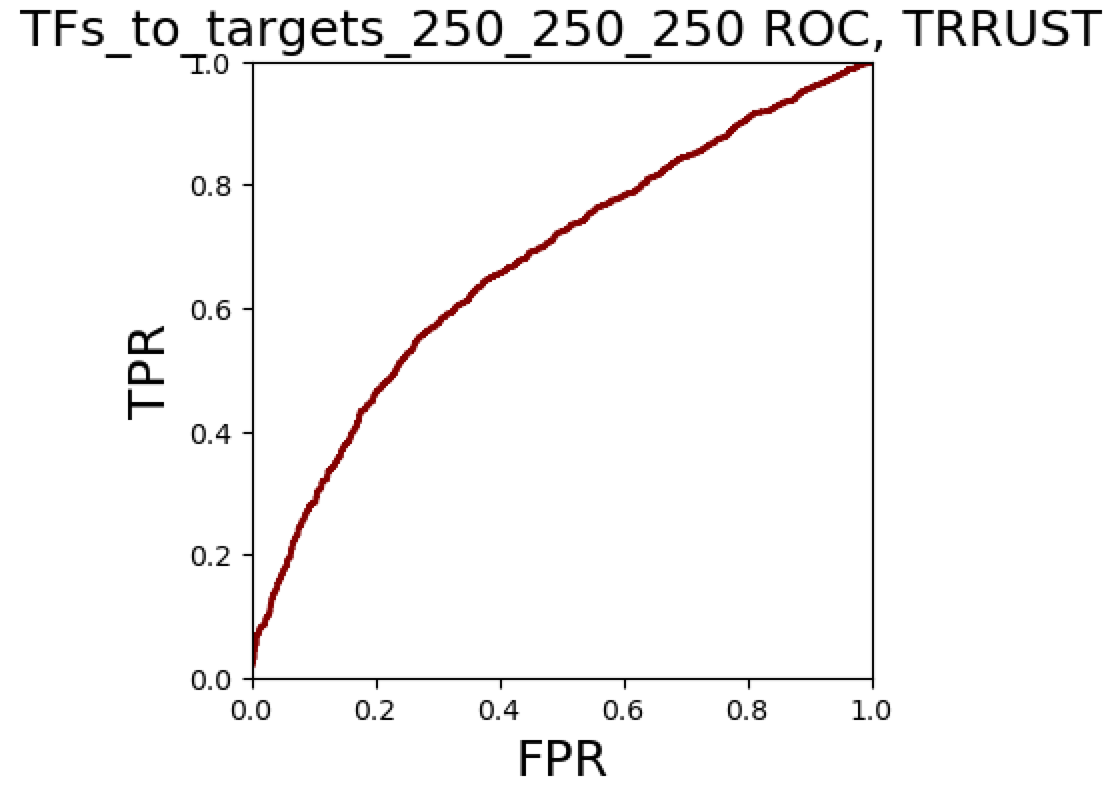

TFs_to_targets_250_250_250 ROC, TRRUST
TPR
FPR
0.0
0.2
0.4
0.6
0.8
1.0

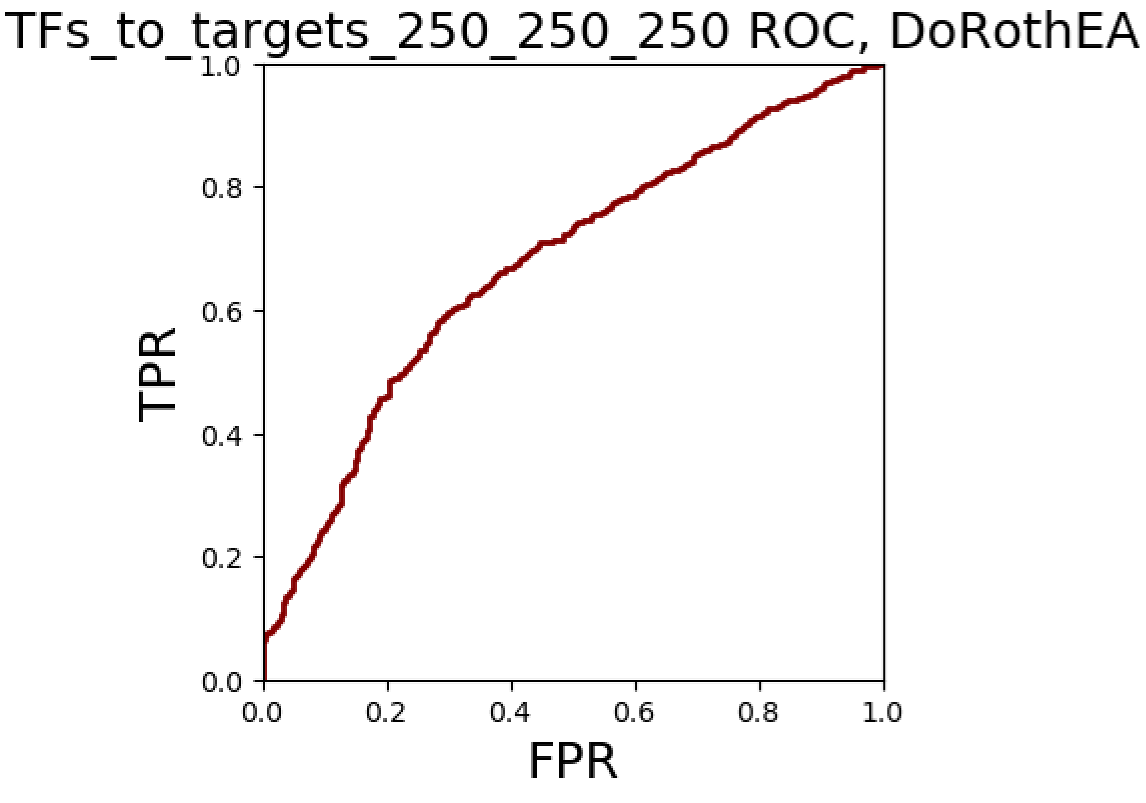

TFs_to_targets_250_250_250 ROC, DoRothEA
TPR
FPR
0.0
0.2
0.4
0.6
0.8
1.0

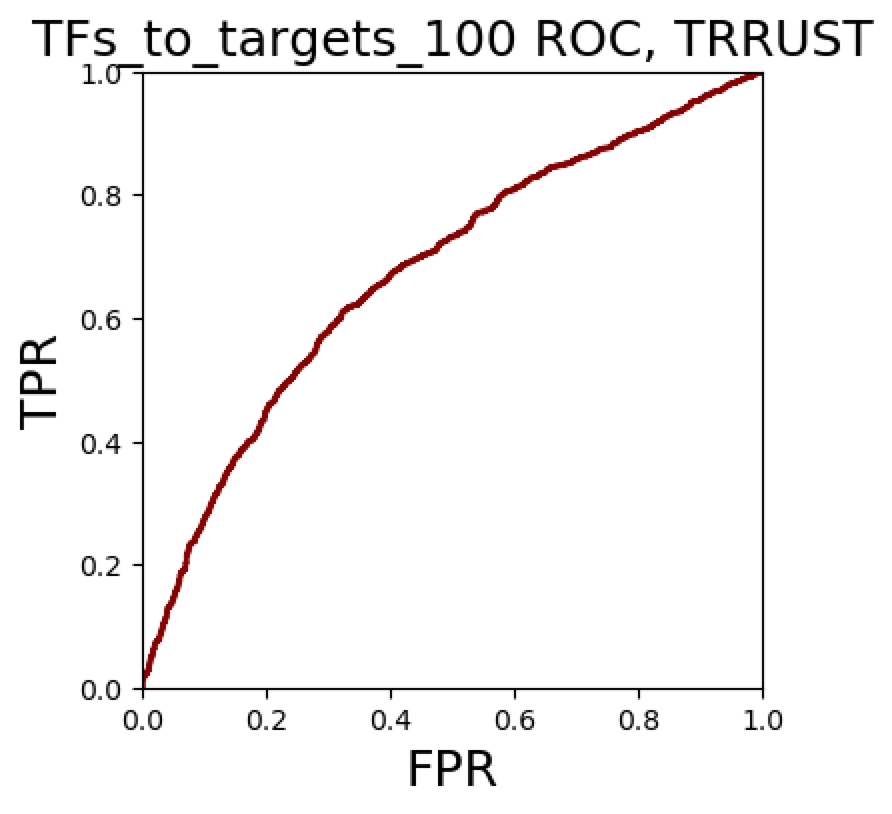
TFs_to_targets_100 ROC, TRRUST
TPR
FPR
0.0
0.2
0.4
0.6
0.8
1.0

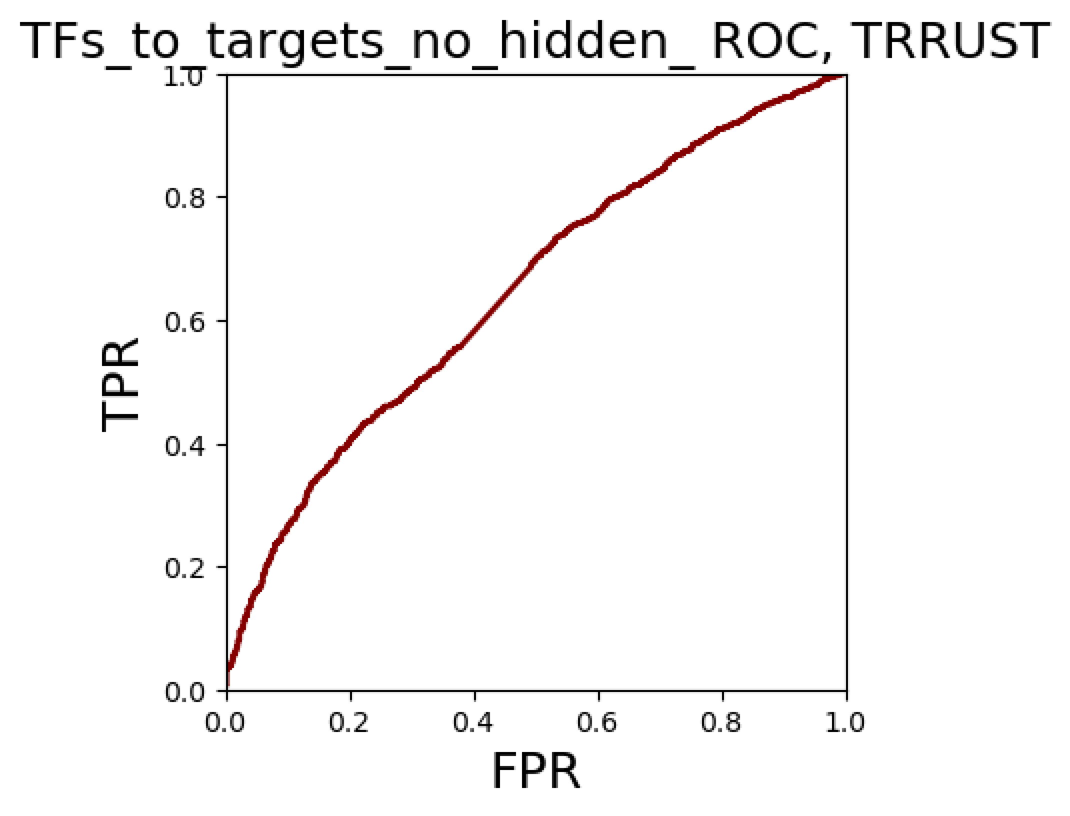

TFs_to_targets_no_hidden_ ROC, TRRUST
TPR
FPR
0.0
0.2
0.4
0.6
0.8
1.0
0.0
0.2
0.4
0.6
0.8
1.0

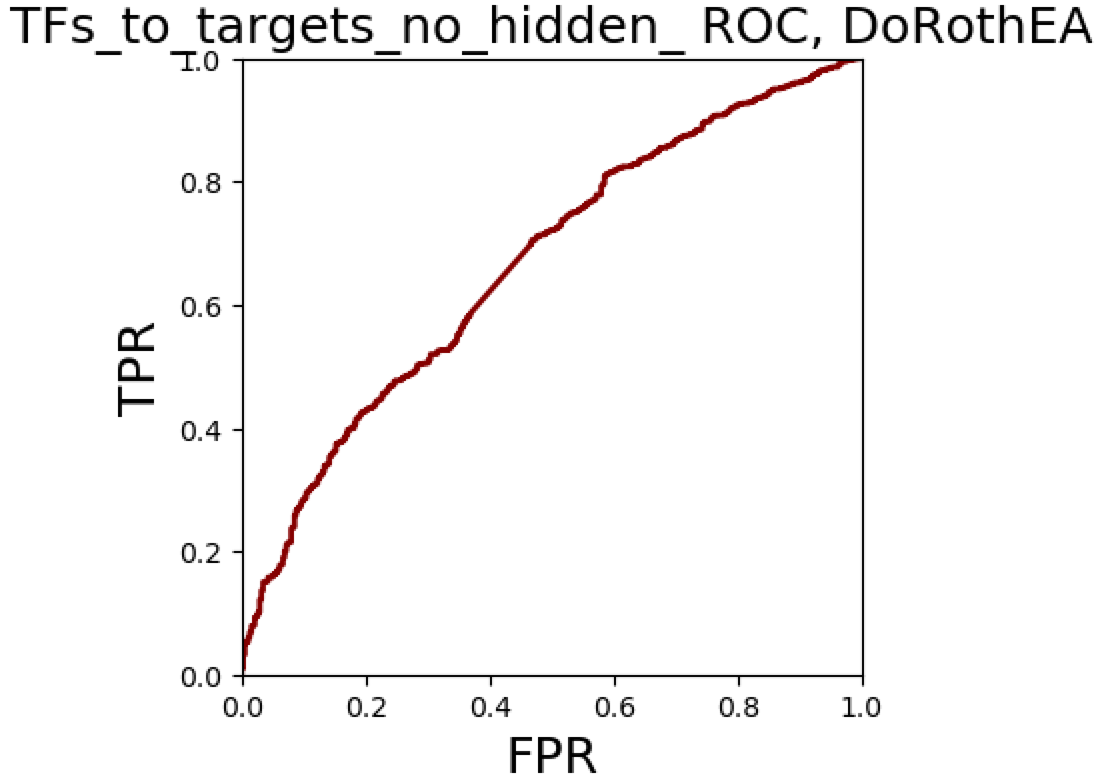

TFs_to_targets_no_hidden_ ROC, DoRothEA
1.0
0.8
0.6
0.4
0.2
0.0
TPR
0.0
0.2
0.4
0.6
0.8
1.0
FPR

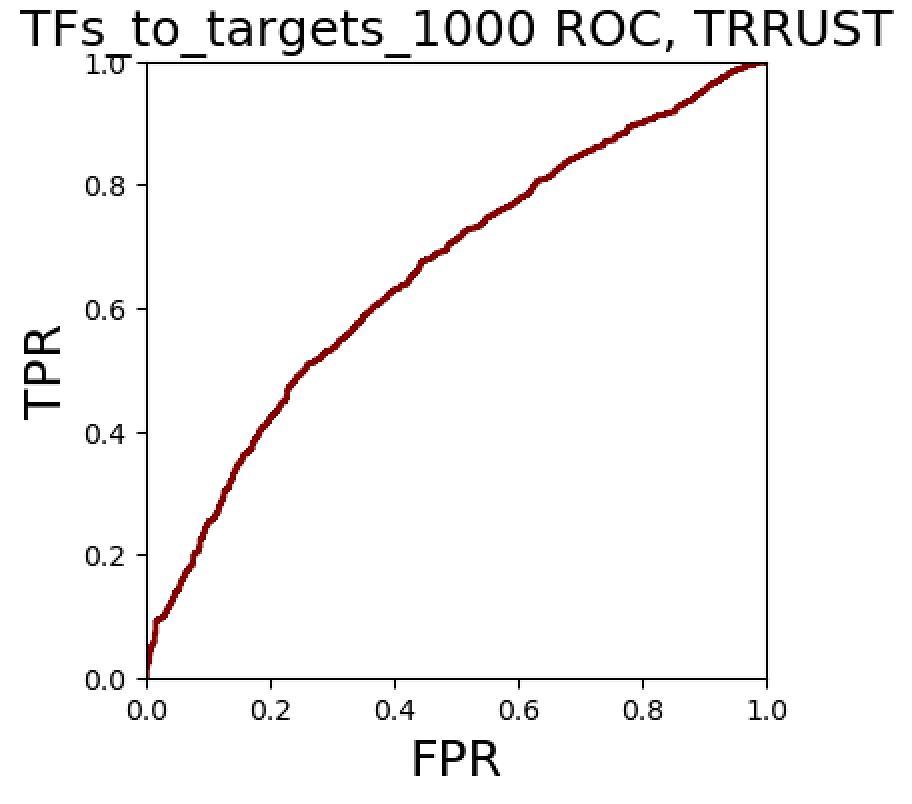
TFs_to_targets_1000 ROC, TRRUST
TPR
FPR
0.0
0.2
0.4
0.6
0.8
1.0

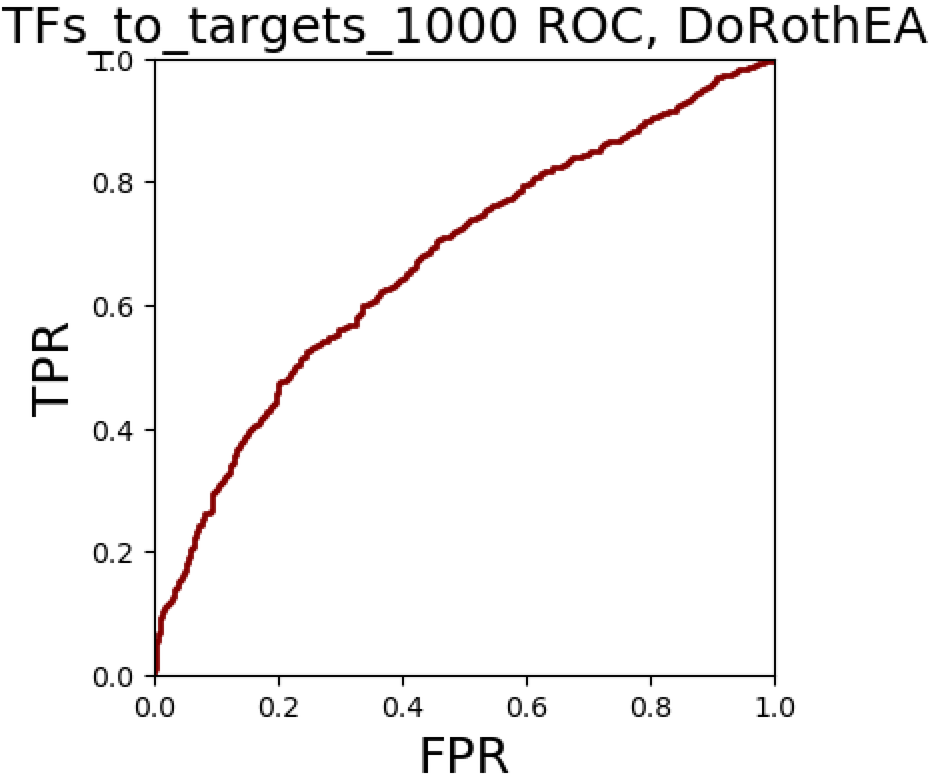

TFs_to_targets_1000 ROC, DoRothEA
TPR
FPR
0.0
0.2
0.4
0.6
0.8
1.0

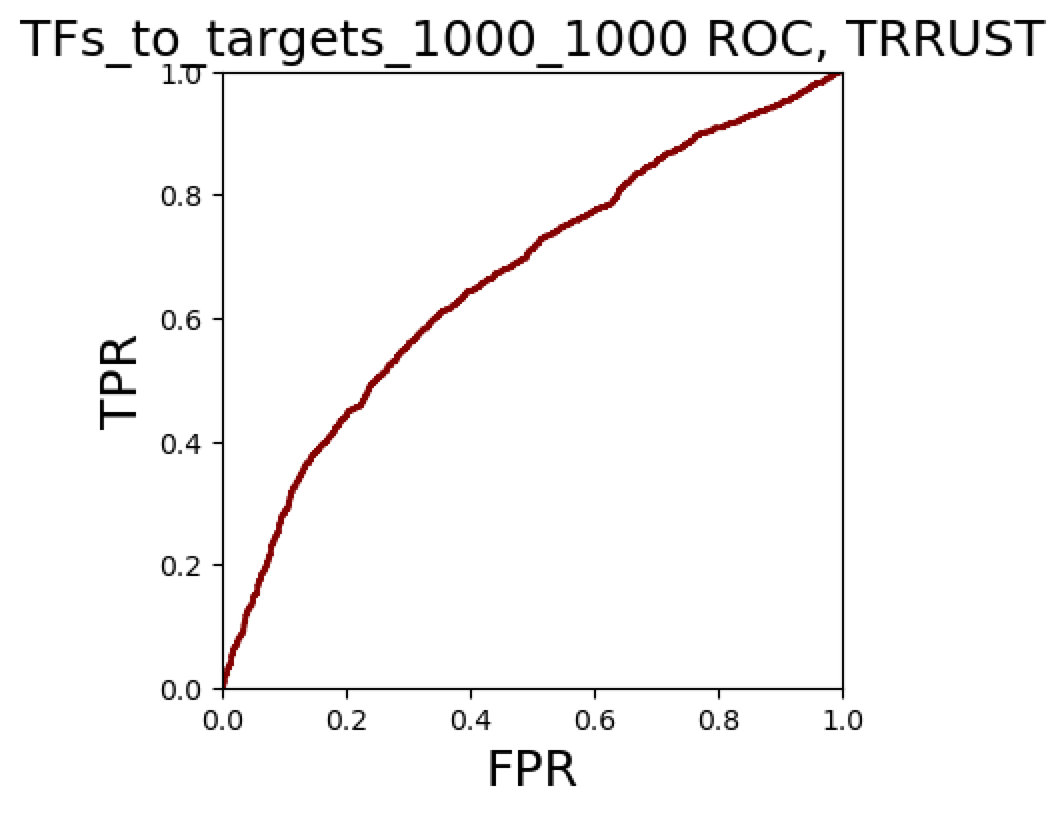
TFs_to_targets_1000_1000 ROC, TRRUST
TPR
FPR
0.0
0.2
0.4
0.6
0.8
1.0
0.0
0.2
0.4
0.6
0.8
1.0

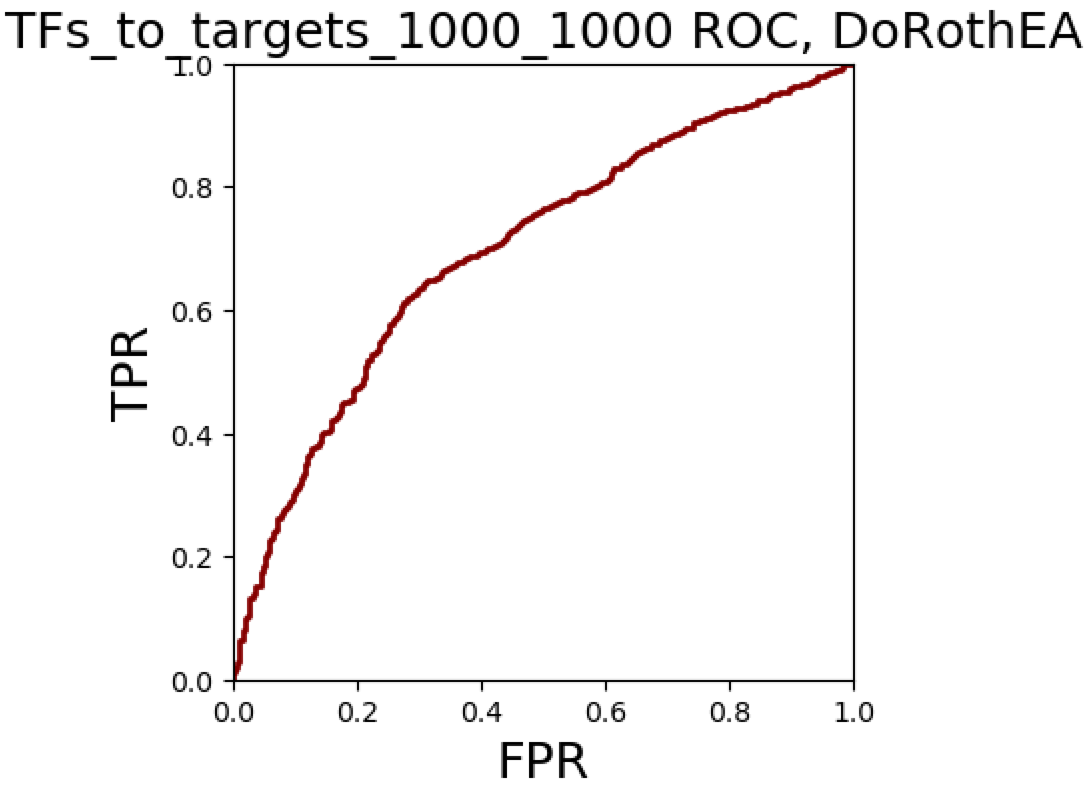

TFs_to_targets_1000_1000 ROC, DoRothEA
TPR
FPR
0.0
0.2
0.4
0.6
0.8
1.0

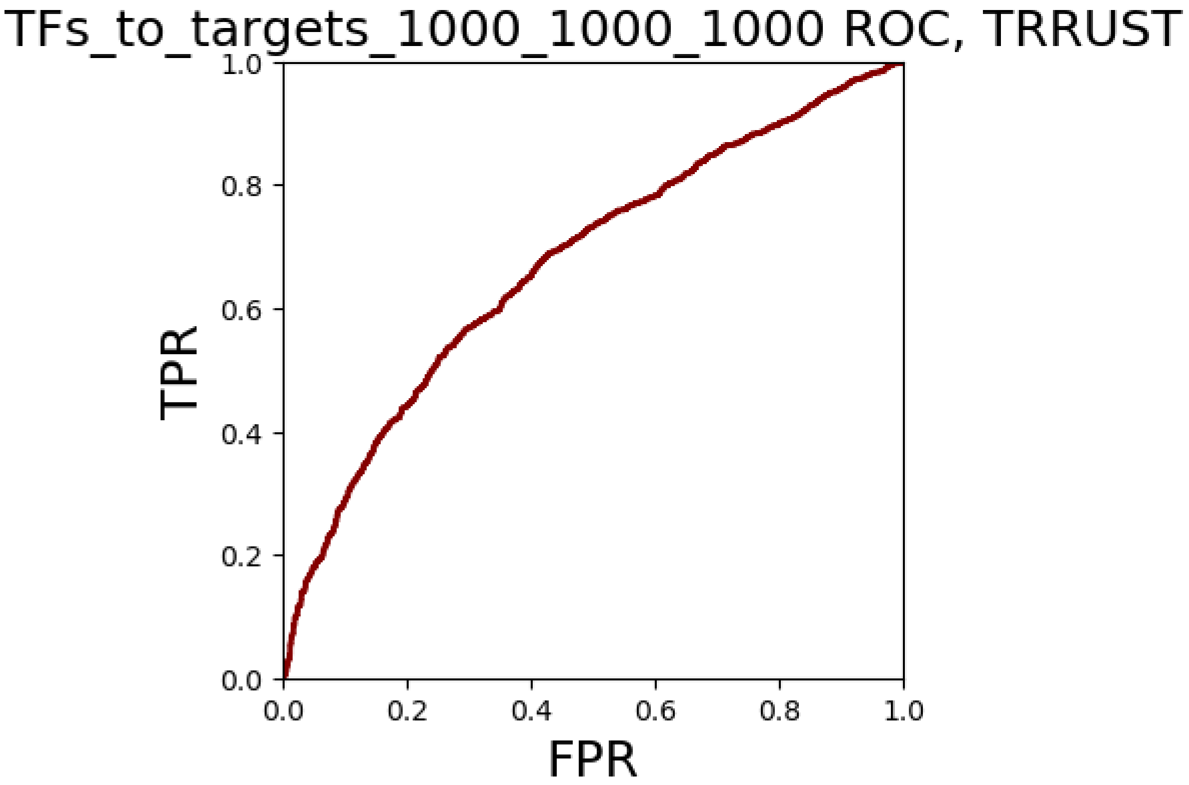
TFs_to_targets_1000_1000_1000 ROC, TRRUST
TPR
FPR
0.0
0.2
0.4
0.6
0.8
1.0
0.0
0.2
0.4
0.6
0.8
1.0

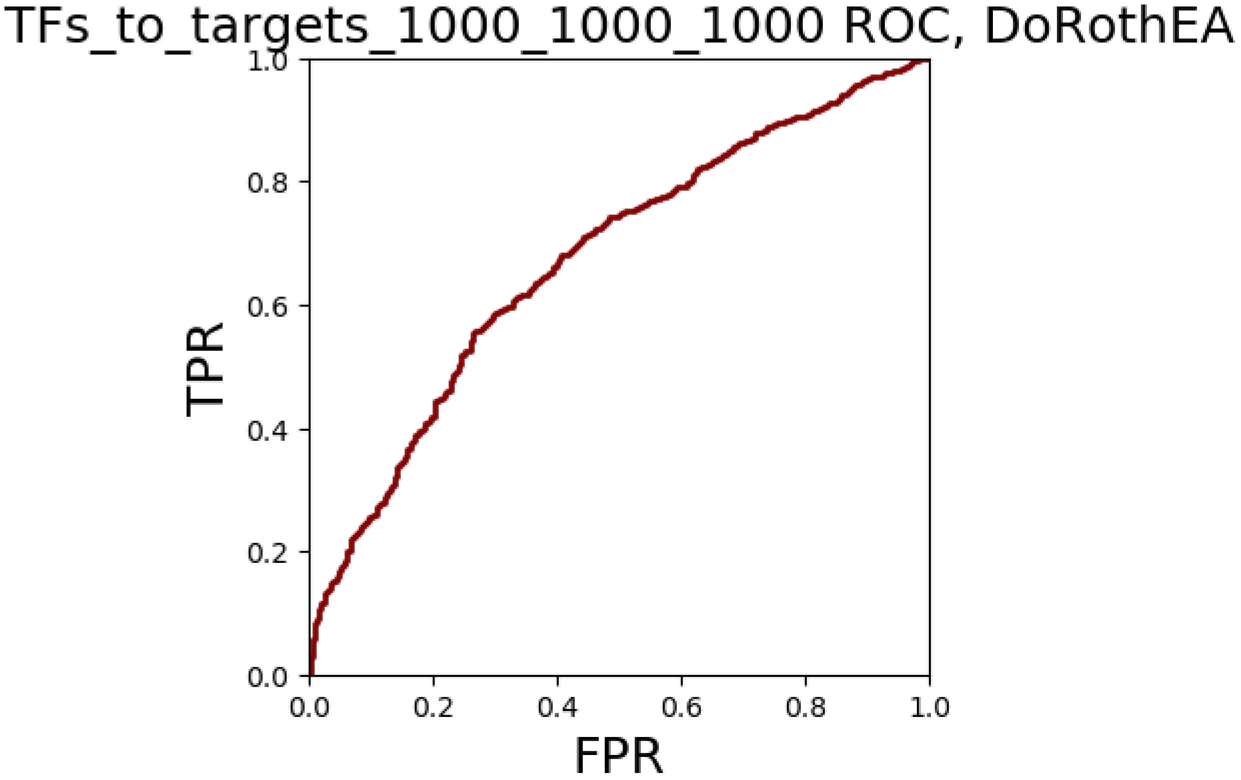
TFs_to_targets_1000_1000_1000 ROC, DoRothEA
TPR
FPR
0.0
0.2
0.4
0.6
0.8
1.0

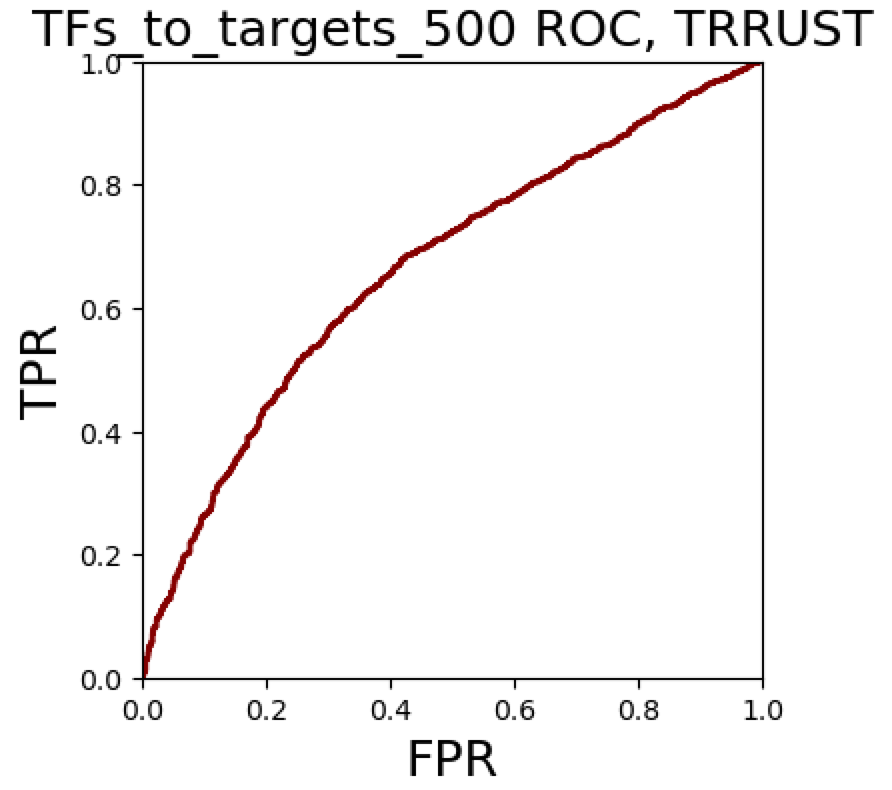

TFs_to_targets_500 ROC, TRRUST
TPR
FPR
0.0
0.2
0.4
0.6
0.8
1.0

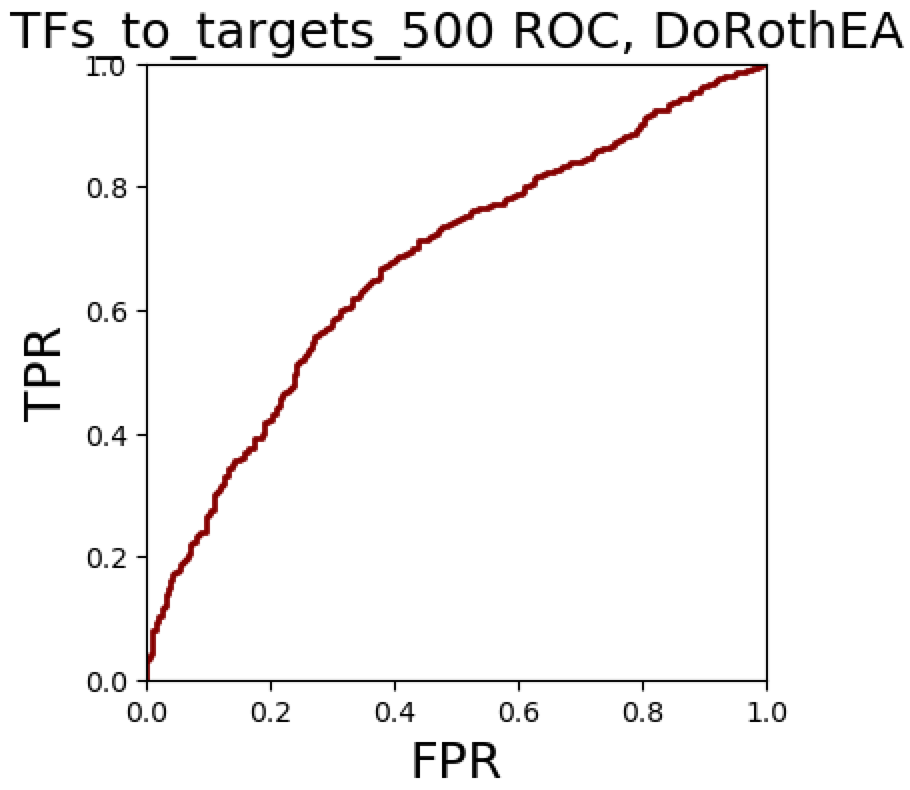
TFs_to_targets_500 ROC, DoRothEA
TPR
FPR
0.0
0.2
0.4
0.6
0.8
1.0

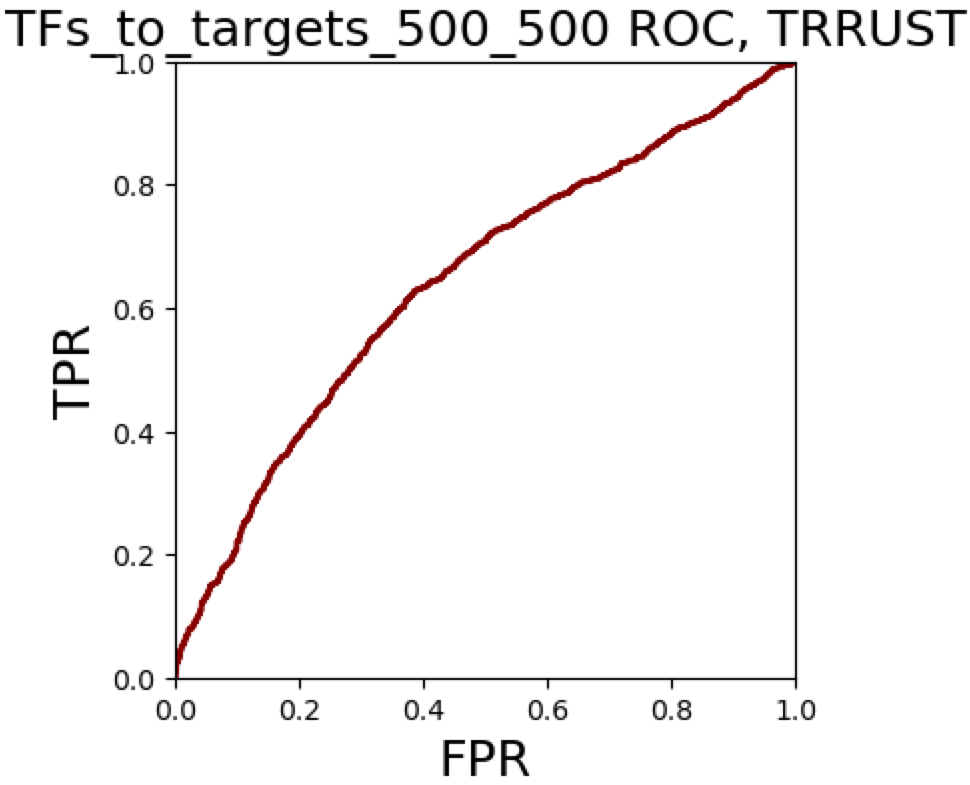
TFs_to_targets_500_500 ROC, TRRUST
TPR
FPR
0.0
0.2
0.4
0.6
0.8
1.0
0.0
0.2
0.4
0.6
0.8
1.0

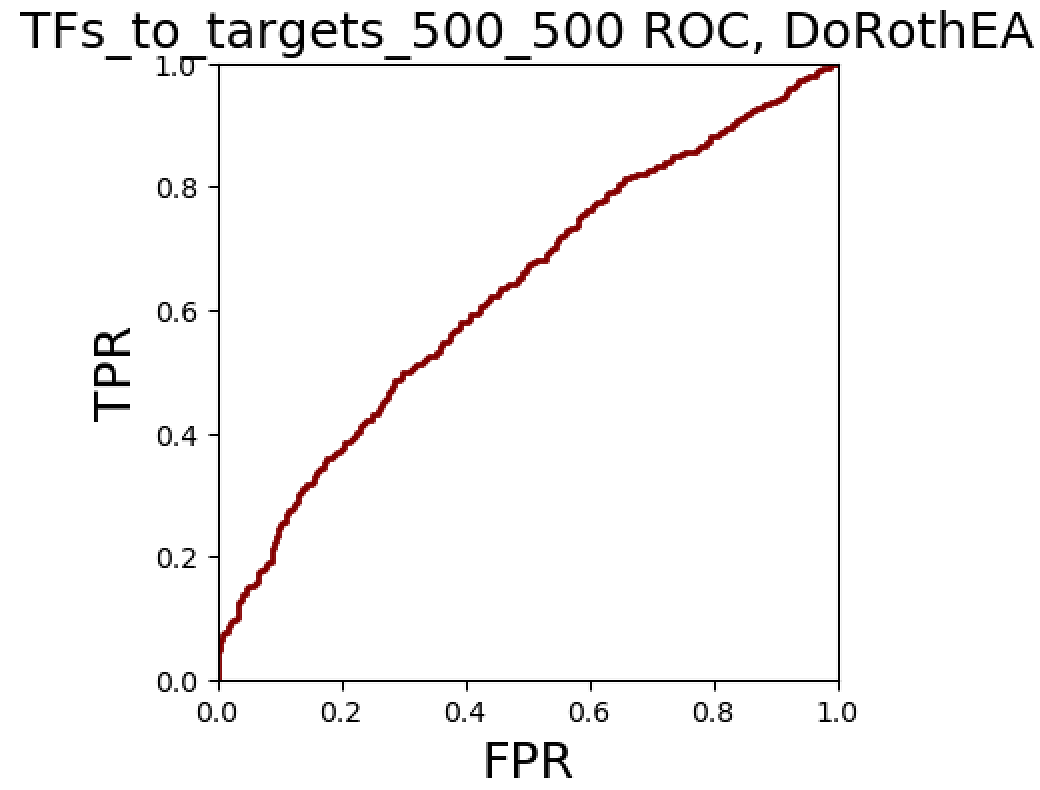
TFs_to_targets_500_500 ROC, DoRothEA
TPR
FPR
0.0
0.2
0.4
0.6
0.8
1.0

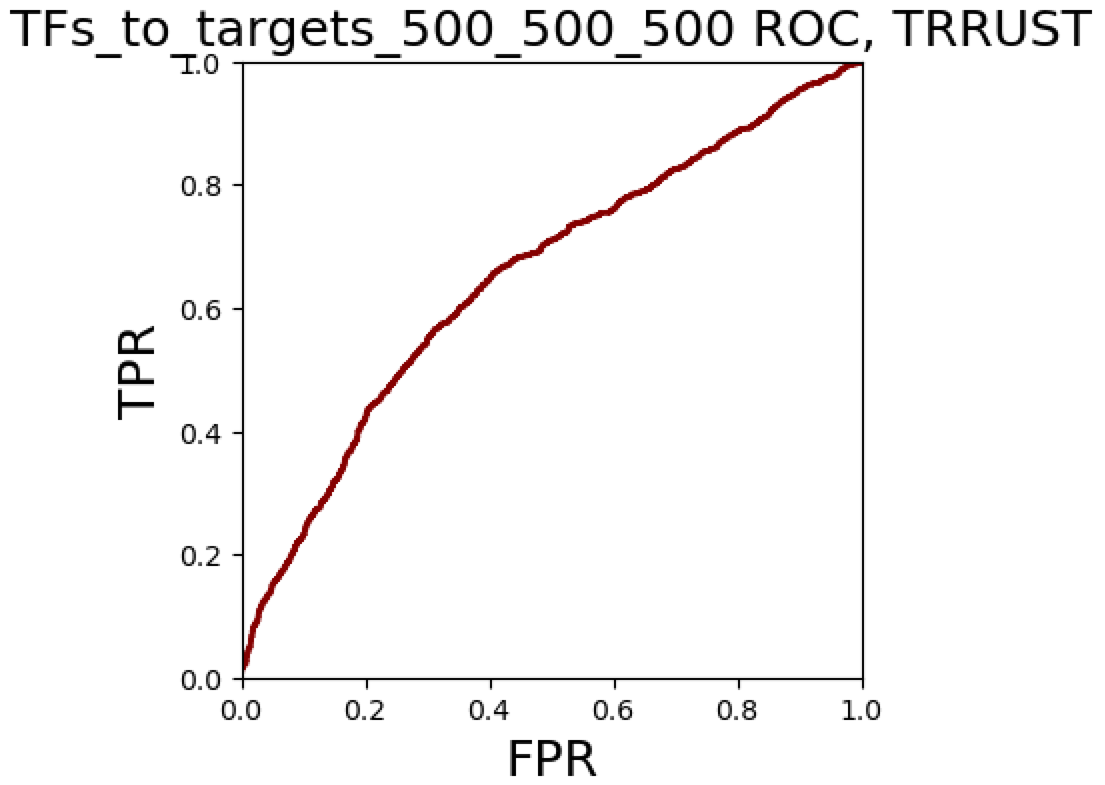

TFs_to_targets_500_500_500 ROC, TRRUST
TPR
FPR
0.0
0.2
0.4
0.6
0.8
1.0

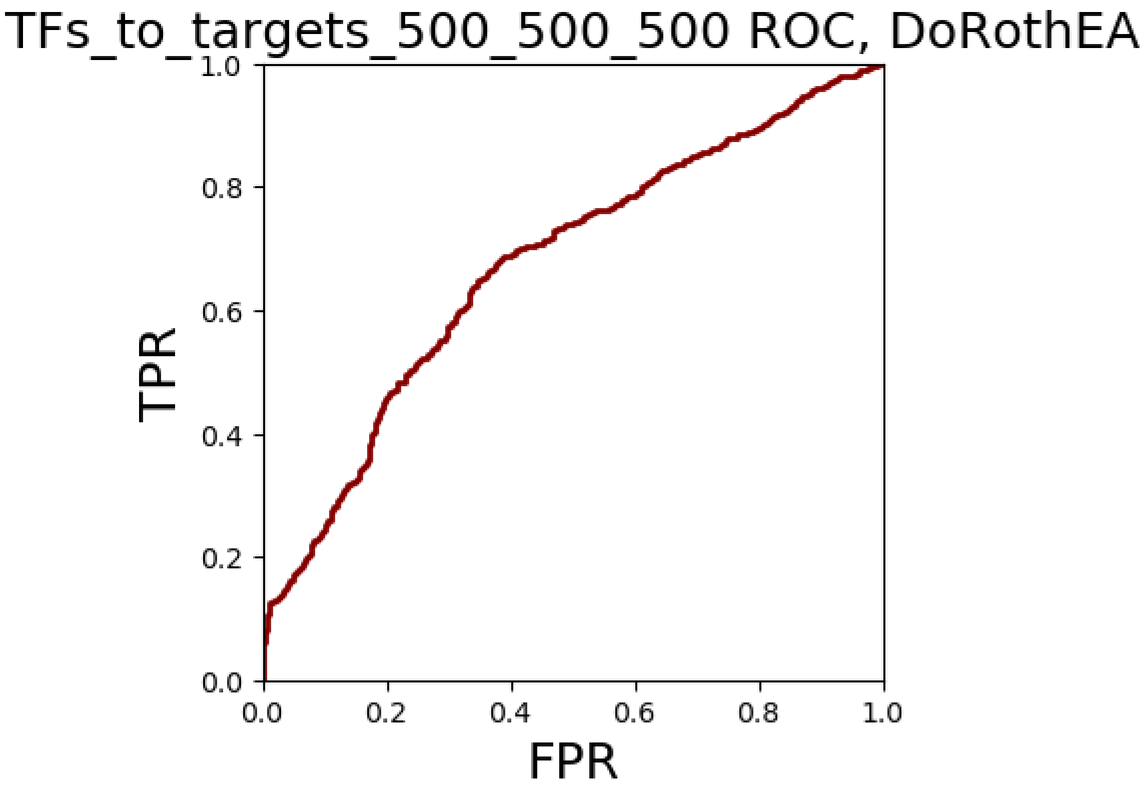
TFs_to_targets_500_500_500 ROC, DoRothEA
TPR
FPR
0.0
0.2
0.4
0.6
0.8
1.0

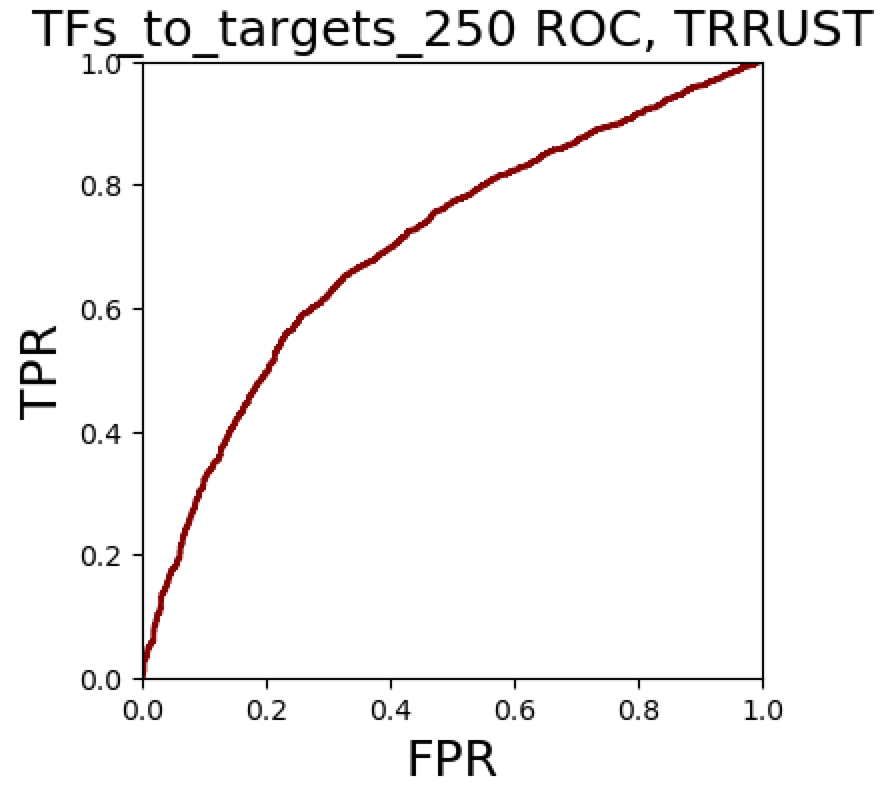

TFs_to_targets_250 ROC, TRRUST
TPR
FPR
0.0
0.2
0.4
0.6
0.8
1.0

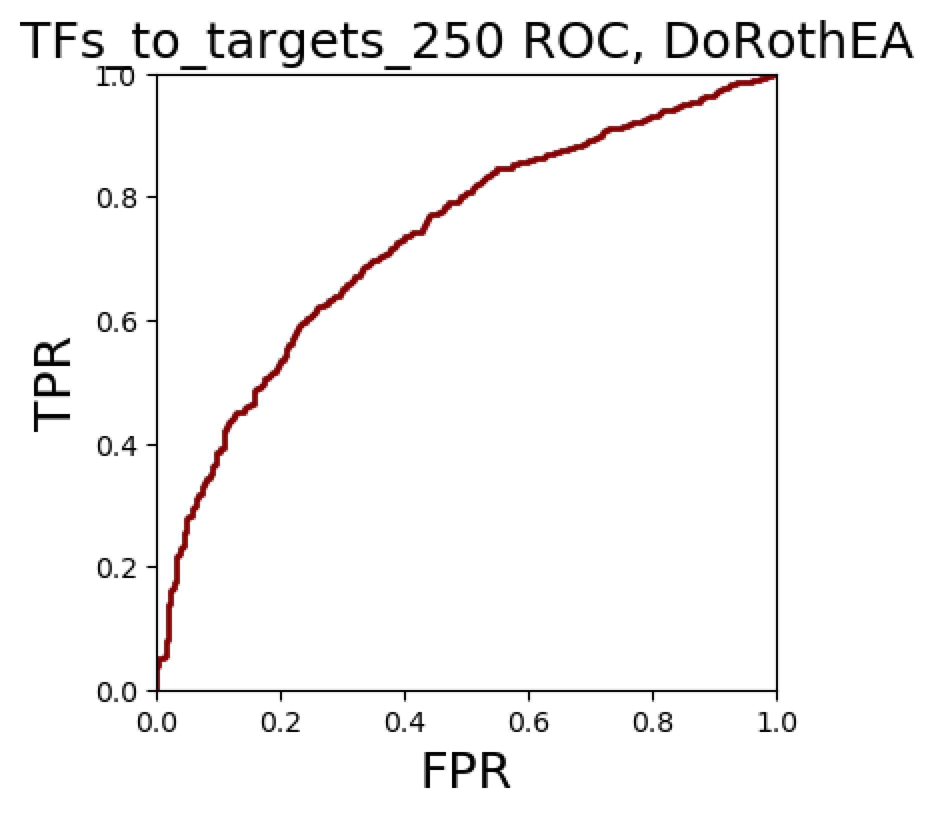

TFs_to_targets_250 ROC, DoRothEA
TPR
FPR
0.0
0.2
0.4
0.6
0.8
1.0

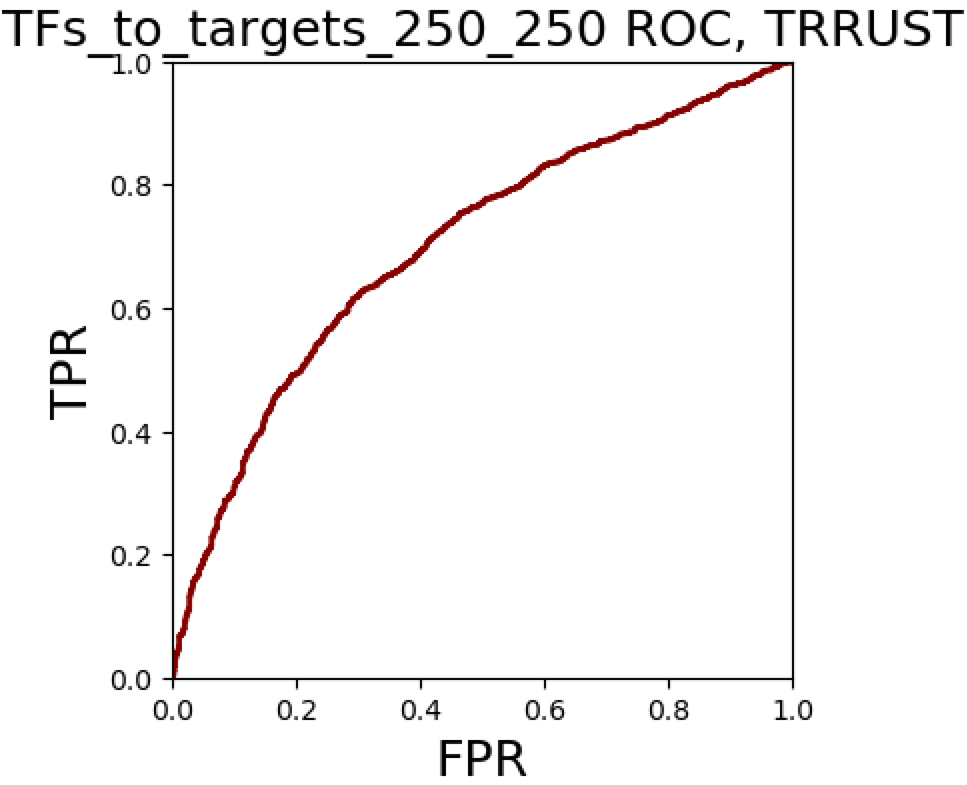
TFs_to_targets_250_250 ROC, TRRUST
TPR
FPR
0.0
0.2
0.4
0.6
0.8
1.0
0.0
0.2
0.4
0.6
0.8
1.0

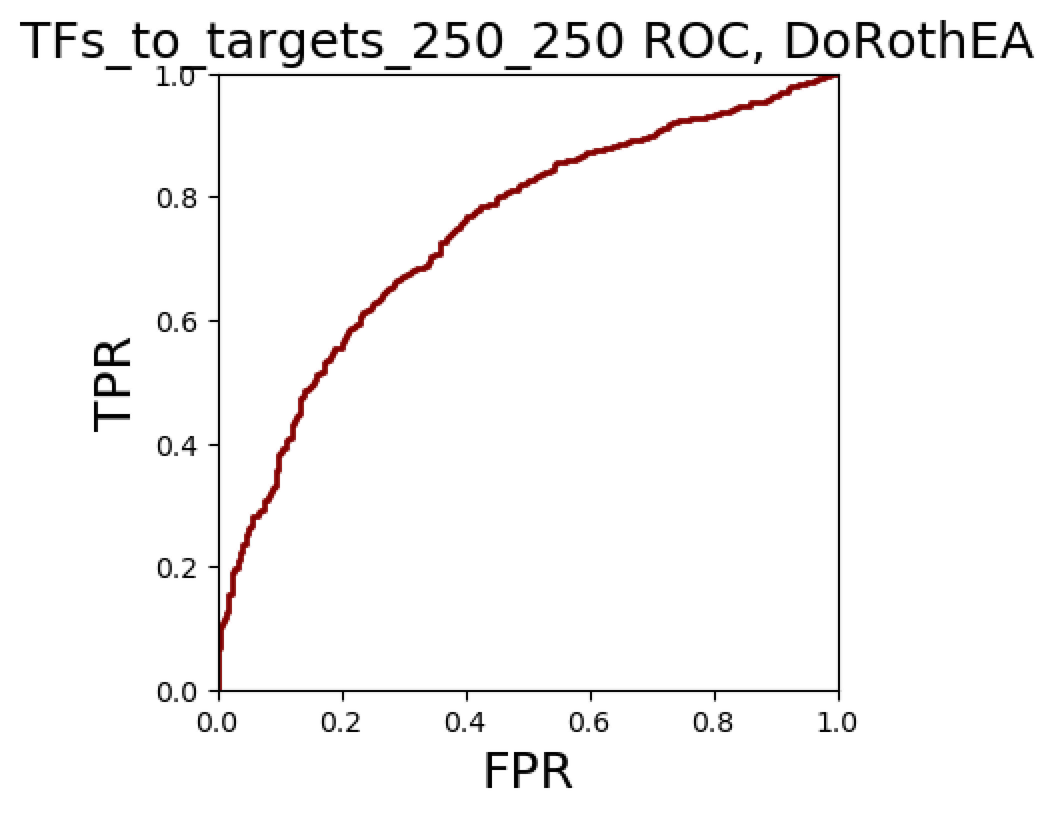
TFs_to_targets_250_250 ROC, DoRothEA
TPR
FPR
0.0
0.2
0.4
0.6
0.8
1.0

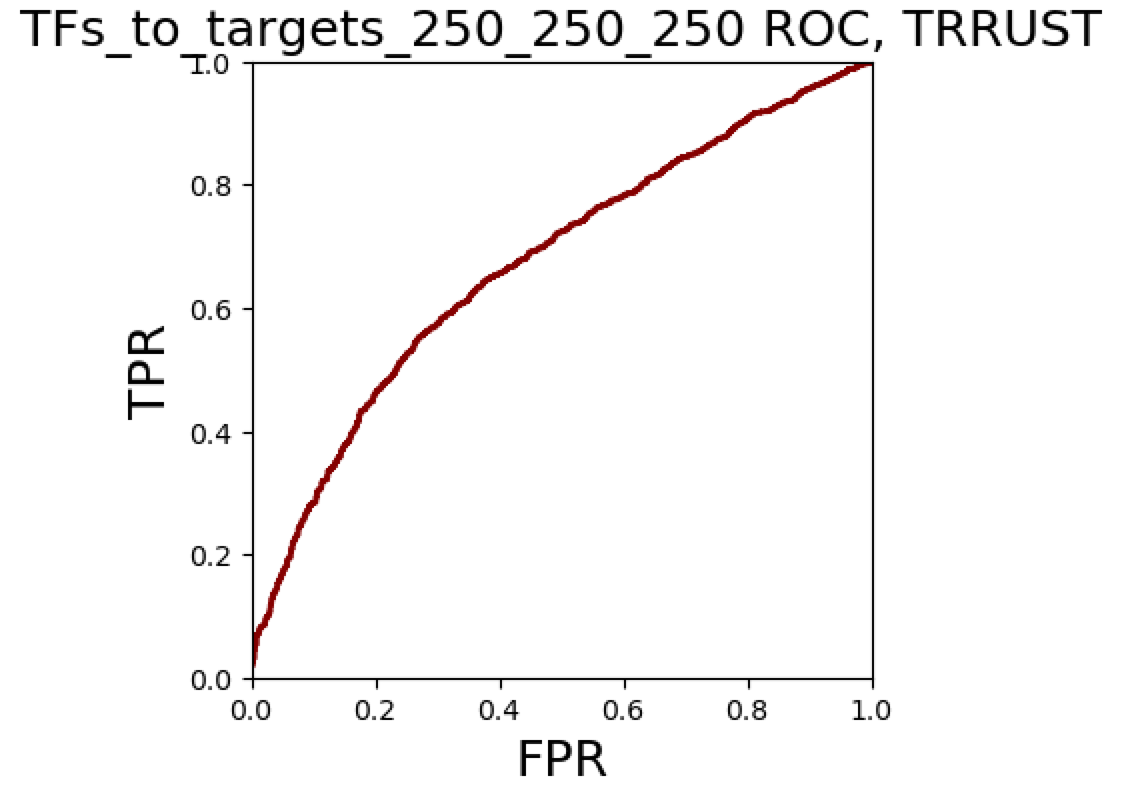

TFs_to_targets_250_250_250 ROC, TRRUST
TPR
FPR
0.0
0.2
0.4
0.6
0.8
1.0
0.0
0.2
0.4
0.6
0.8
1.0

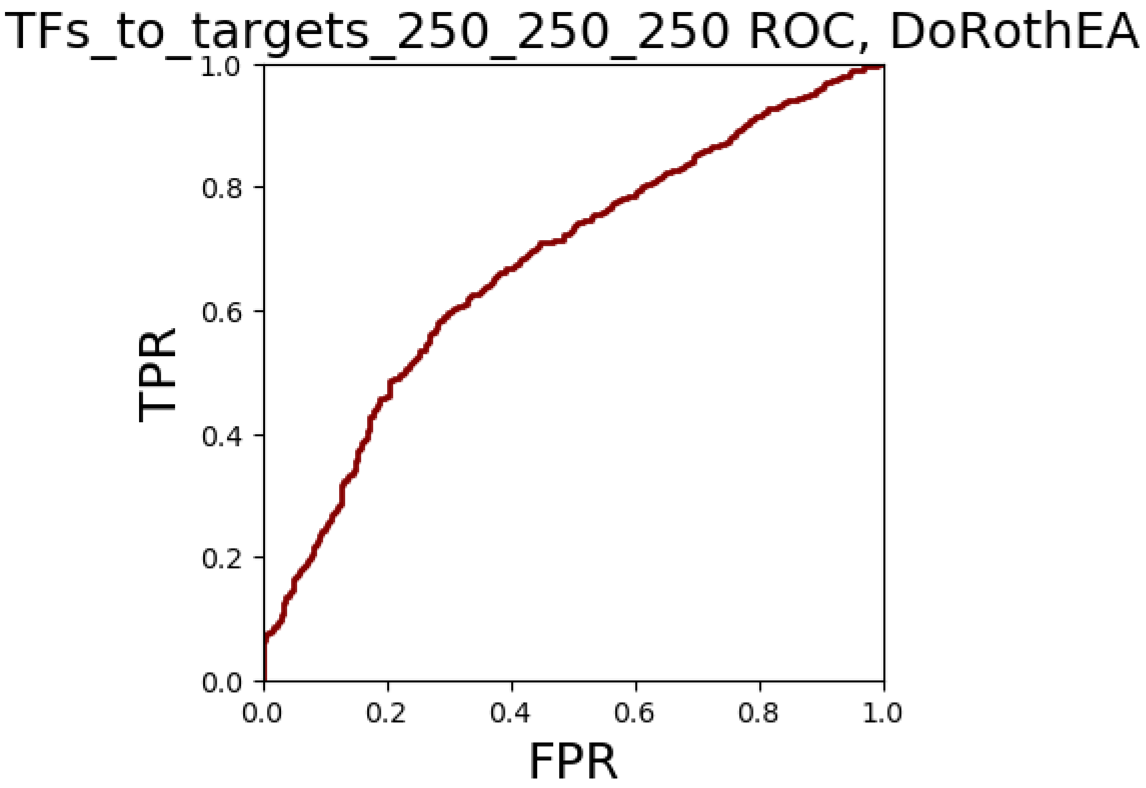
TFs_to_targets_250_250_250 ROC, DoRothEA
TPR
FPR
0.0
0.2
0.4
0.6
0.8
1.0

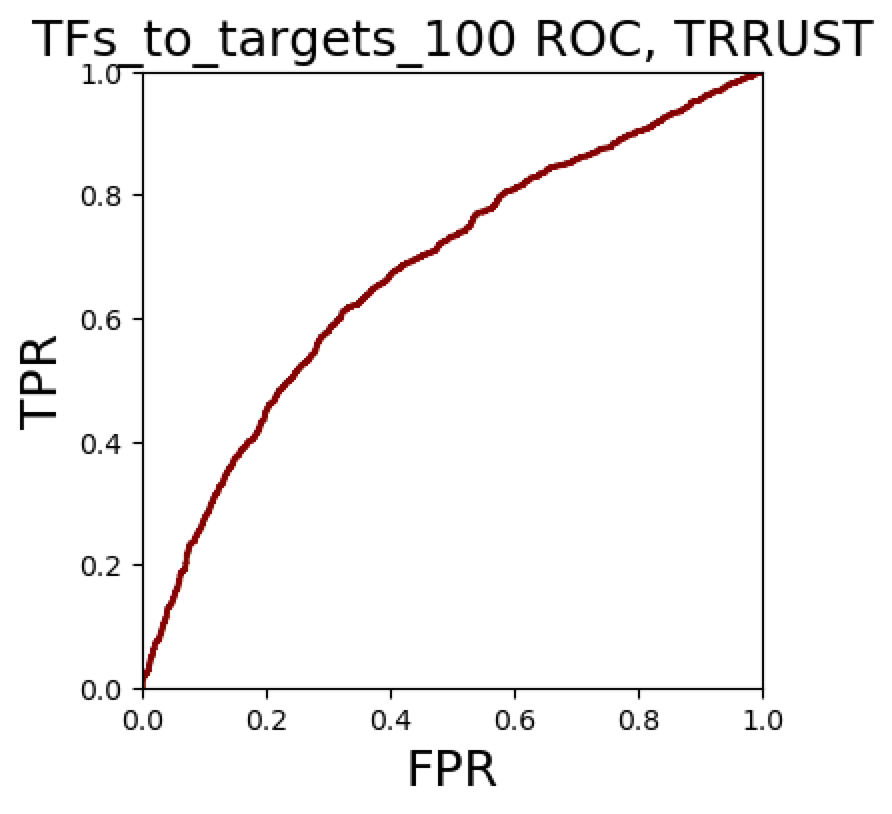

TFs_to_targets_100 ROC, TRRUST
TPR
FPR
0.0
0.2
0.4
0.6
0.8
1.0
0.0
0.2
0.4
0.6
0.8
1.0

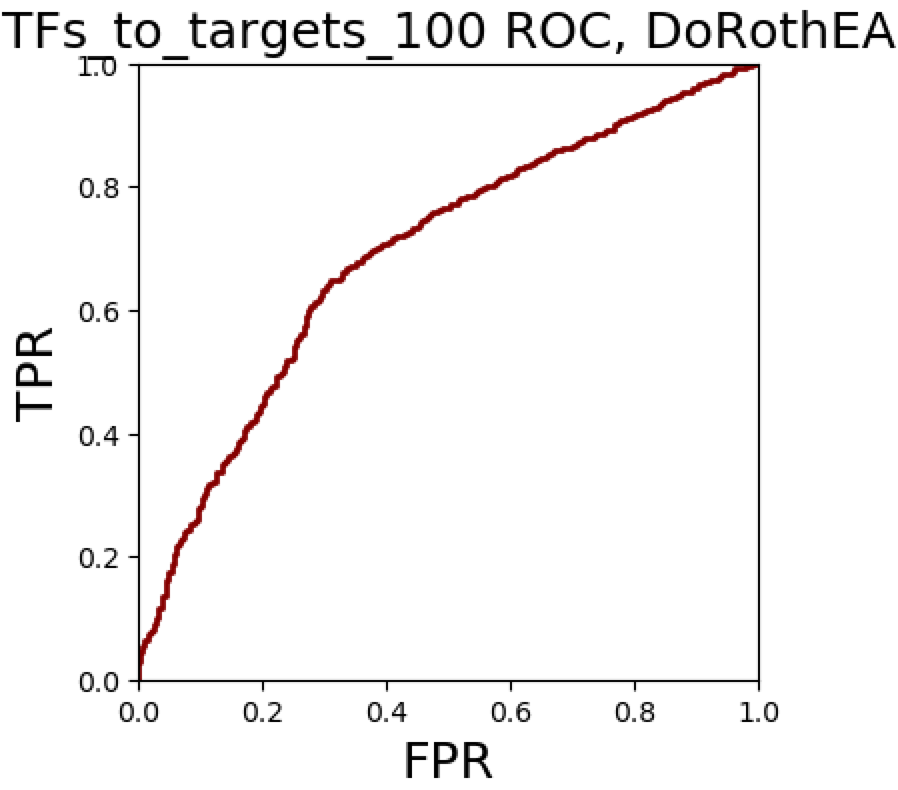
TFs_to_targets_100 ROC, DoRothEA
TPR
FPR
0.0
0.2
0.4
0.6
0.8
1.0

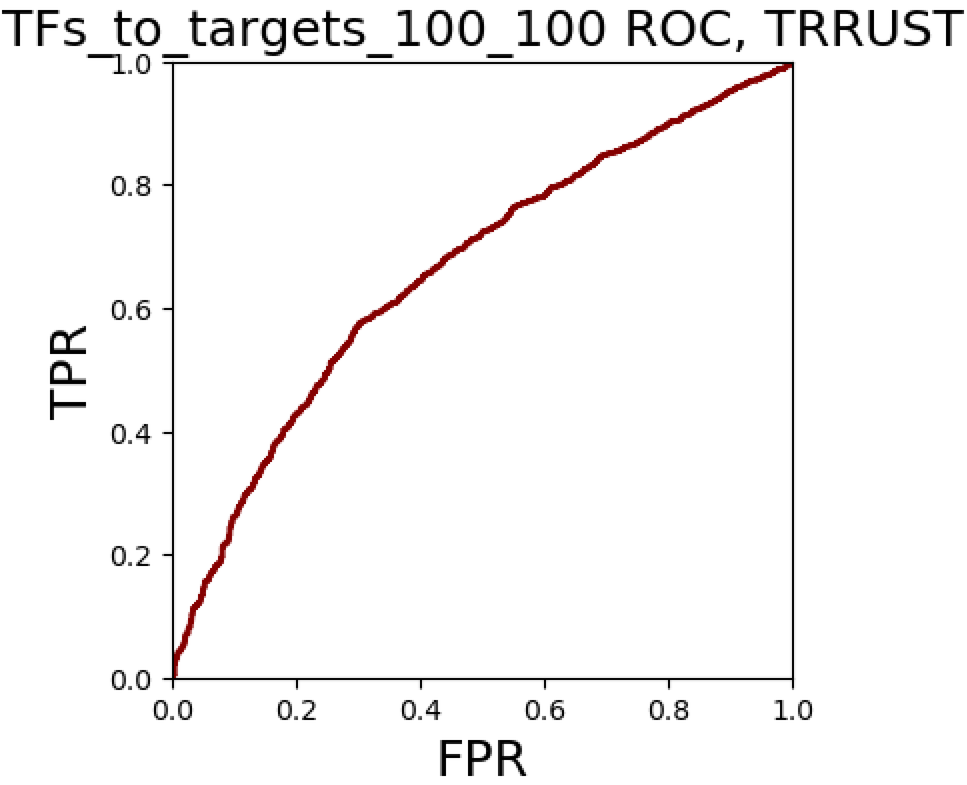
TFs_to_targets_100_100 ROC, TRRUST
TPR
FPR
0.0
0.2
0.4
0.6
0.8
1.0

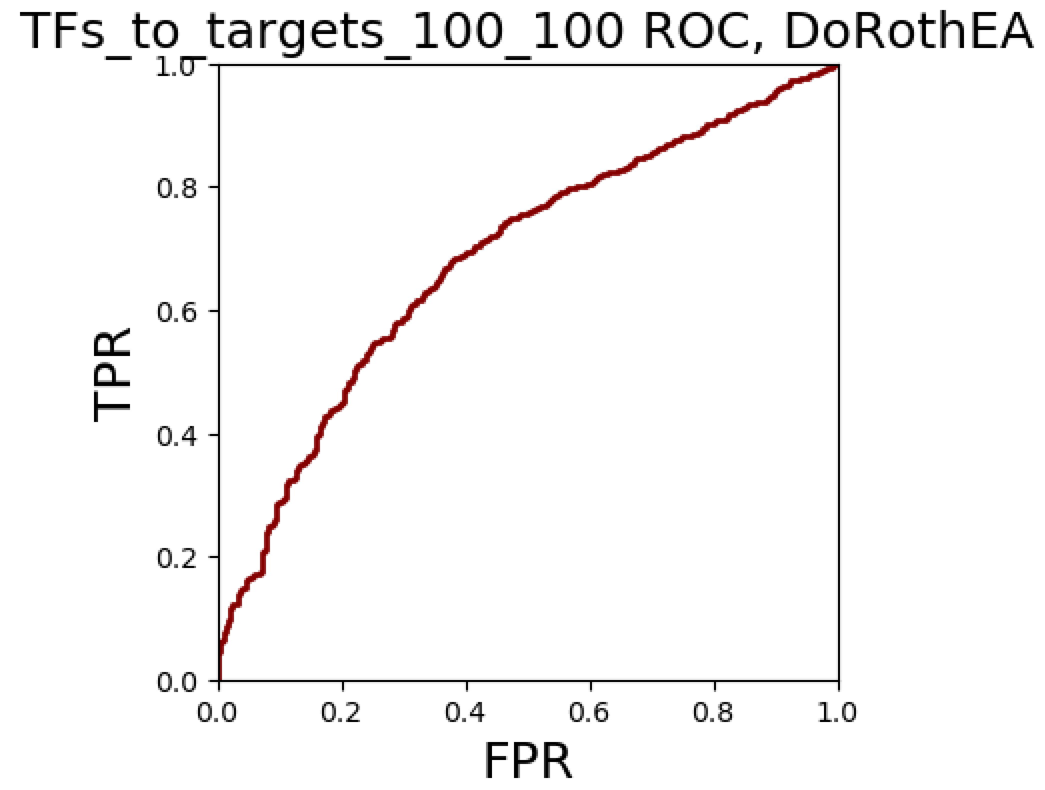

TFs_to_targets_100_100 ROC, DoRothEA
TPR
FPR
0.0
0.2
0.4
0.6
0.8
1.0

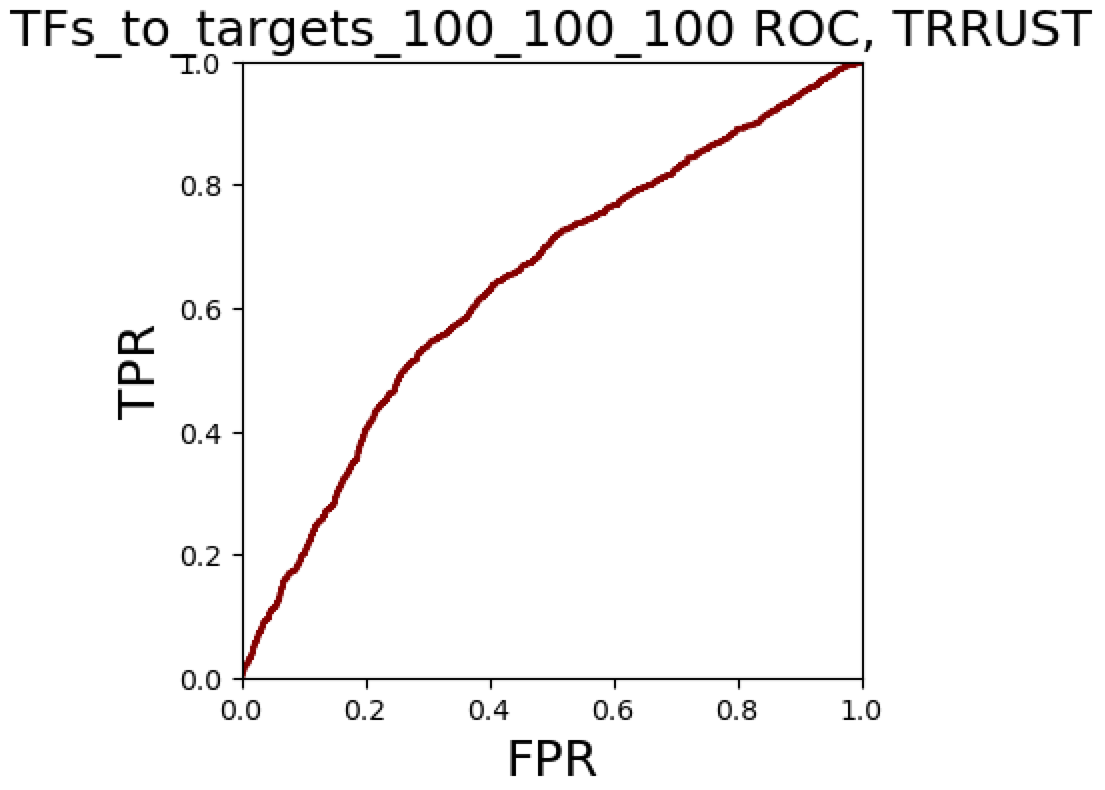
TFs_to_targets_100_100_100 ROC, TRRUST
TPR
FPR
0.0
0.2
0.4
0.6
0.8
1.0

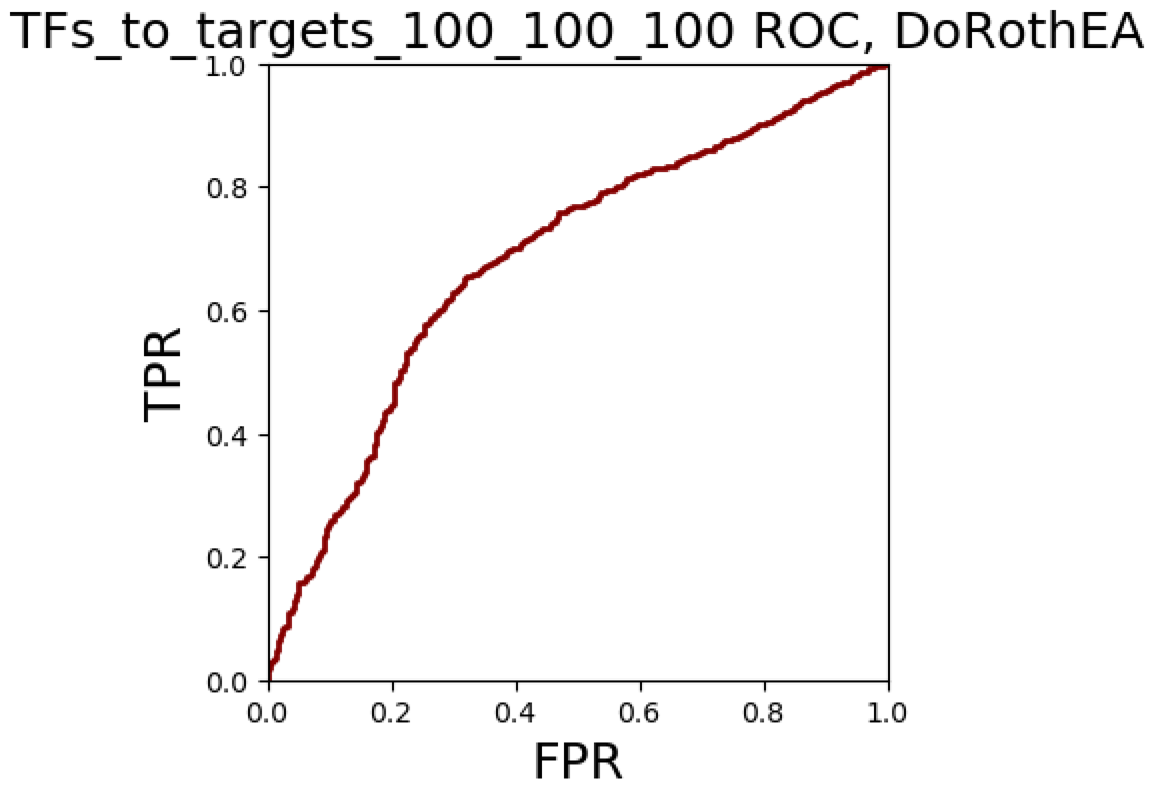
TFs_to_targets_100_100_100 ROC, DoRothEA
TPR
FPR
0.0
0.2
0.4
0.6
0.8
1.0

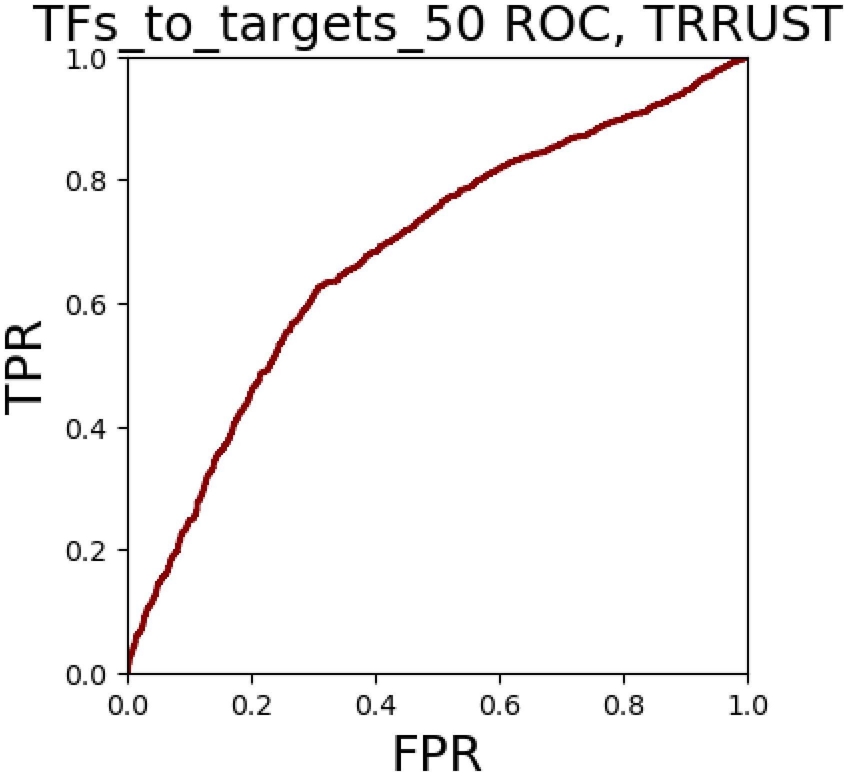

TFs_to_targets_50 ROC, TRRUST
TPR
FPR
0.0
0.2
0.4
0.6
0.8
1.0

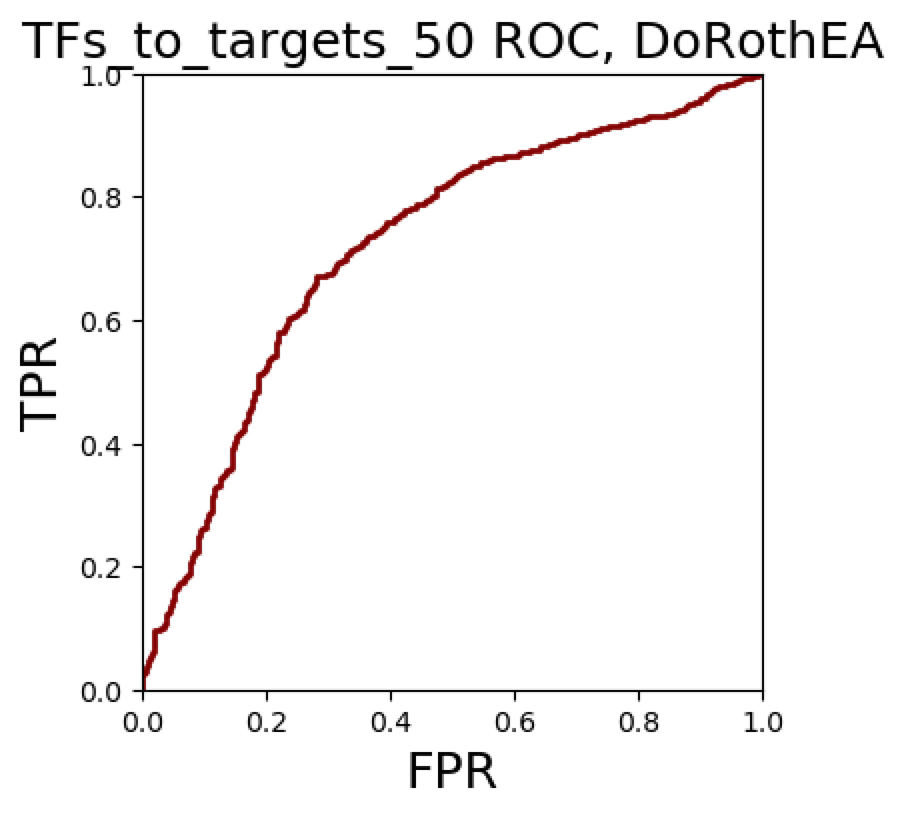
TFs_to_targets_50 ROC, DoRothEA
TPR
FPR
0.0
0.2
0.4
0.6
0.8
1.0

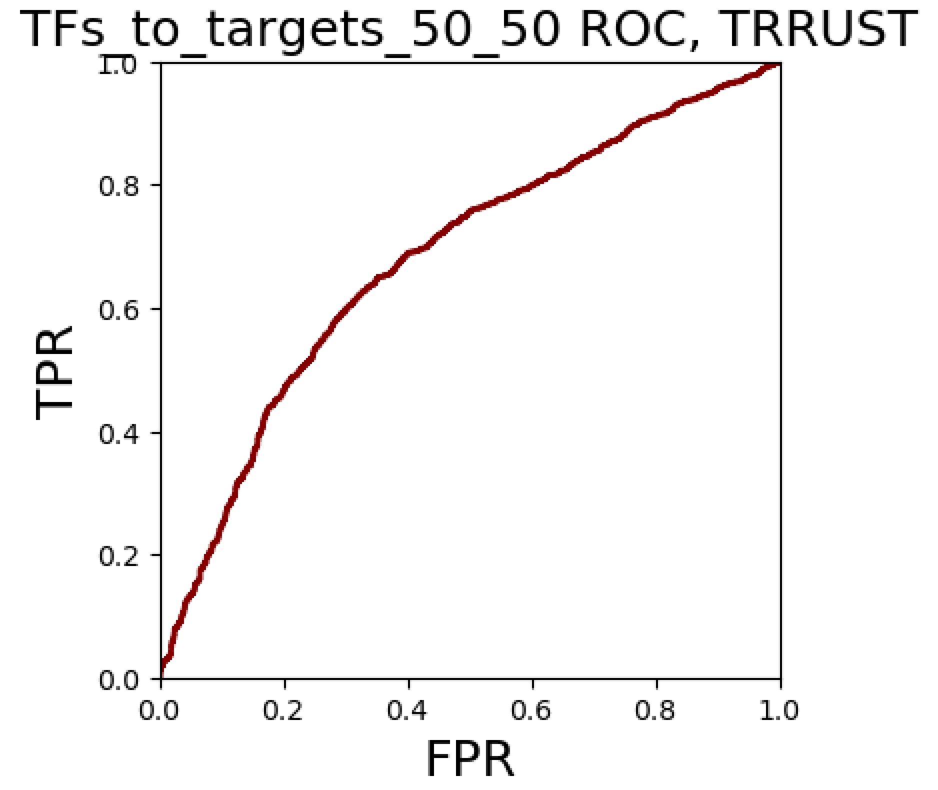
TFs_to_targets_50_50 ROC, TRRUST
TPR
FPR
0.0
0.2
0.4
0.6
0.8
1.0
0.0
0.2
0.4
0.6
0.8
1.0

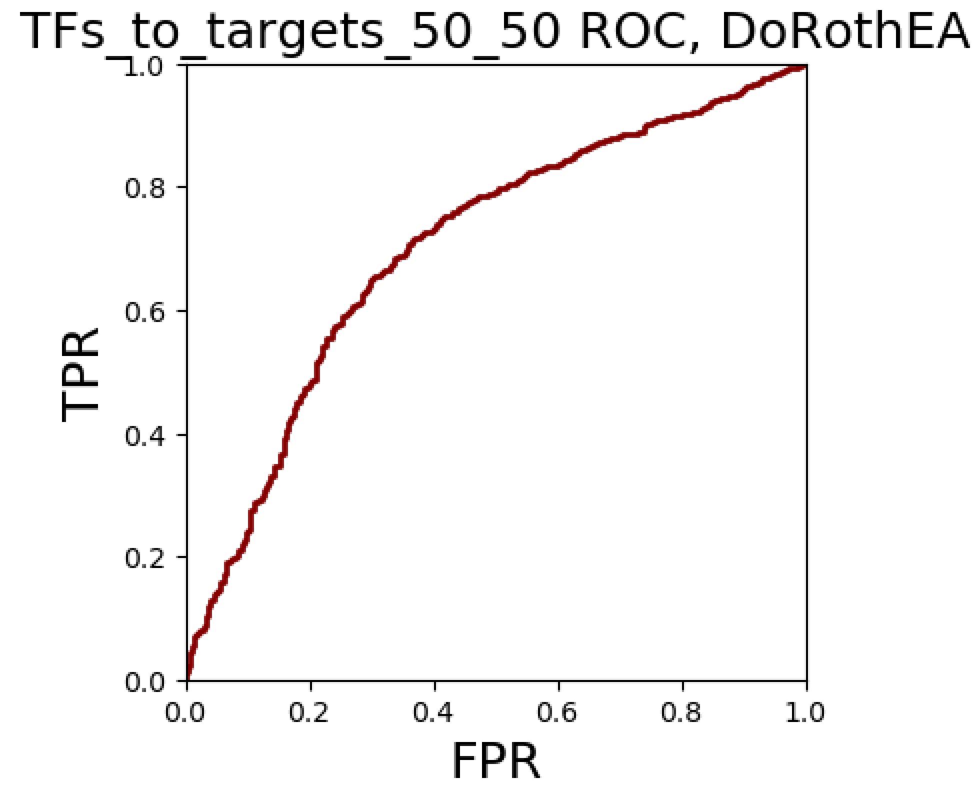
TFs_to_targets_50_50 ROC, DoRothEA
TPR
FPR
0.0
0.2
0.4
0.6
0.8
1.0

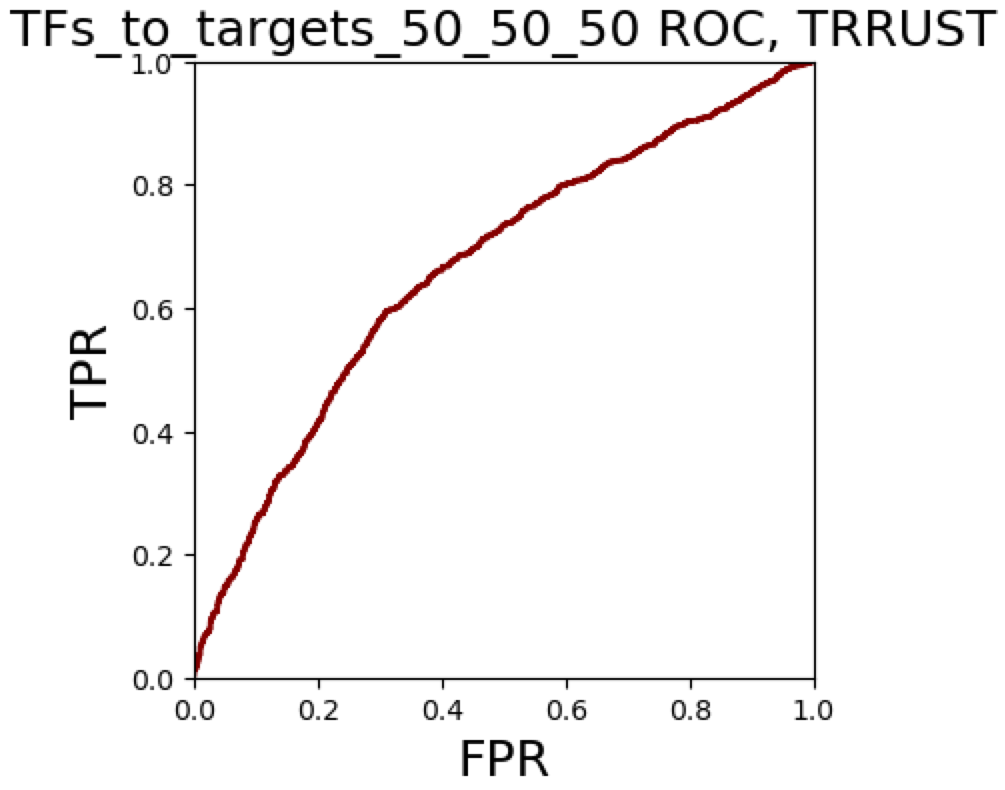
TFs_to_targets_50_50_50 ROC, TRRUST
TPR
FPR
0.0
0.2
0.4
0.6
0.8
1.0

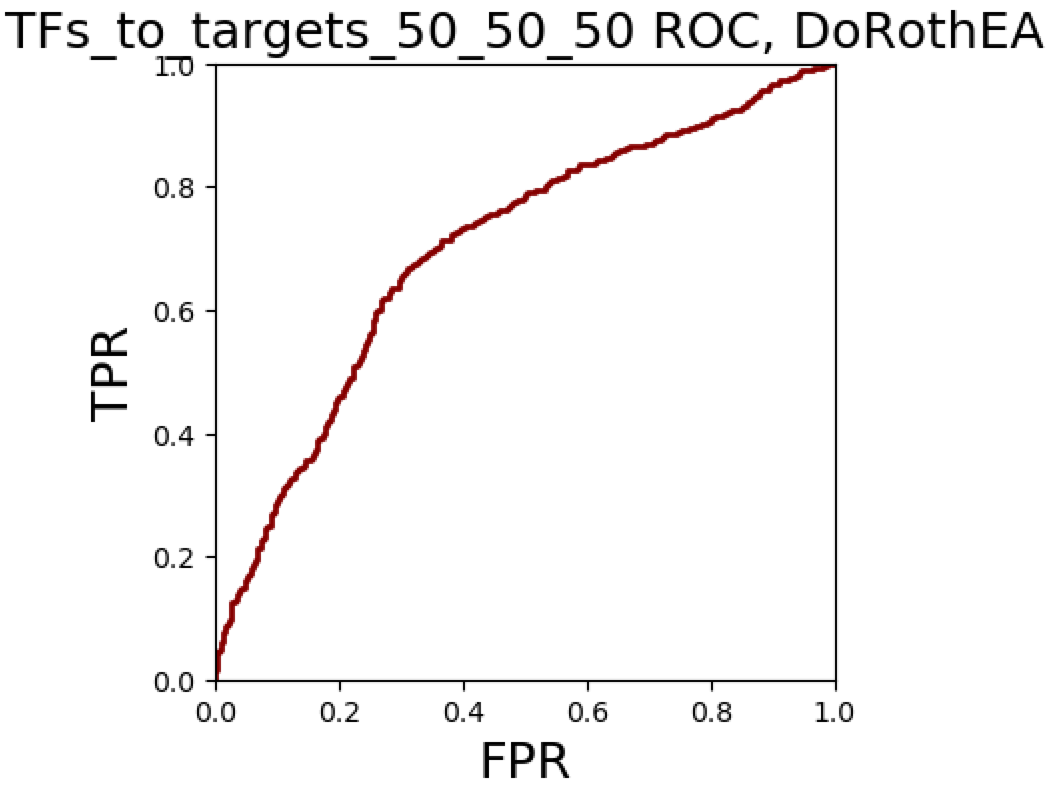

TFs_to_targets_50_50_50 ROC, DoRothEA
TPR
FPR
0.0
0.2
0.4
0.6
0.8
1.0

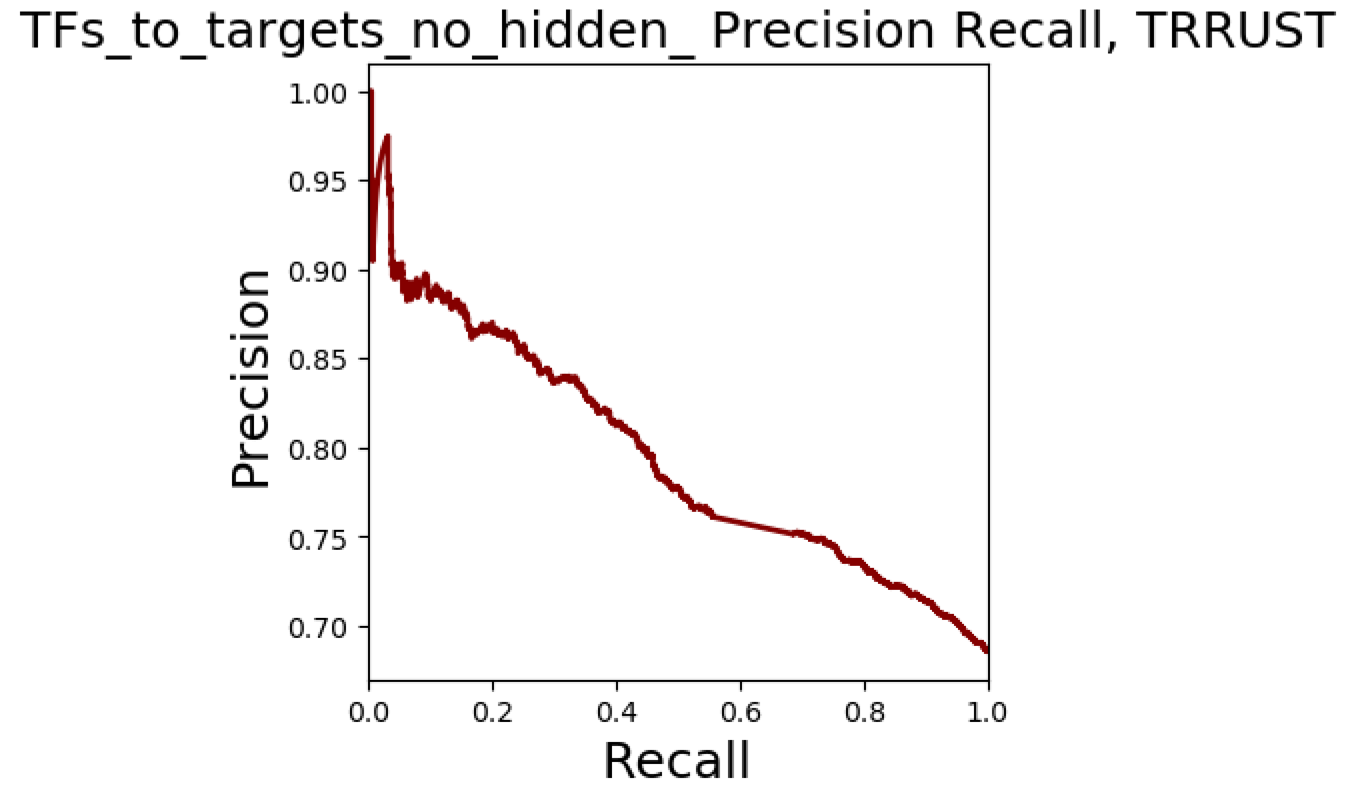

TFs_to_targets_no_hidden_ Precision Recall, TRRUST
Precision
1.00
0.95
0.90
0.85
0.80
0.75
0.70
Recall
0.0
0.2
0.4
0.6
0.8
1.0

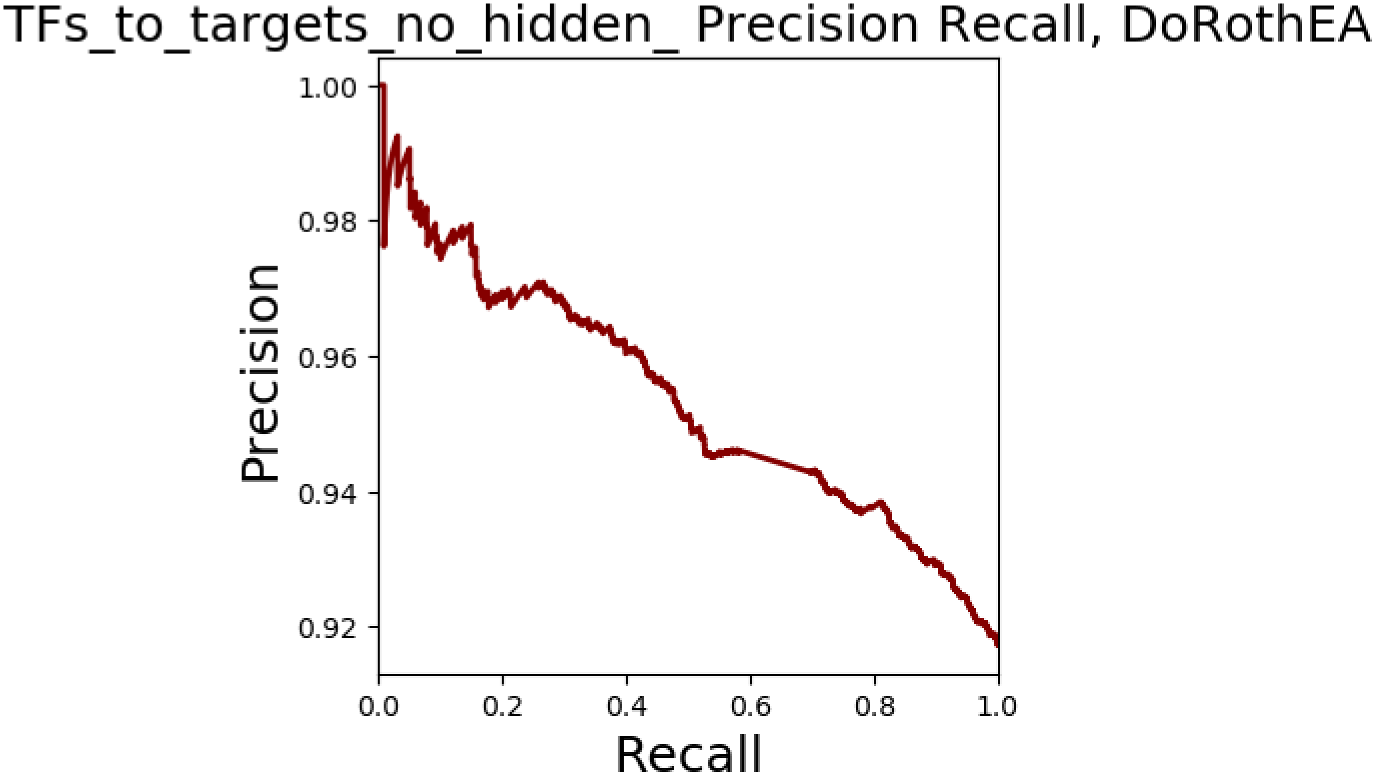

TFs_to_targets_no_hidden_ Precision Recall, DoRothEA
Precision
1.00
0.98
0.96
0.94
0.92
Recall
0.0
0.2
0.4
0.6
0.8
1.0

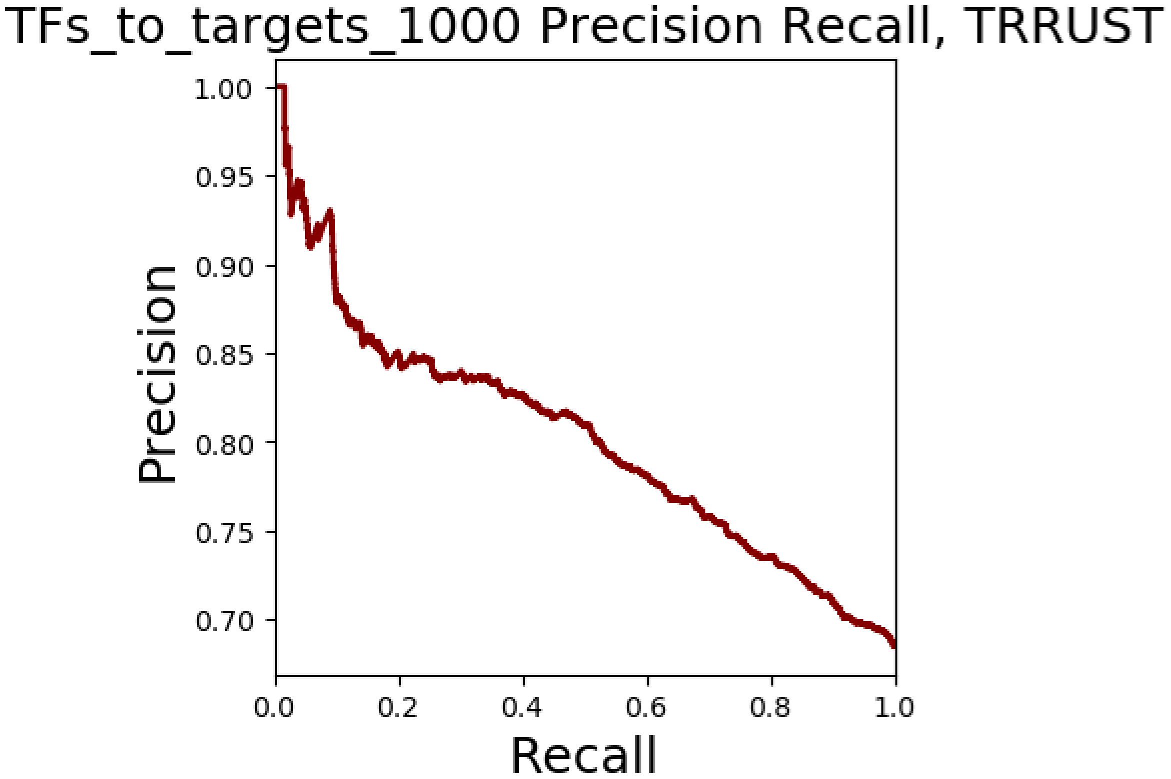
TFs_to_targets_1000 Precision Recall, TRRUST
Precision
Recall
1.00
0.95
0.90
0.85
0.80
0.75
0.70
0.0
0.2
0.4
0.6
0.8
1.0

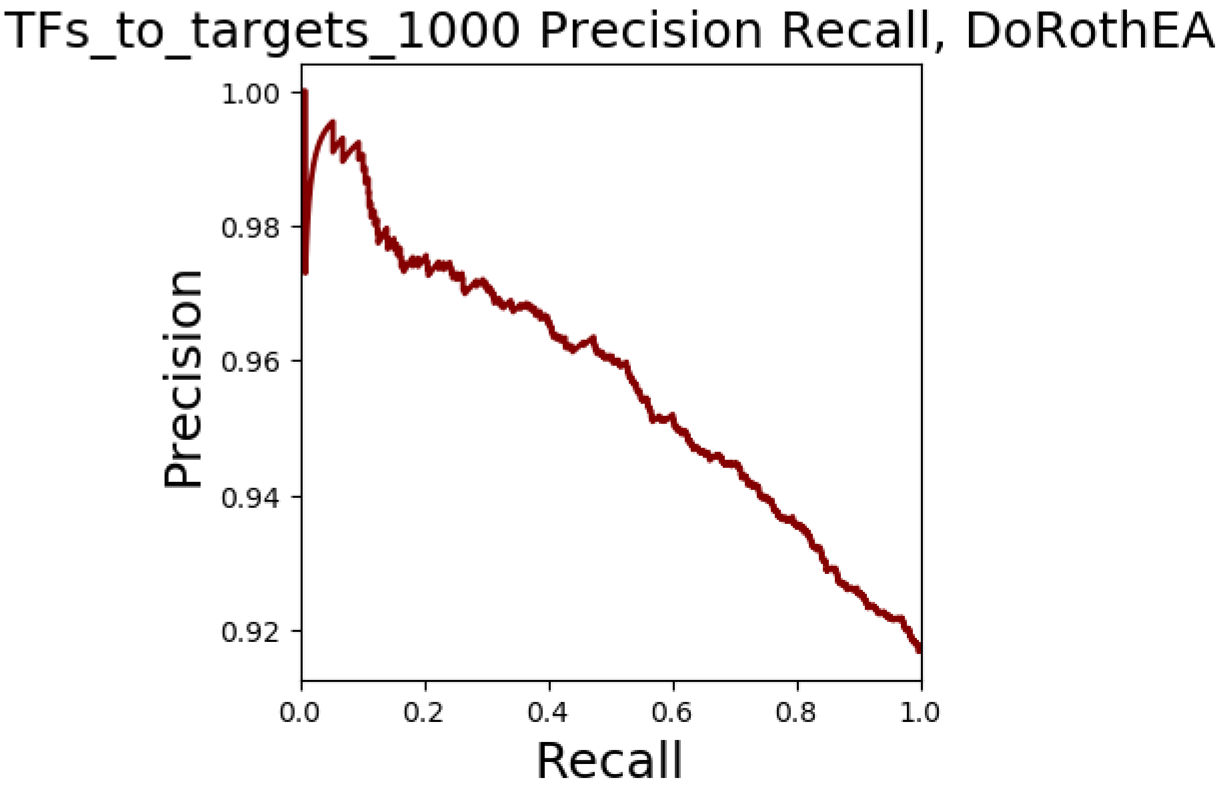
TFs_to_targets_1000 Precision Recall, DoRothEA
Precision
1.00
0.98
0.96
0.94
0.92
Recall
0.0
0.2
0.4
0.6
0.8
1.0

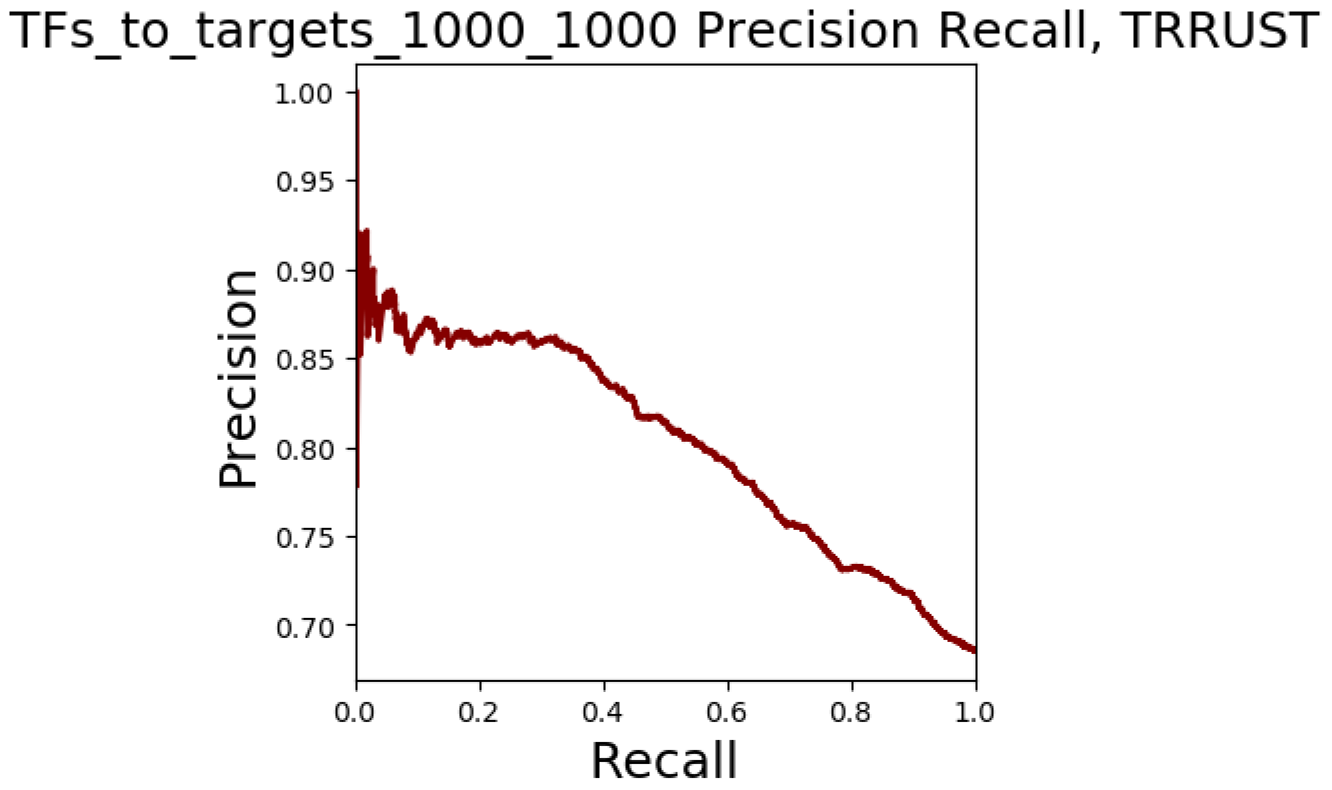
TFs_to_targets_1000_1000 Precision Recall, TRRUST
Precision
Recall
1.00
0.95
0.90
0.85
0.80
0.75
0.70
0.0
0.2
0.4
0.6
0.8
1.0

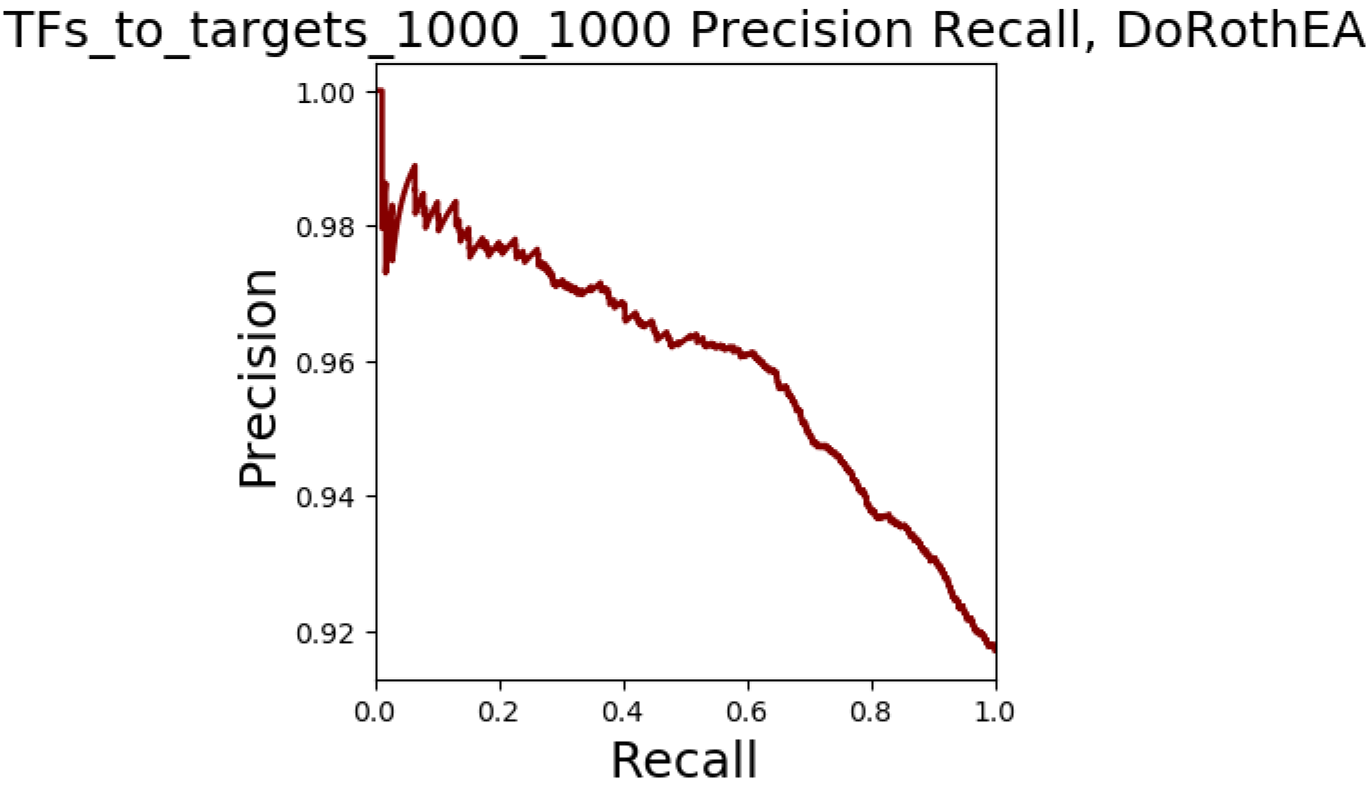
TFs_to_targets_1000_1000 Precision Recall, DoRothEA
Precision
Recall
1.00
0.98
0.96
0.94
0.92
0.0
0.2
0.4
0.6
0.8
1.0

TFs_to_targets_1000_1000_1000 Precision Recall, TRRUST

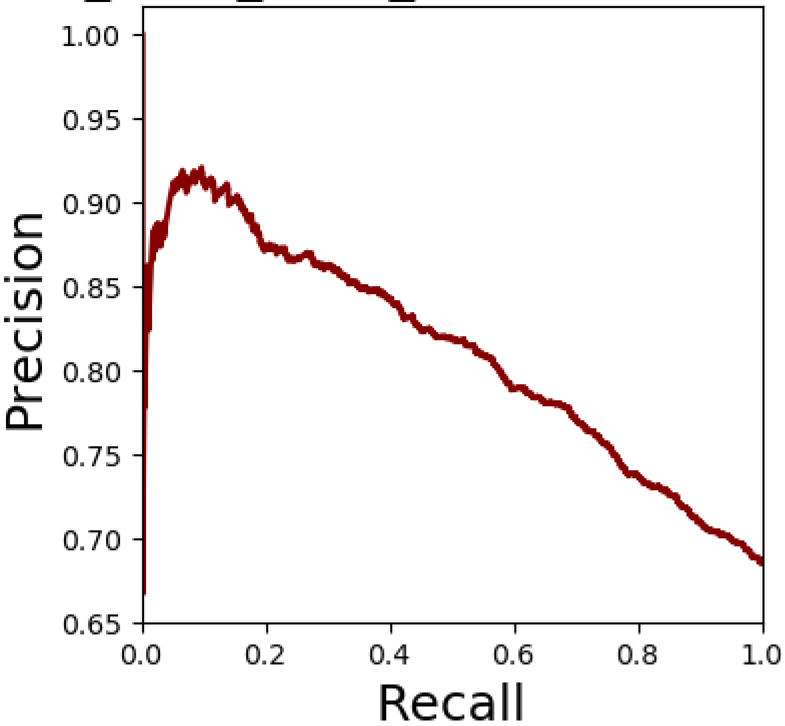

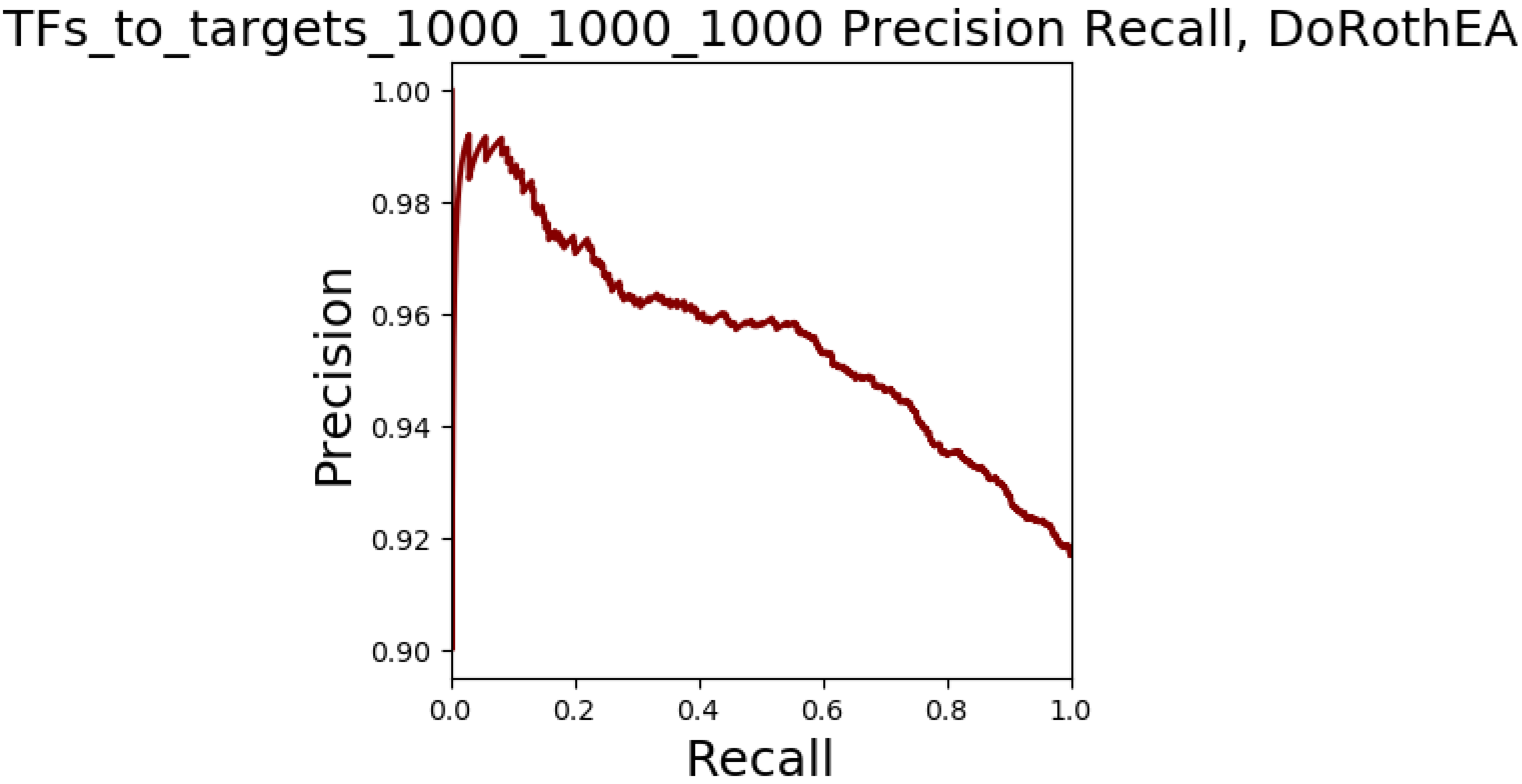

TFs_to_targets_1000_1000_1000 Precision Recall, DoRothEA
Precision
1.00
0.98
0.96
0.94
0.92
0.90
Recall
0.0
0.2
0.4
0.6
0.8
1.0

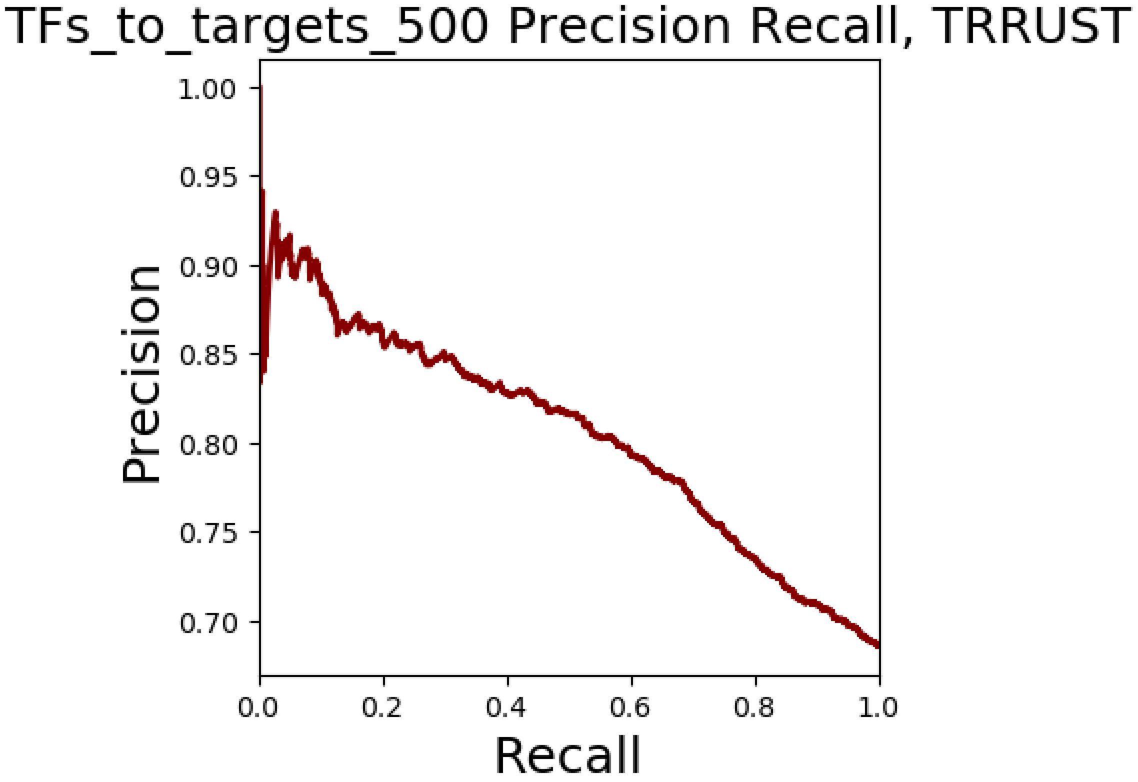

TFs_to_targets_500 Precision Recall, TRRUST
Precision
1.00
0.95
0.90
0.85
0.80
0.75
0.70
Recall
0.0
0.2
0.4
0.6
0.8
1.0

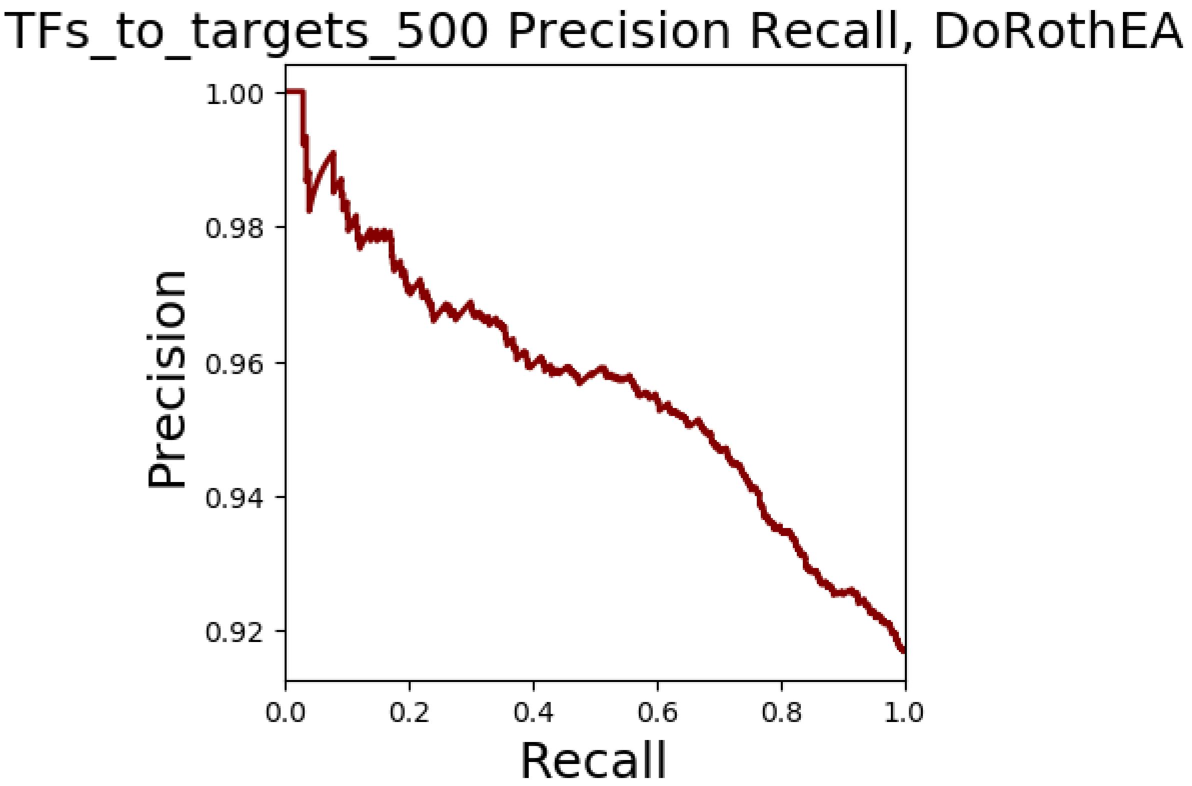

TFs_to_targets_500 Precision Recall, DoRothEA
Precision
1.00
0.98
0.96
0.94
0.92
0.0
0.2
0.4
0.6
0.8
1.0
Recall

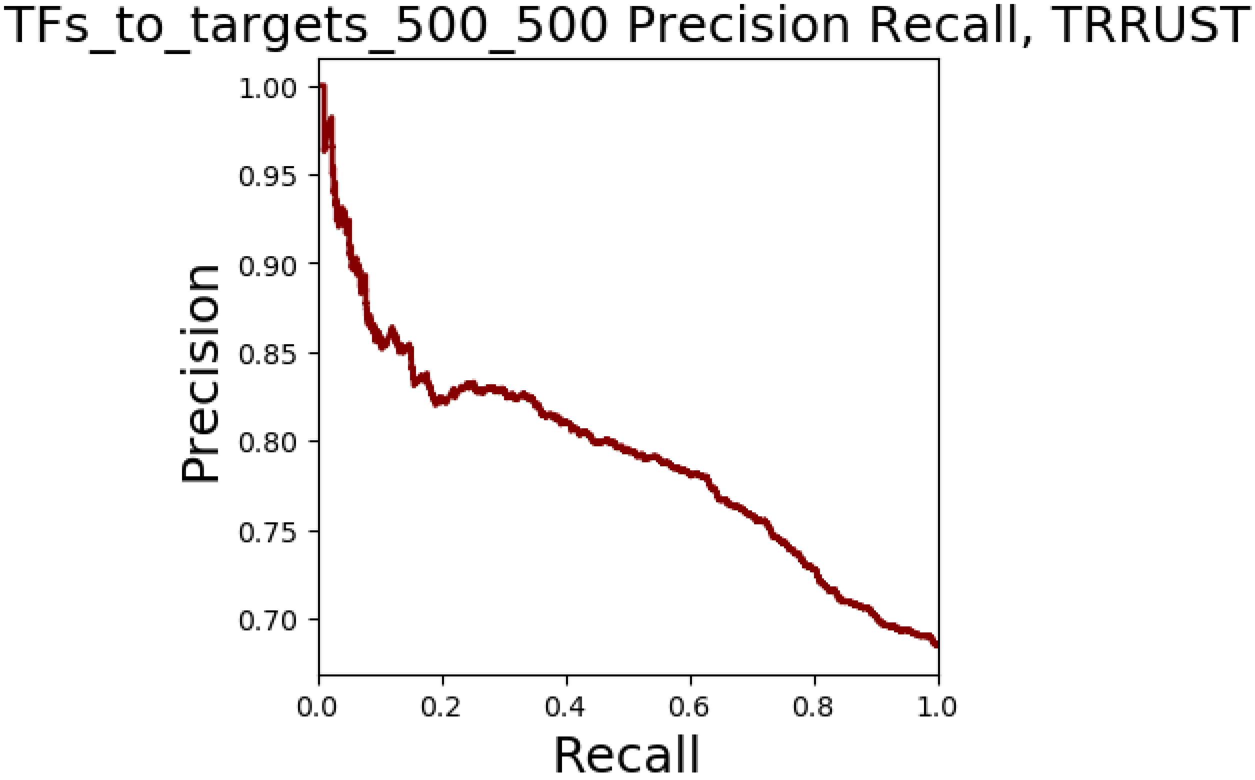

TFs_to_targets_500_500 Precision Recall, TRRUST
1.00
0.95
0.90
0.85
0.80
0.75
0.70
Precision
0.0
0.2
0.4
0.6
0.8
1.0
Recall

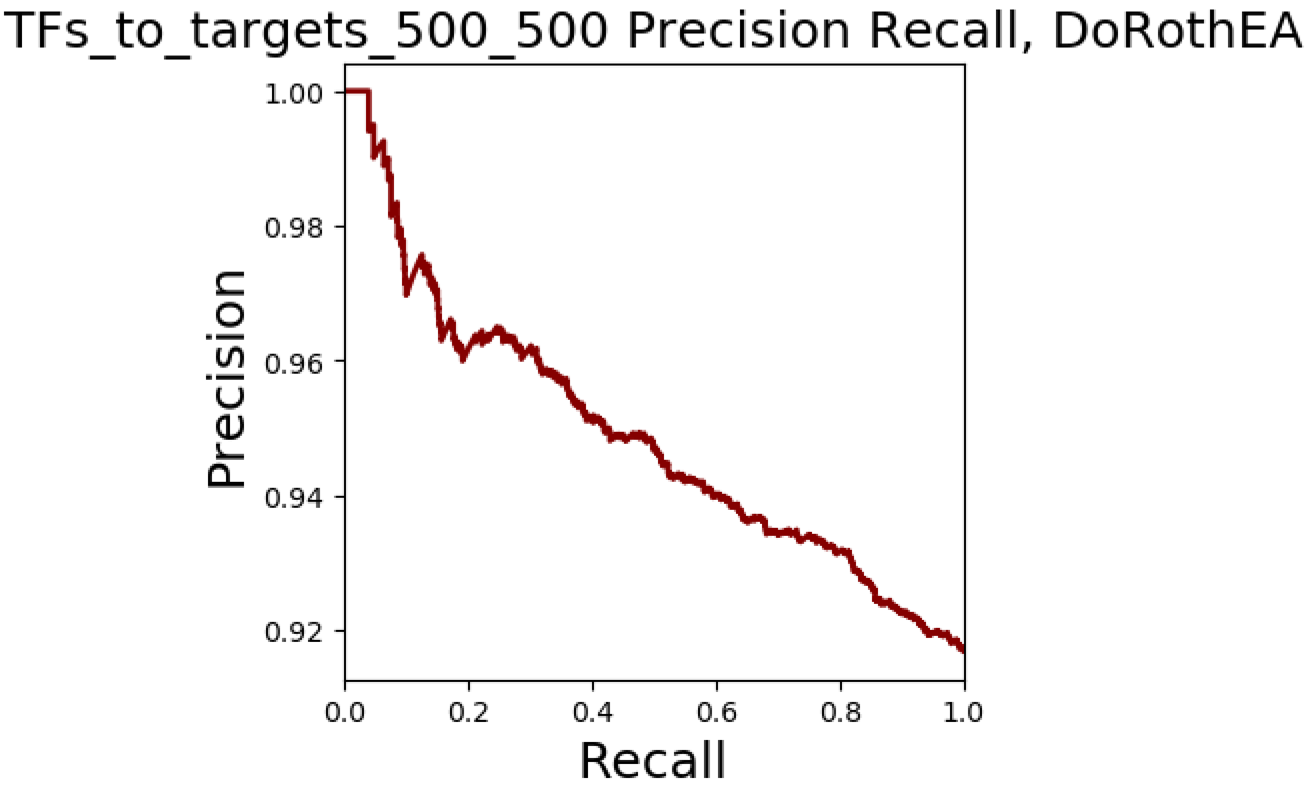

TFs_to_targets_500_500 Precision Recall, DoRothEA
Precision
1.00
0.98
0.96
0.94
0.92
Recall
0.0
0.2
0.4
0.6
0.8
1.0

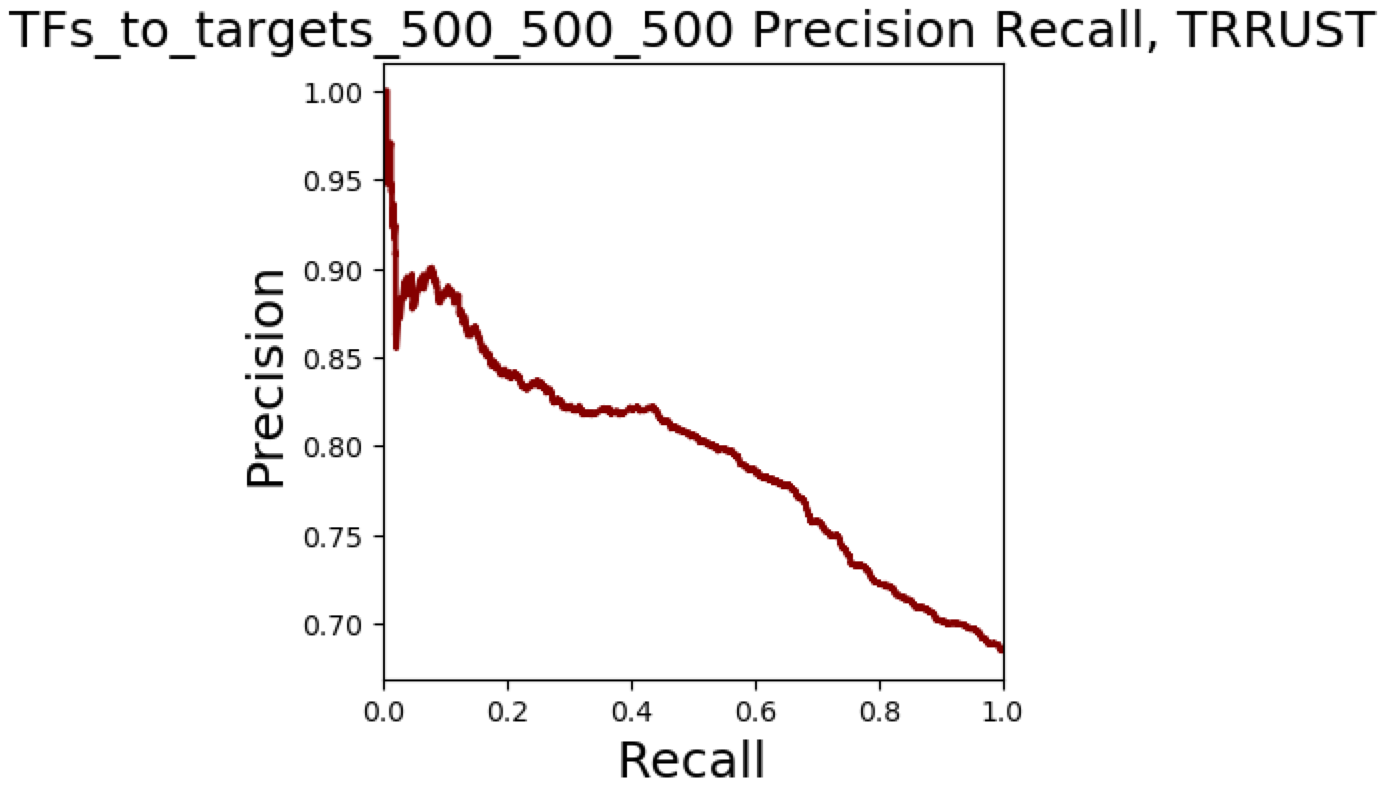
TFs_to_targets_500_500_500 Precision Recall, TRRUST
Precision
1.00
0.95
0.90
0.85
0.80
0.75
0.70
Recall
0.0
0.2
0.4
0.6
0.8
1.0

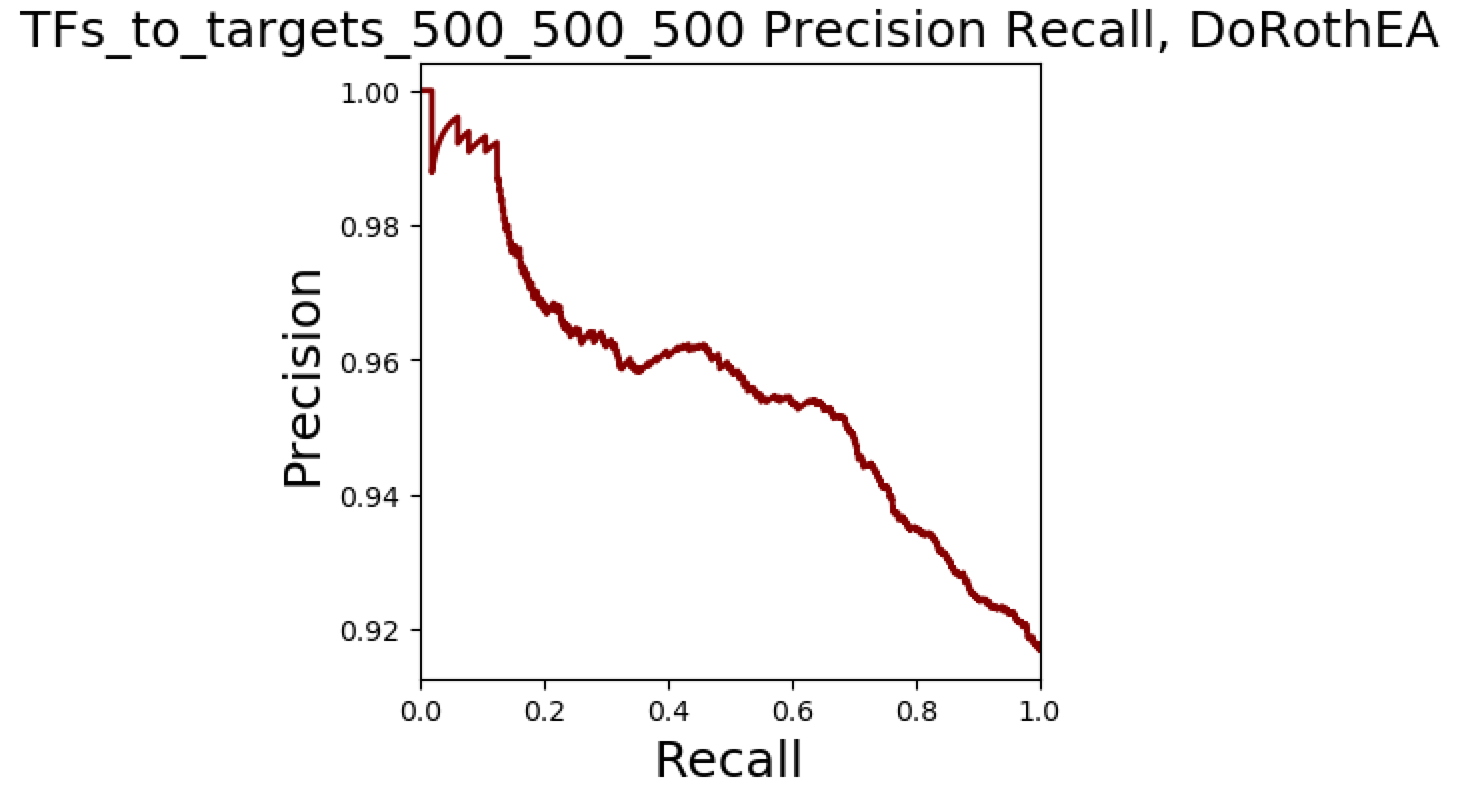
TFs_to_targets_500_500_500 Precision Recall, DoRothEA
Precision
Recall
1.00
0.98
0.96
0.94
0.92
0.0
0.2
0.4
0.6
0.8
1.0

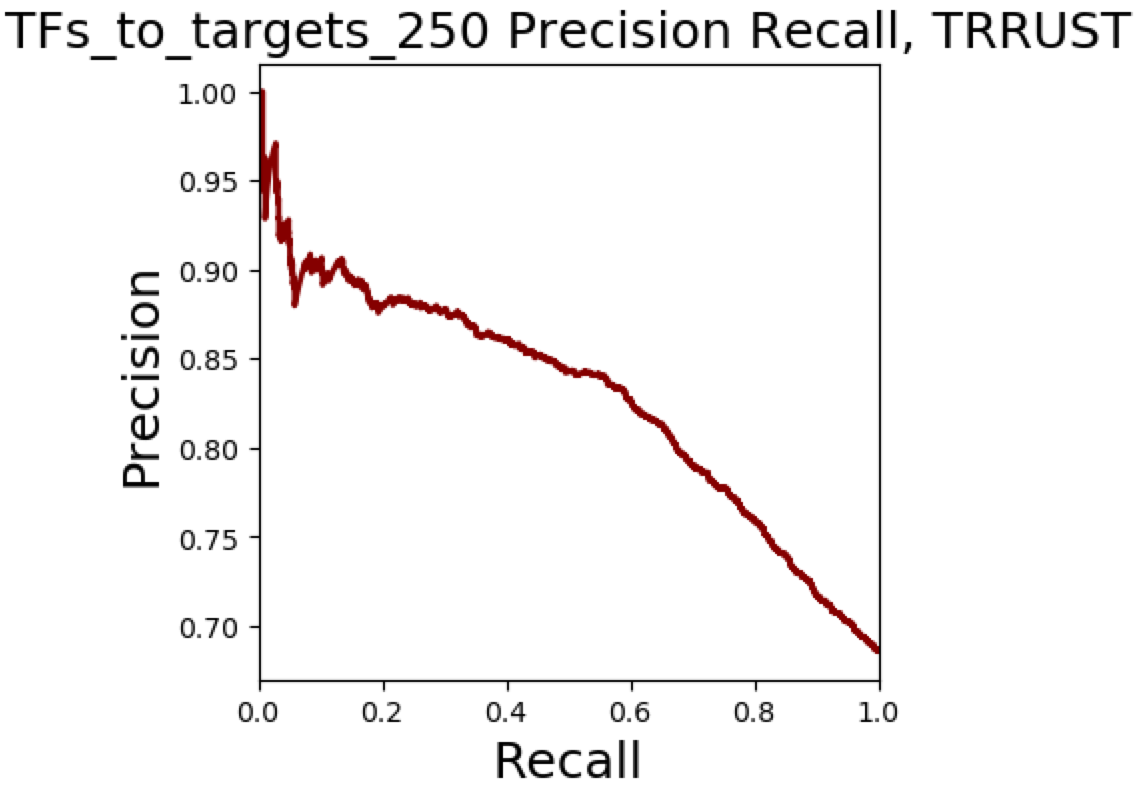
TFs_to_targets_250 Precision Recall, TRRUST
Precision
Recall
1.00
0.95
0.90
0.85
0.80
0.75
0.70
0.0
0.2
0.4
0.6
0.8
1.0

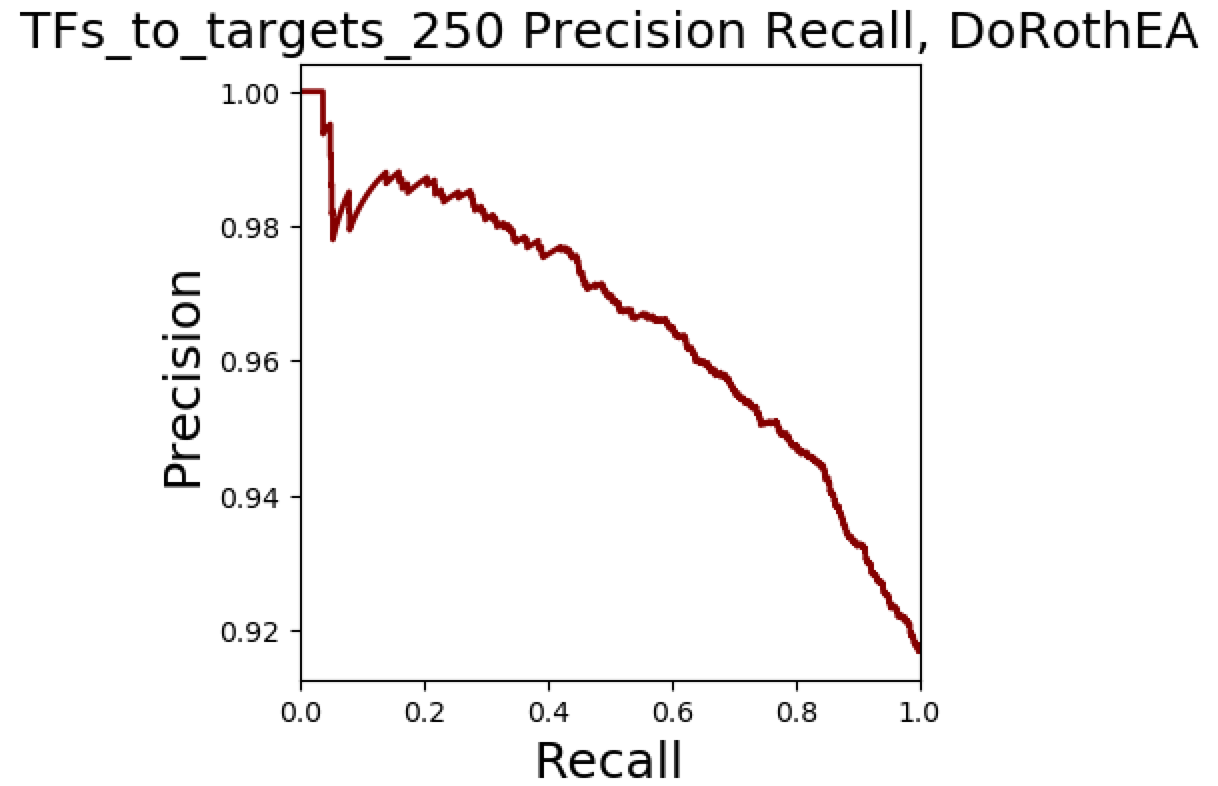
TFs_to_targets_250 Precision Recall, DoRothEA
Precision
1.00
0.98
0.96
0.94
0.92
Recall
0.0
0.2
0.4
0.6
0.8
1.0

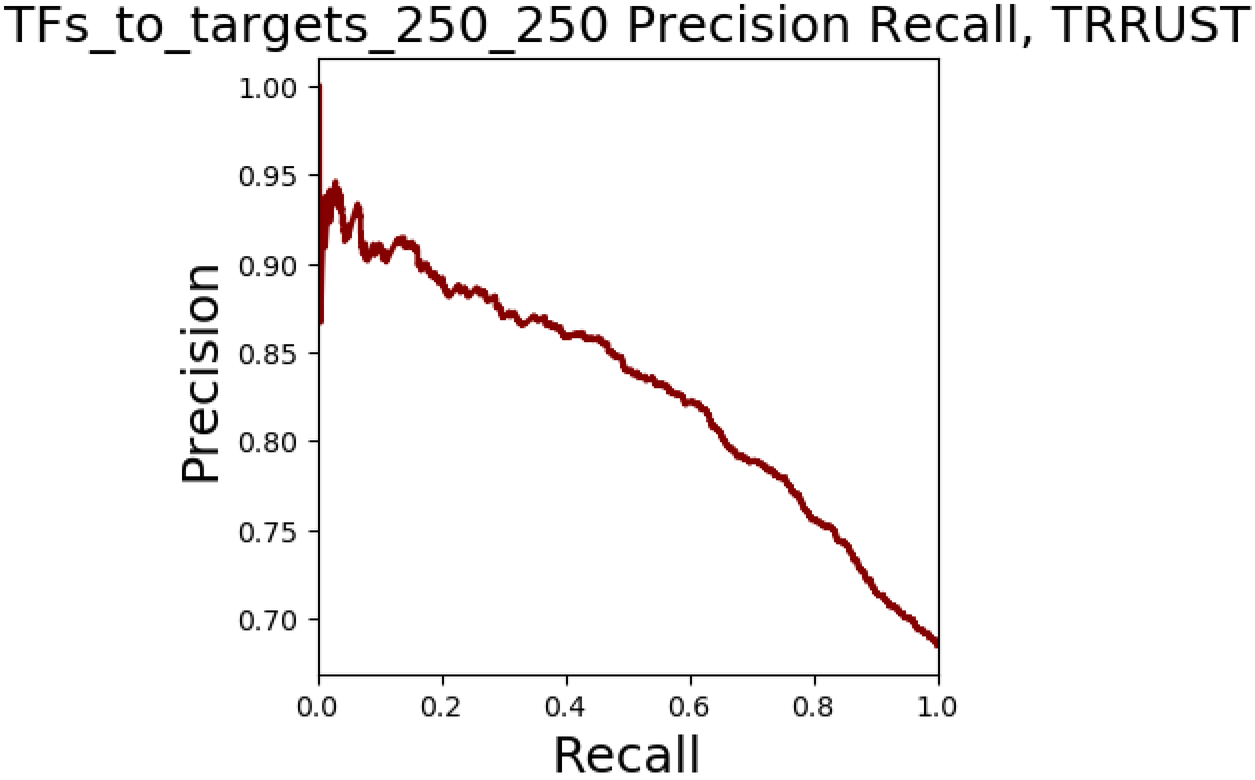

TFs_to_targets_250_250 Precision Recall, TRRUST
Precision
Recall
1.00
0.95
0.90
0.85
0.80
0.75
0.70
0.0
0.2
0.4
0.6
0.8
1.0

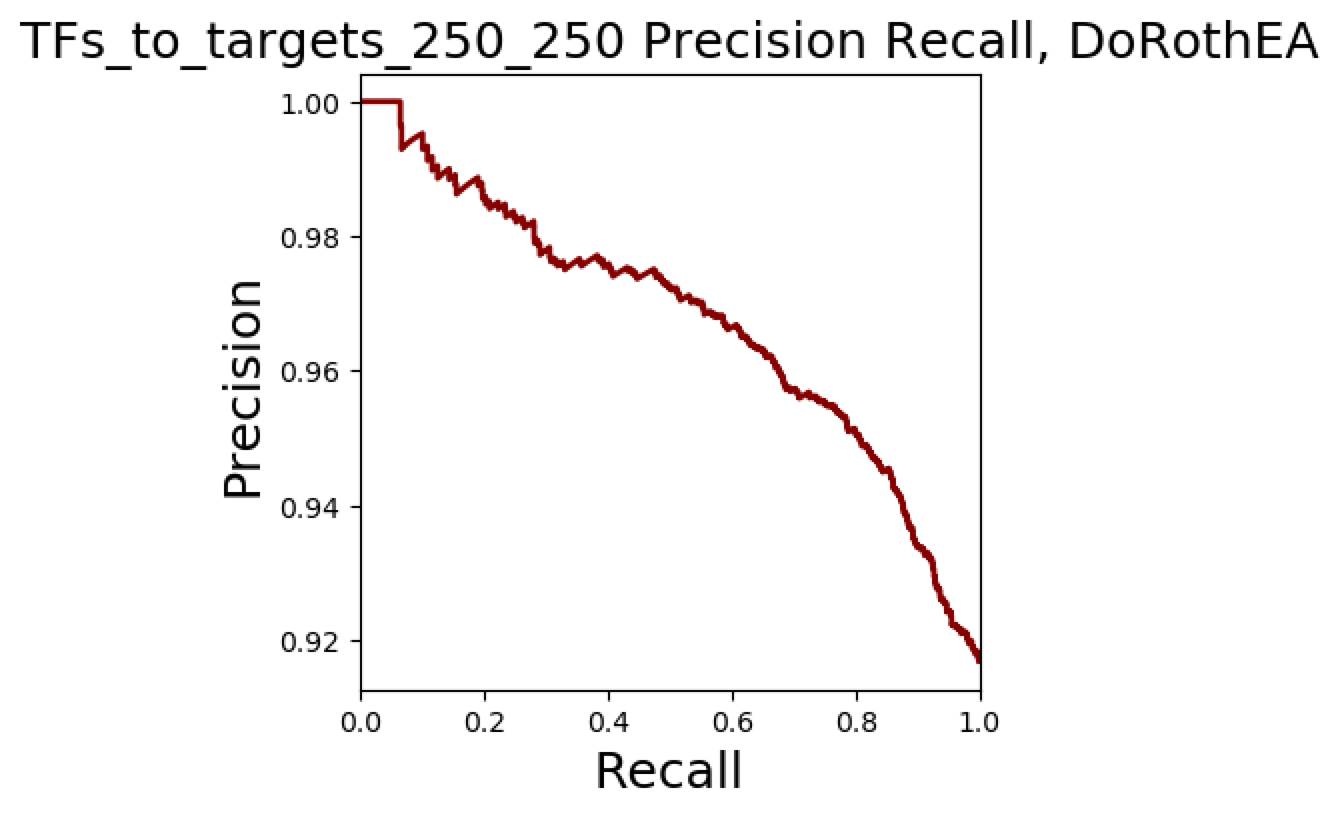
TFs_to_targets_250_250 Precision Recall, DoRothEA
Precision
Recall
1.00
0.98
0.96
0.94
0.92
0.0
0.2
0.4
0.6
0.8
1.0

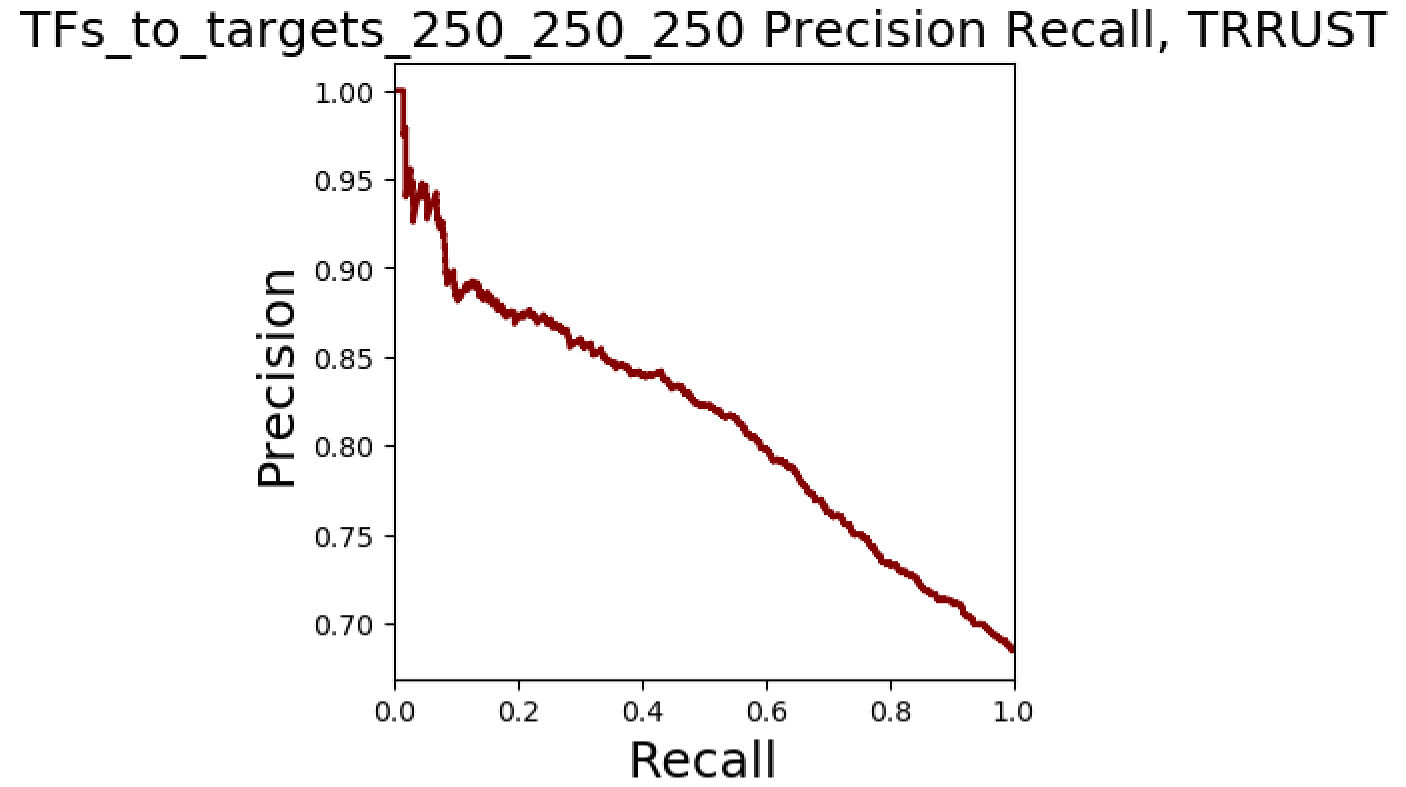
TFs_to_targets_250_250_250 Precision Recall, TRRUST
Precision
Recall
1.00
0.95
0.90
0.85
0.80
0.75
0.70
0.0
0.2
0.4
0.6
0.8
1.0

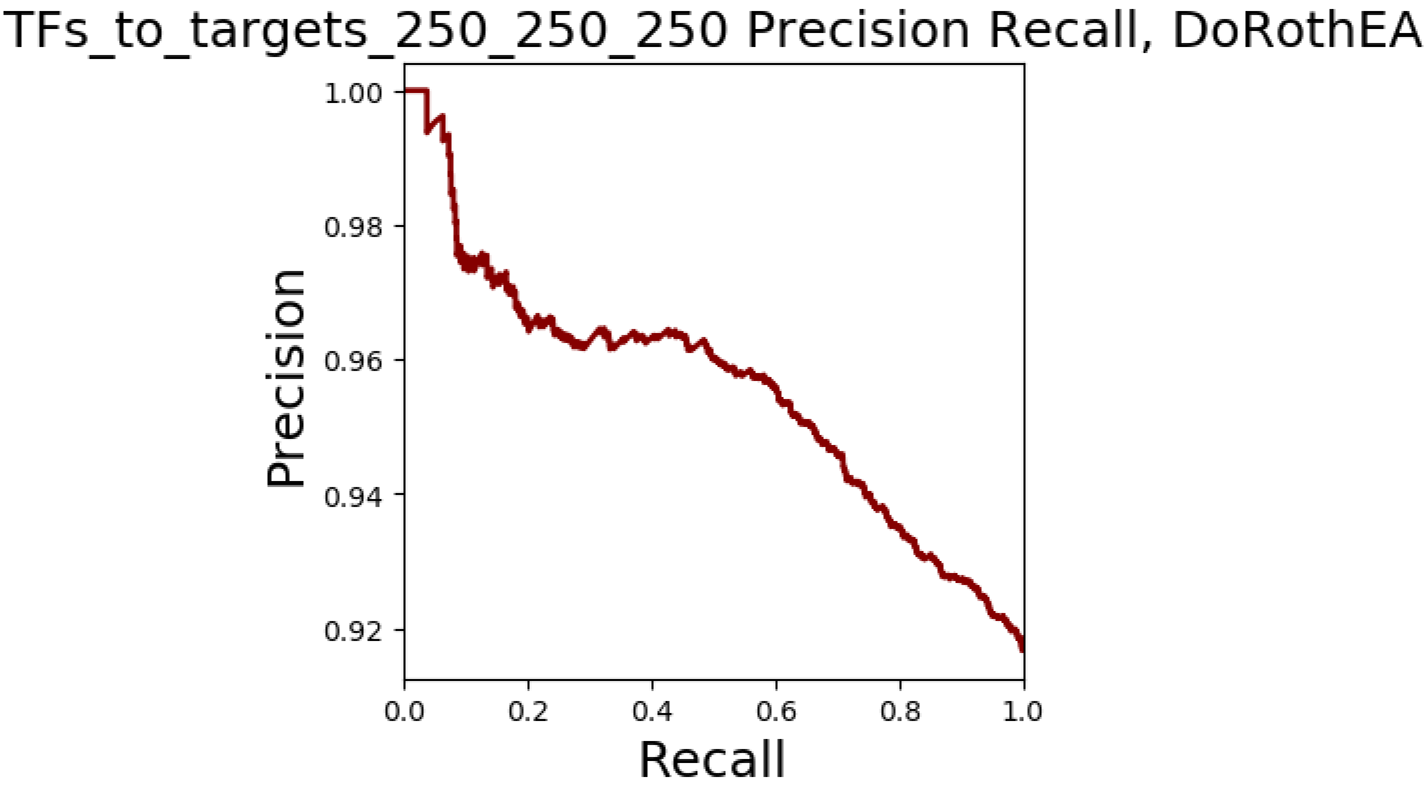
TFs_to_targets_250_250_250 Precision Recall, DoRothEA
Precision
1.00
0.98
0.96
0.94
0.92
Recall
0.0
0.2
0.4
0.6
0.8
1.0

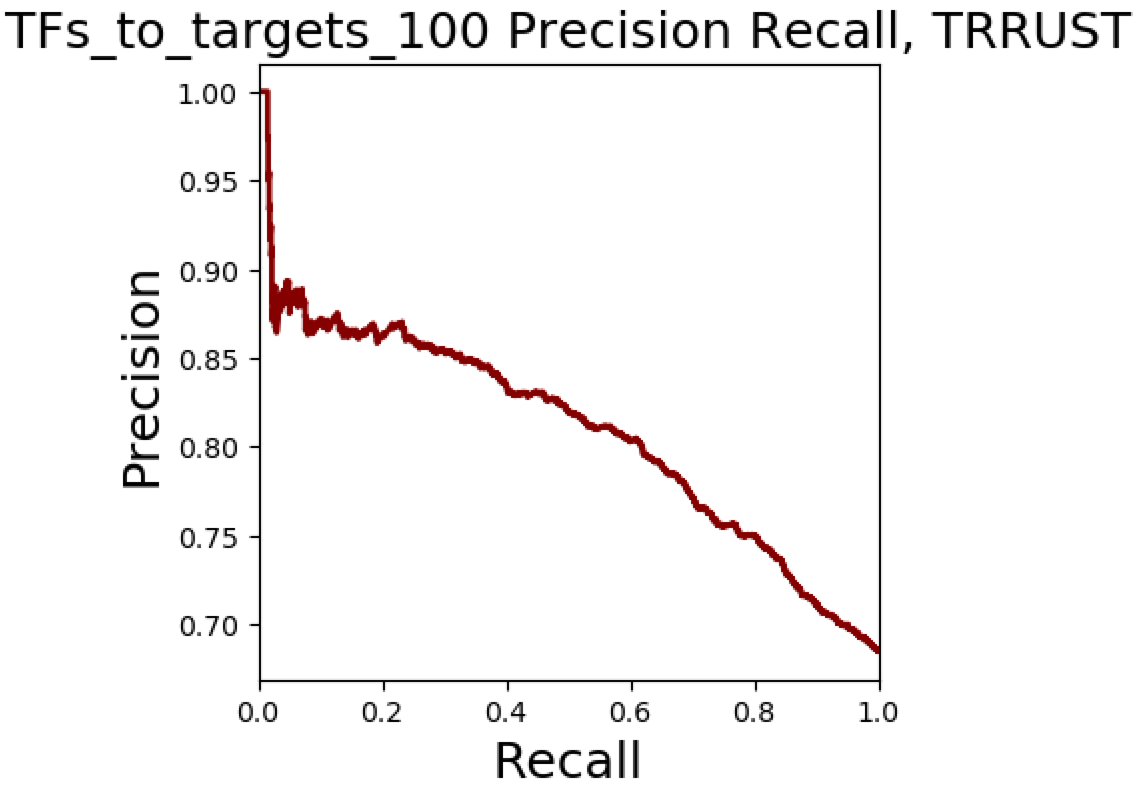
TFs_to_targets_100 Precision Recall, TRRUST
Precision
Recall
1.00
0.95
0.90
0.85
0.80
0.75
0.70
0.0
0.2
0.4
0.6
0.8
1.0

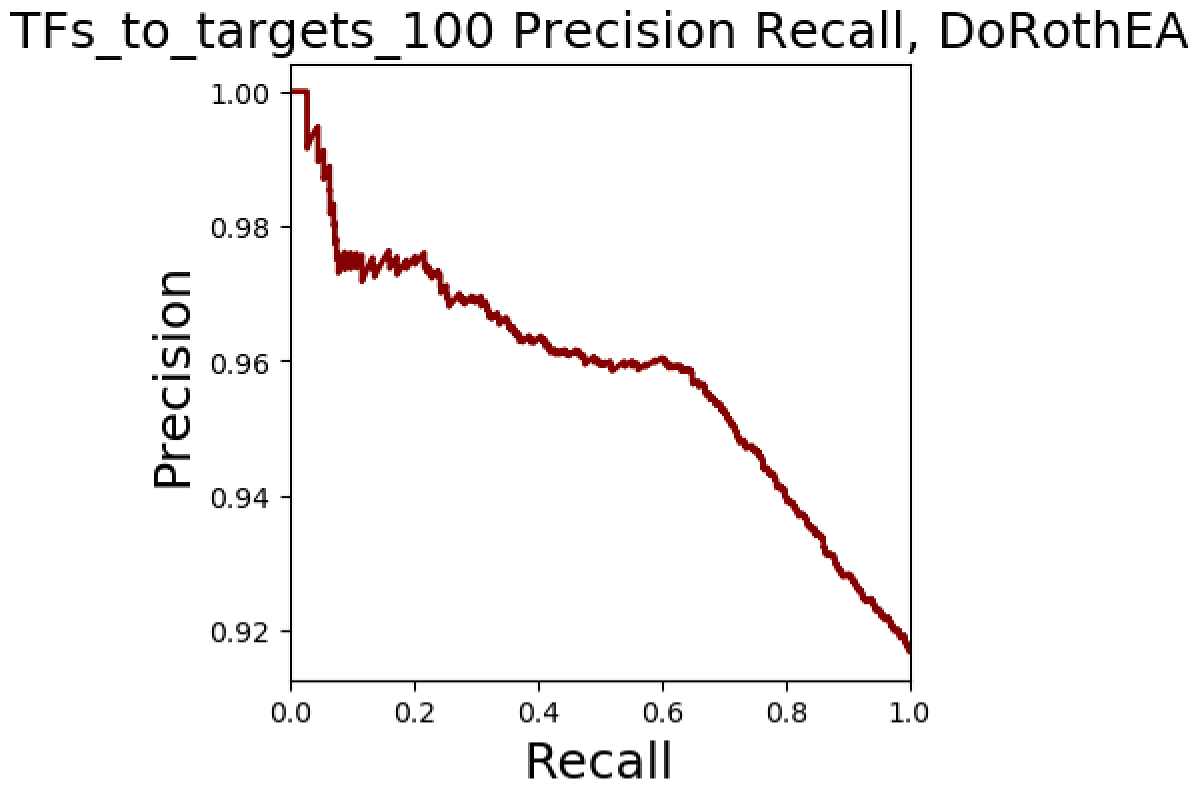
TFs_to_targets_100 Precision Recall, DoRothEA
Precision
Recall
1.00
0.98
0.96
0.94
0.92
0.0
0.2
0.4
0.6
0.8
1.0

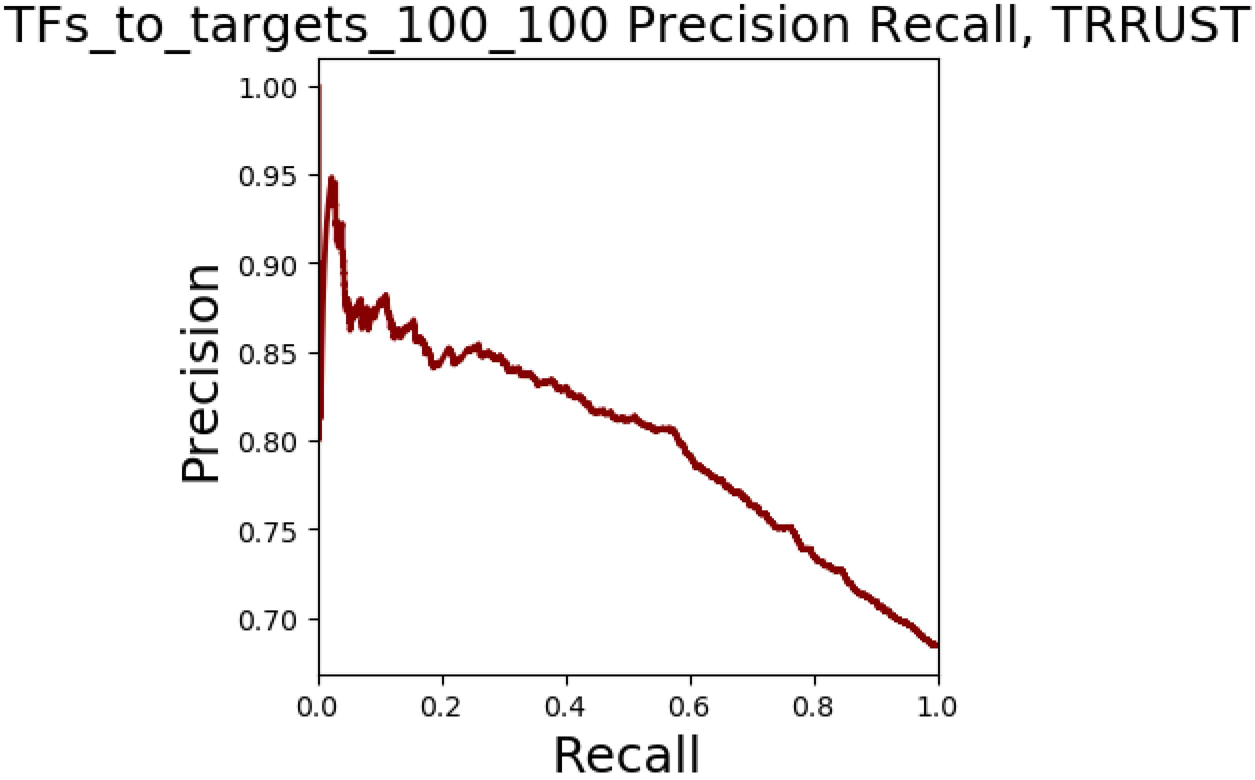
TFs_to_targets_100_100 Precision Recall, TRRUST
Precision
1.00
0.95
0.90
0.85
0.80
0.75
0.70
0.0
0.2
0.4
0.6
0.8
1.0
Recall

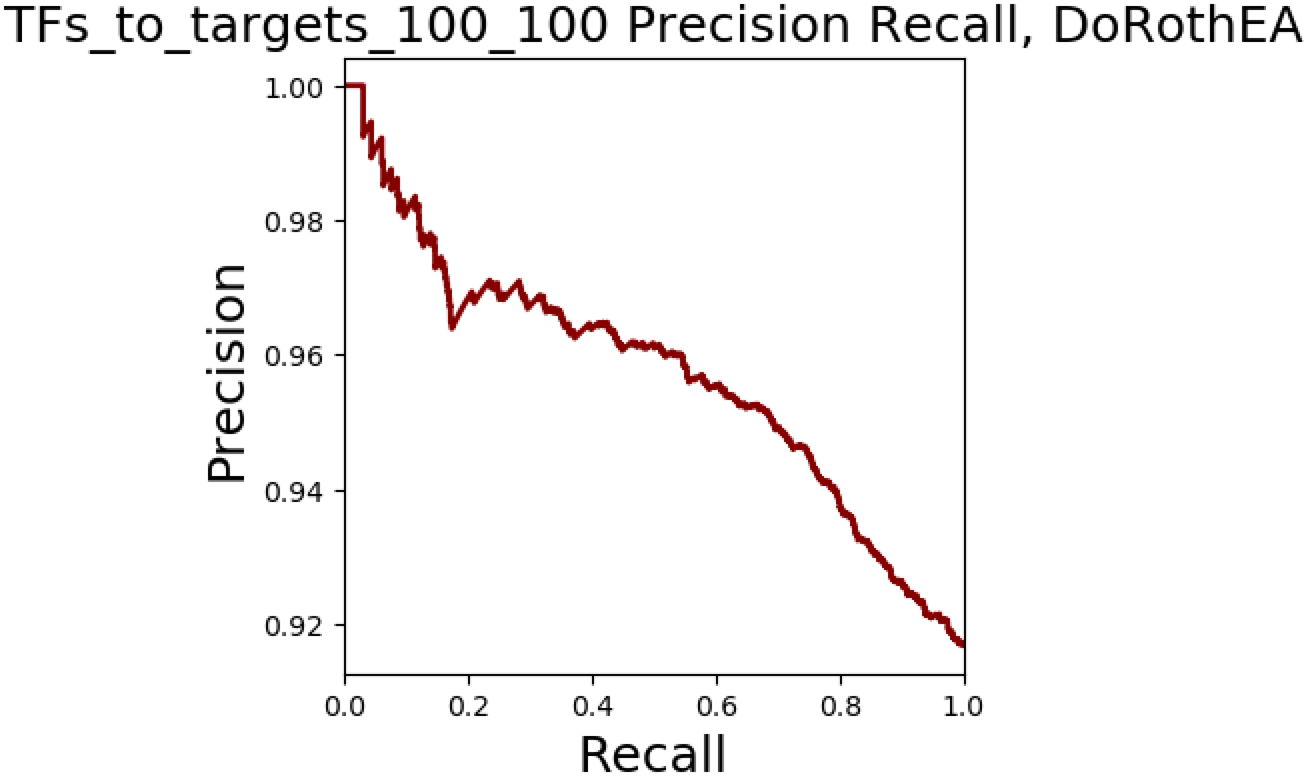
TFs_to_targets_100_100 Precision Recall, DoRothEA
Precision
1.00
0.98
0.96
0.94
0.92
0.0
0.2
0.4
0.6
0.8
1.0
Recall

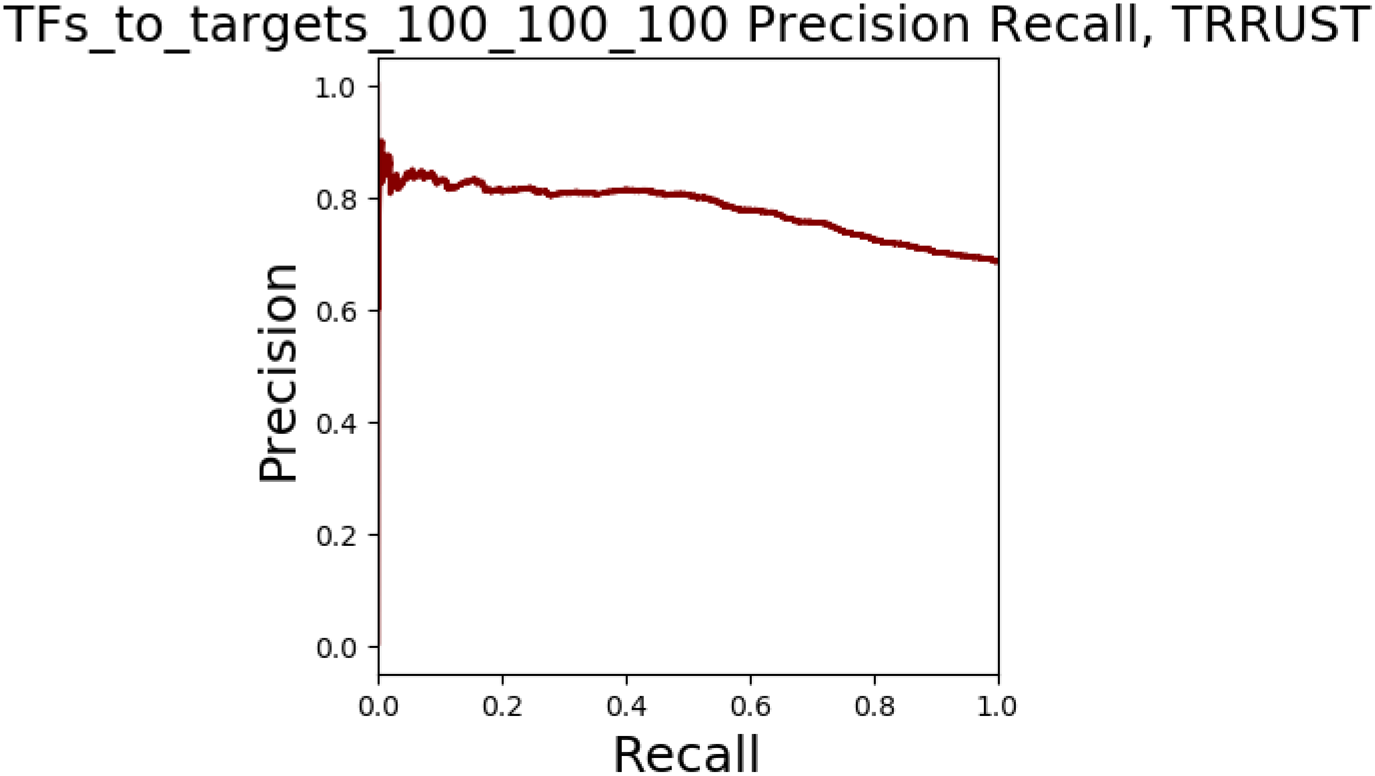
TFs_to_targets_100_100_100 Precision Recall, TRRUST
Precision
Recall
0.0
0.2
0.4
0.6
0.8
1.0

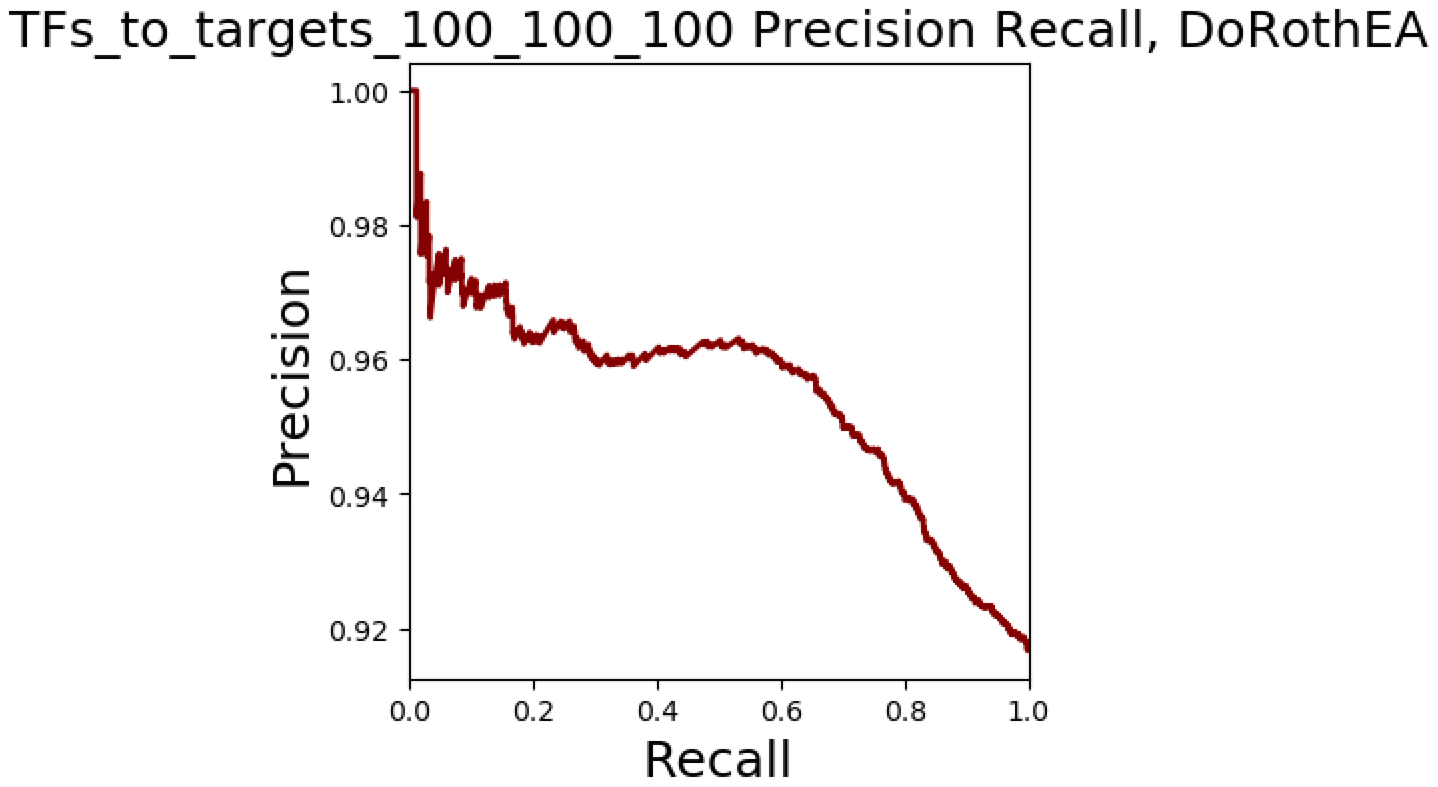
TFs_to_targets_100_100_100 Precision Recall, DoRothEA
Precision
1.00
0.98
0.96
0.94
0.92
Recall
0.0
0.2
0.4
0.6
0.8
1.0

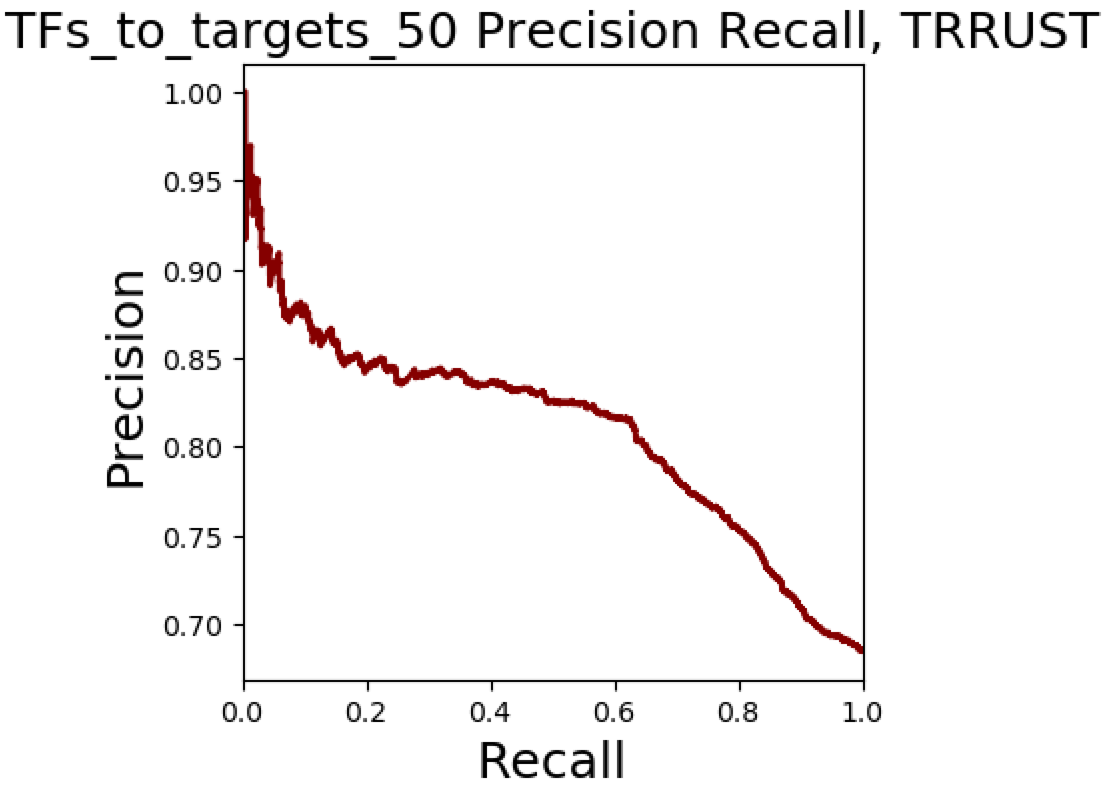
TFs_to_targets_50 Precision Recall, TRRUST
Precision
Recall
1.00
0.95
0.90
0.85
0.80
0.75
0.70
0.0
0.2
0.4
0.6
0.8
1.0

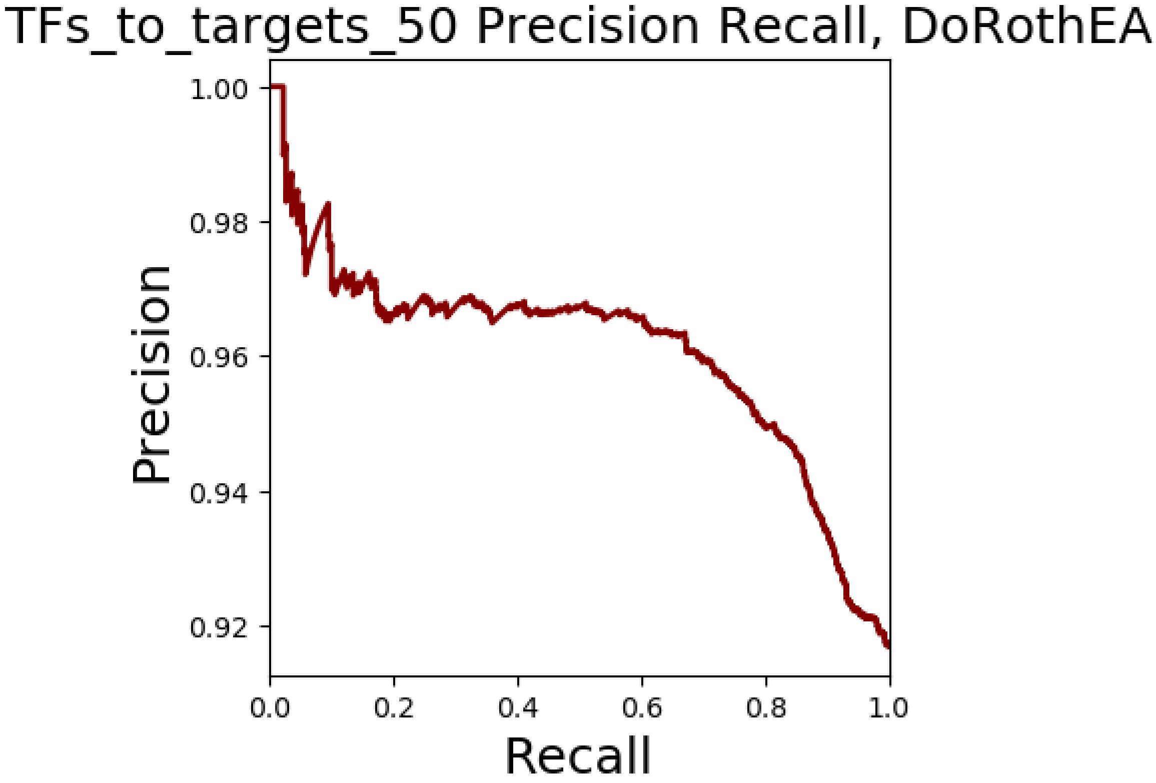

TFs_to_targets_50 Precision Recall, DoRothEA
Precision
Recall
1.00
0.98
0.96
0.94
0.92
0.0
0.2
0.4
0.6
0.8
1.0

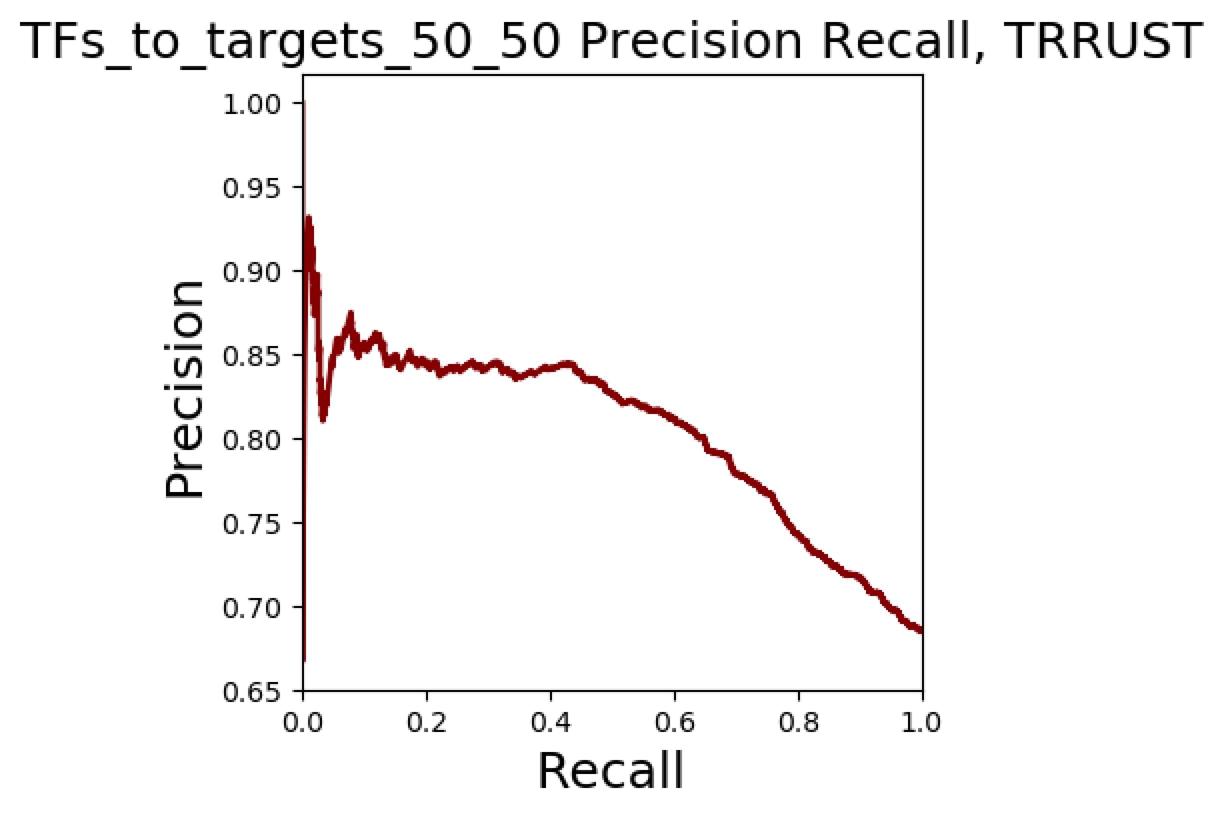
TFs_to_targets_50_50 Precision Recall, TRRUST
Precision
Recall
1.00
0.95
0.90
0.85
0.80
0.75
0.70
0.65
0.0
0.2
0.4
0.6
0.8
1.0

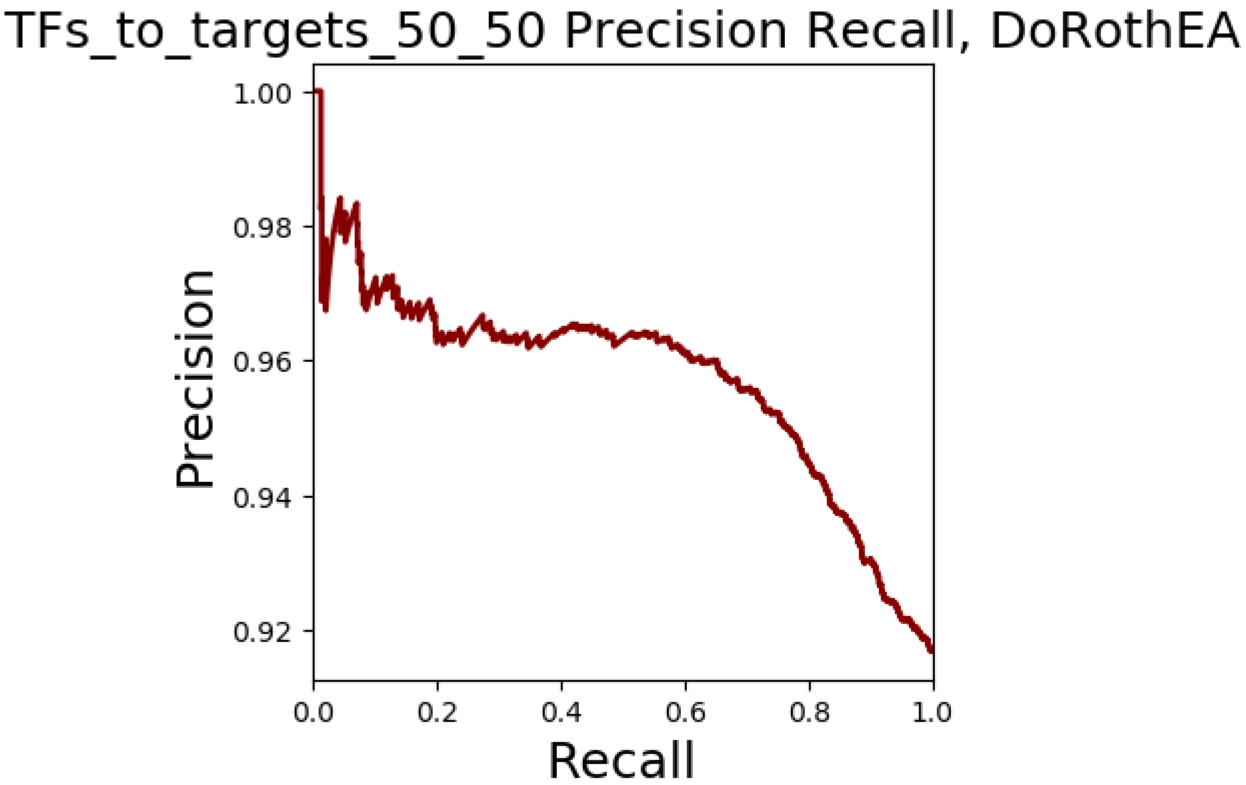
TFs_to_targets_50_50 Precision Recall, DoRothEA
Precision
1.00
0.98
0.96
0.94
0.92
Recall
0.0
0.2
0.4
0.6
0.8
1.0

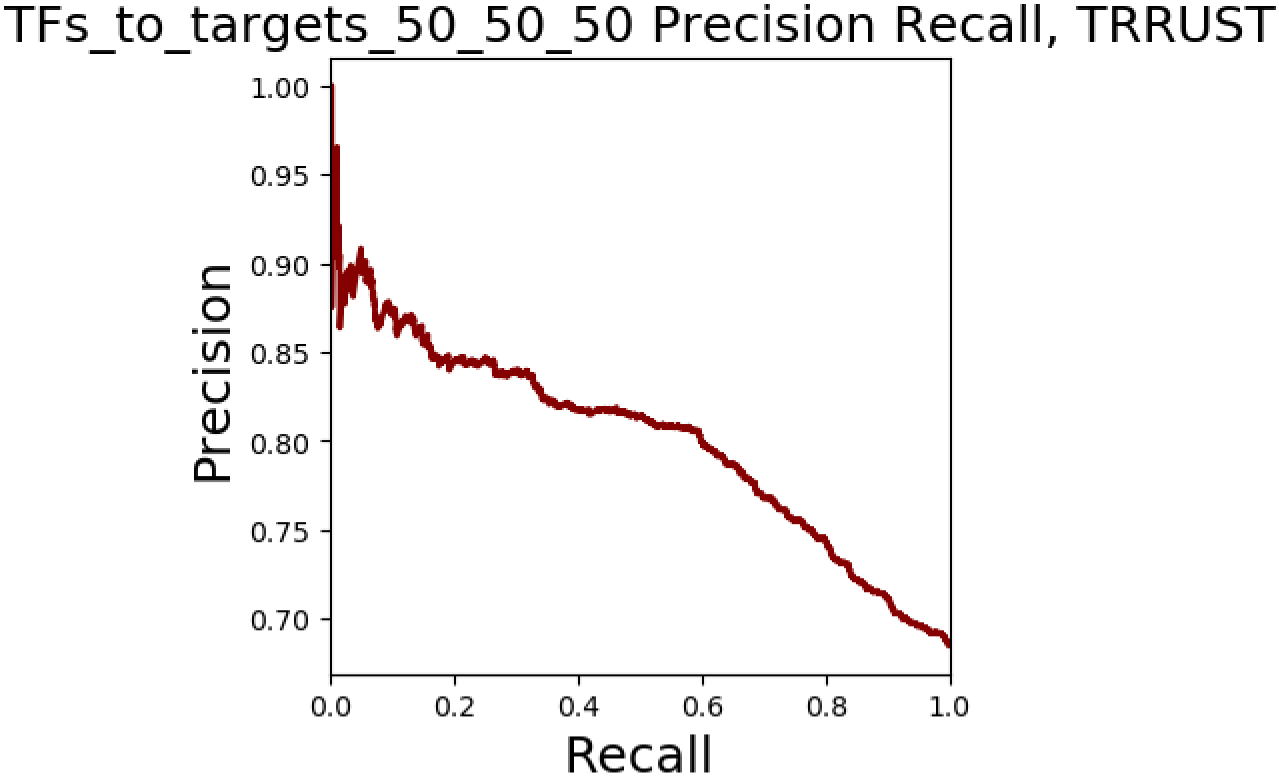
TFs_to_targets_50_50_50 Precision Recall, TRRUST
Precision
1.00
0.95
0.90
0.85
0.80
0.75
0.70
0.0
0.2
0.4
0.6
0.8
1.0
Recall

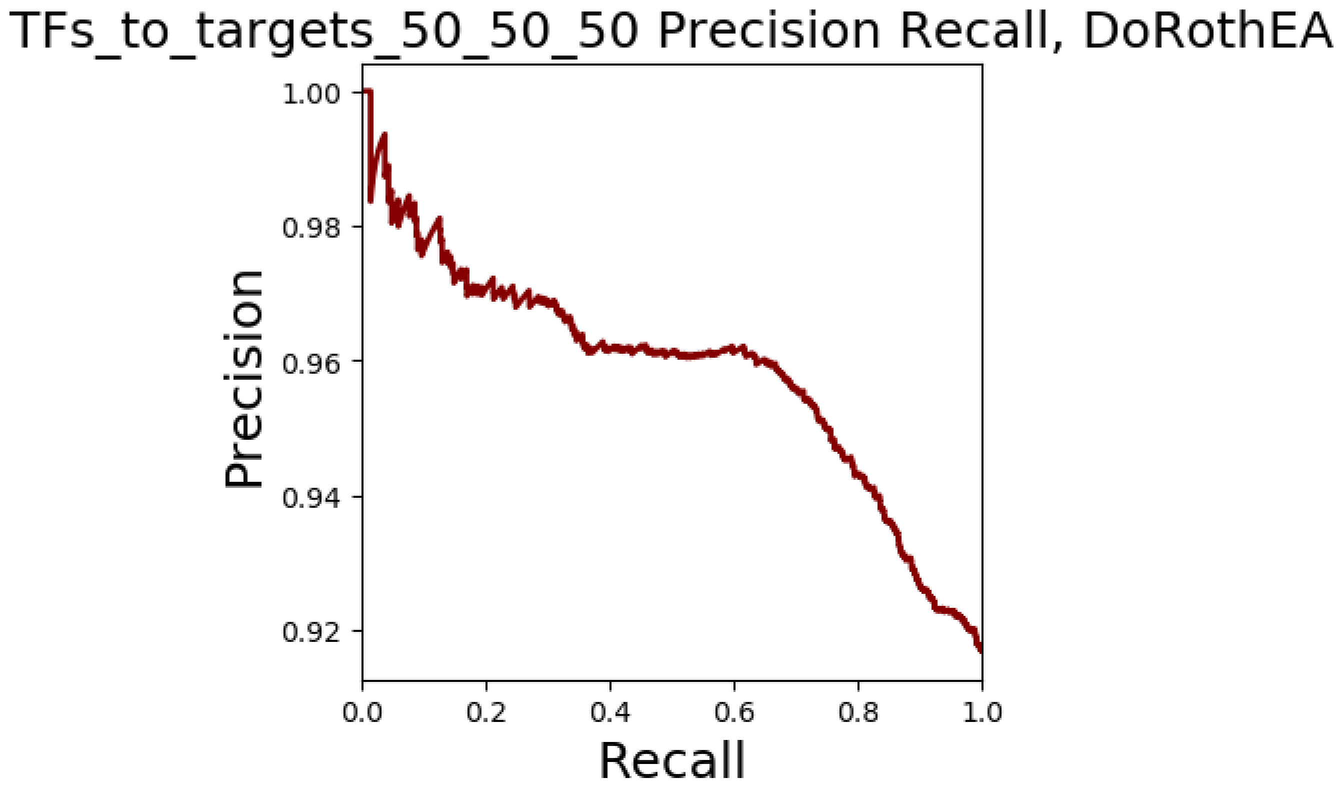

TFs_to_targets_50_50_50 Precision Recall, DoRothEA
Precision
1.00
0.98
0.96
0.94
0.92
0.0
0.2
0.4
0.6
0.8
1.0
Recall

Supplementary material 3: Hierarchical structures of TF inputs are robust between models

For each of the models, we performed a backward-selection algorithm on the TFs (Methods) to rank the TFs according to importance when predicting target gene expression. These models were trained completely independently, and to analyse how robust TF-to-target interactions identified by the models were, i.e. how consistent the model approach was, we compared the similarities of removal order between the models. We measured similarity as correlation between the respective step at which each TF was removed, with one correlation between each model pair (Supplementary Figure 1).

We found that the models that had a high compression, i.e. few hidden nodes in each layer, were less similar in the order TFs were removed. Nevertheless, all TF-ranking comparisons had highly significant Spearman correlations (highest P < 10e-118, rho=0.53).

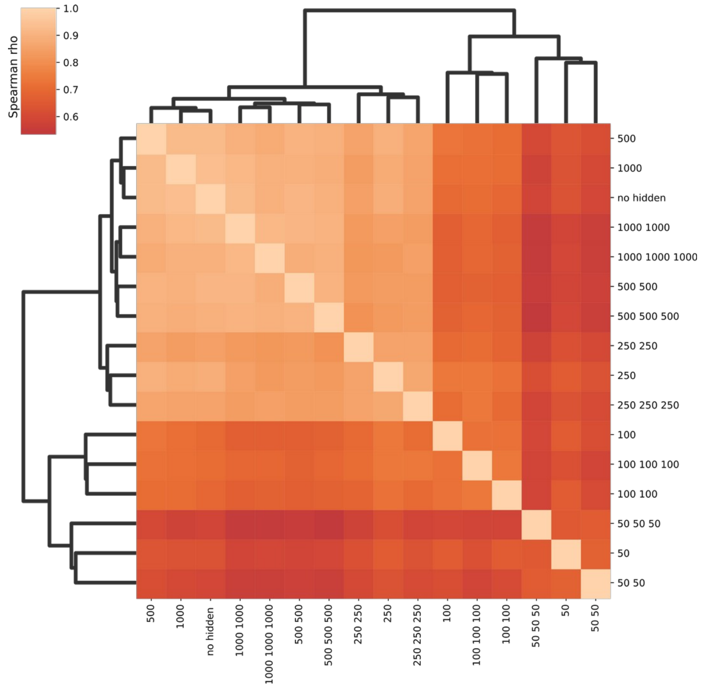

Supplementary Figure 1: *Consistency of TF-removals between models. We found the iteration at which TF was removed to be highly consistent between models, indicating a robustness in how TFs are used to predict target genes.*
